# Imide Condensation as a Strategy for the Synthesis of Core‐Diversified G‐Quadruplex Ligands with Anticancer and Antiparasitic Activity**

**DOI:** 10.1002/chem.202100040

**Published:** 2021-05-02

**Authors:** Steven T. G. Street, Pablo Peñalver, Michael P. O'Hagan, Gregory J. Hollingworth, Juan C. Morales, M. Carmen Galan

**Affiliations:** ^1^ School of Chemistry University of Bristol Cantocks Close Bristol BS8 1TS UK; ^2^ Department of Chemistry University of Victoria Dr. S. T. G. Street Victoria BC V8P 5C2 Canada; ^3^ Instituto de Parasitología y Biomedicina López Neyra CSIC, PTS Granada Avenida del Conocimiento, 17 18016 Armilla, Granada Spain; ^4^ Novartis Institutes for Biomedical Research Novartis Campus 4002 Basel Switzerland

**Keywords:** Aggregation, Amphiphiles, Antiprotozoal agents, Drug design, G-quadruplexes

## Abstract

A facile imide coupling strategy for the one‐step preparation of G‐quadruplex ligands with varied core chemistries is described. The G‐quadruplex stabilization of a library of nine compounds was examined using FRET melting experiments, and CD, UV‐Vis, fluorescence and NMR titrations, identifying several compounds that were capable of stabilizing G‐quadruplex DNA with interesting selectivity profiles. The best G4 ligand was identified as compound **3**, which was based on a perylene scaffold and exhibited 40‐fold selectivity for a telomeric G‐quadruplex over duplex DNA. Surprisingly, a tetra‐substituted flexible core, compound **11**, also exhibited selective stabilization of G4 DNA over duplex DNA. The anticancer and antiparasitic activity of the library was also examined, with the lead compound **3** exhibiting nanomolar inhibition of *Trypanosoma brucei* with 78‐fold selectivity over MRC5 cells. The cellular localization of this compound was also studied via fluorescence microscopy. We found that uptake was time dependant, with localization outside the nucleus and kinetoplast that could be due to strong fluorescence quenching in the presence of small amounts of DNA.

## Graphical abstract

**The rigid rules of G‐quadruplex stabilization**: A library of amphiphiles has been synthesized in one step, using a simple imide condensation reaction. The G‐quadruplex stabilization, anti‐cancer and anti‐parasitic activity of the library has been investigated, uncovering the important factors that govern G‐quadruplex binding and selectivity, yielding a promising new core‐motif and a compound with potent trypanosomacidal activity.

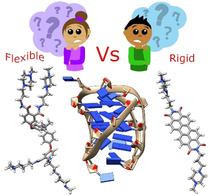

## Introduction

G‐quadruplexes (G4s) are a class of secondary nucleic acid structures formed from guanine‐rich sequences. These motifs have garnered significant attention in recent years, as a wide variety of potential applications and functions continue to emerge.^[1]^ Since the discovery of the first G4 DNA stabilizing ligand by Neidle and Hurley *et. al*. in 1997^[2]^ the field of G4 interactive compounds has experienced exponential growth.^[3]^ Advances in our understanding of G4 biology have driven new discoveries of the potential biomedical applications of G4 ligands in the therapeutic targeting of numerous diseases.^[4]^ Whilst much attention has focused on the use of this strategy for the treatment of cancer,^[5]^ recently their potential to be deployed as antiparasitic agents has come to light.^[6]^ The stabilization of key G4‐forming sequences in gene promoters is proposed to prevent the binding of transcription factors and lead to downstream gene silencing and toxic effects. Induction of the G4 motif at chromosome telomeres is also demonstrated to induce toxicity via inhibition of telomerase, a known anticancer target.

Whilst numerous small molecules have been reported to interact with G4 DNA for use as therapeutics,^[7]^ only a handful have been investigated clinically^[8]^ due to challenging syntheses, poor bioavailability, and poor target specificity.^[7c]^ Whilst significant progress has been made, it is clear that efficient methods to rapidly generate diverse libraries of potential G4 ligands are needed to provide us with a better understanding of the parameters that govern G4 stabilization, destabilization, and target selectivity, which are key requirements for the development of clinically viable G4 interactive small molecules.

**Figure 1:**
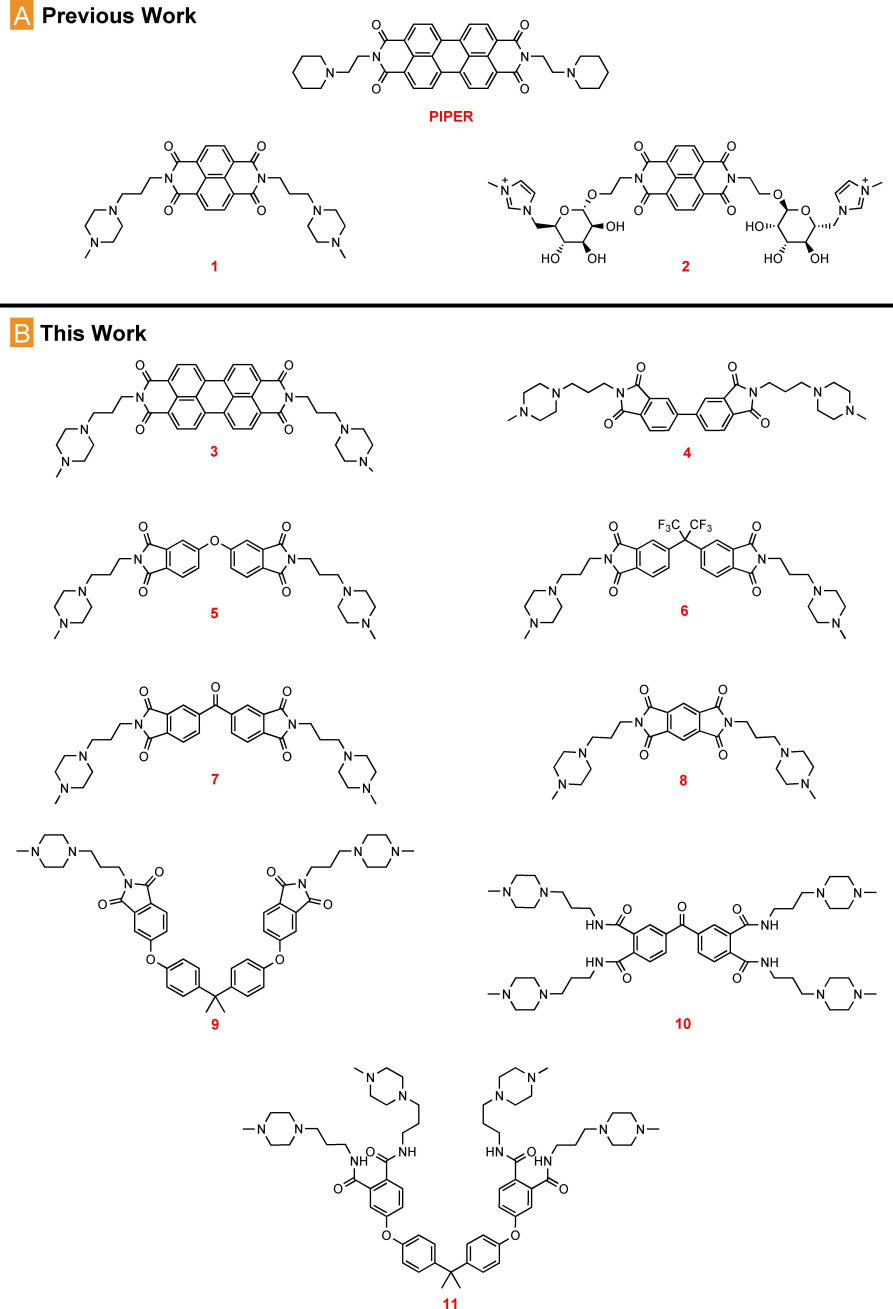
Compounds studied in this work, utilizing imide‐based coupling of a variety of commercially available building blocks. (A) Lead compounds **1** and **2** from previous work,^[9]^ and control G4 ligand **PIPER**.^[12]^ (B) New compounds **3**–**11** studied in this work, probing the role of core flexibility, aromaticity, and electronics on G‐quadruplex binding.

Recent efforts in our groups have been focused on the development of a series of G4 targeting compounds derived from the naphthalene diimide (NDI) scaffold.^[9]^ We found that di‐substituted diimides, particularly NDI‐glycoconjugates, displayed interesting selectivity profiles towards G4 structures with lead compounds **1** and **2** (Figure 1, A) stabilizing particular G4 topologies with good discrimination. Moreover, compound **1** exhibited submicromolar activity against cancerous cell lines. Though NDI cores have been extensively studied as the basis of G4‐targeting molecules,^[10]^ to date there has been no systematic exploration of different diimide scaffolds to probe the role of the ligand core in driving G4 binding, and to identify new core‐forming motifs. In this work, we utilize the simplicity of imide coupling chemistry to develop, in one step, a library of functional, amphiphilic small molecules with a variety of different cores from commercially available materials. The work herein probes the role of core flexibility, structure, and electronics in facilitating G4 binding, and examines whether ligand G4 stabilisation is correlated with antiparasitic or anticancer activity *in vitro*.

## Results and Discussion

**Scheme 1:**
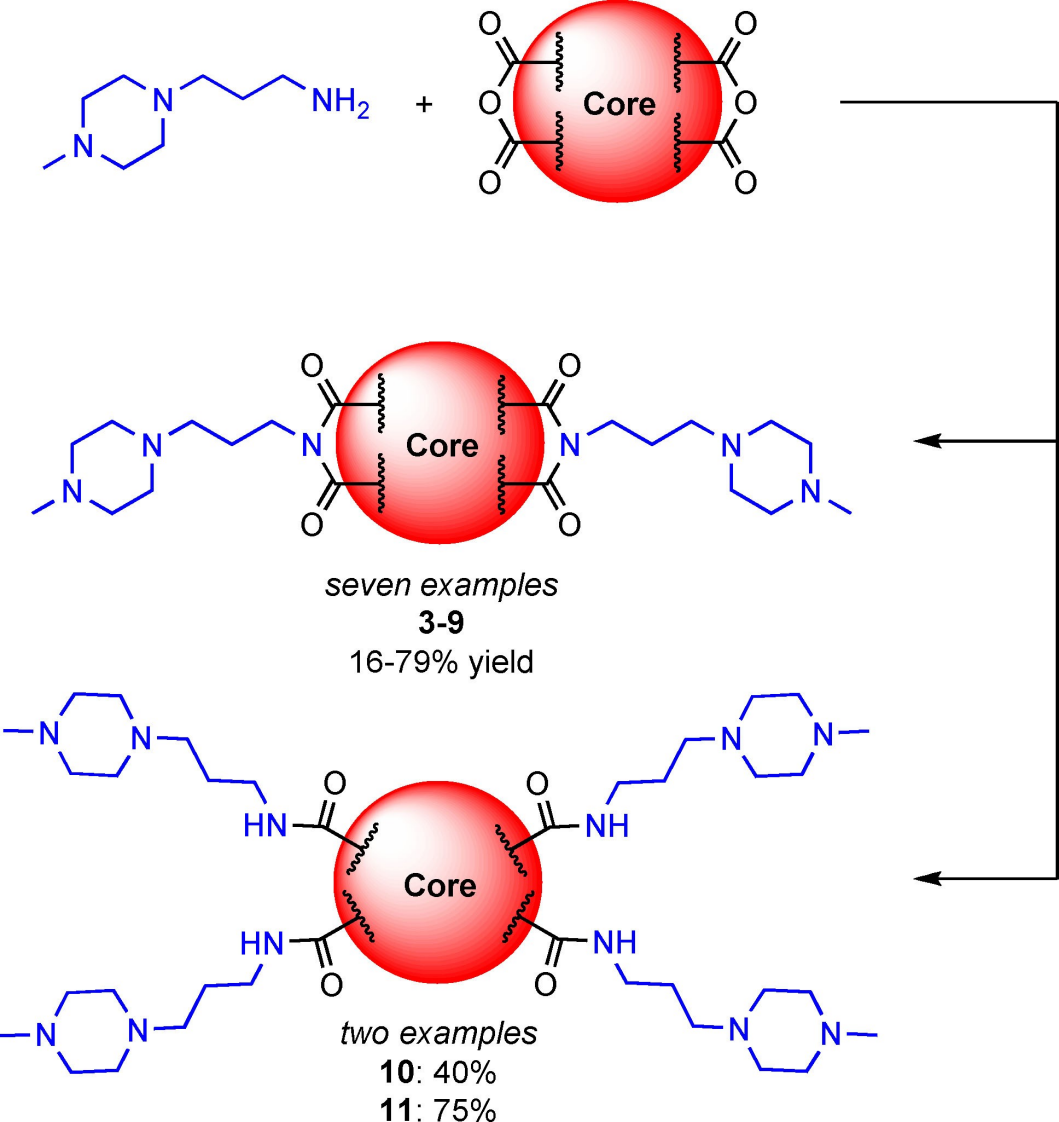
The one‐step synthesis of the nine compounds studied in this work. Conditions were: toluene, 145 °C for **4**–**8** and **10**–**11**; imidazole, 145 °C for **3** and DMSO, NEt_3_, 80 °C for **9**. For experimental details, see Supporting Information.

Previous work on G4 ligands^[11]^ indicates that 1‐(3‐aminopropyl)‐4‐methylpiperazine is an effective side chain that is capable of conferring G4 recognition properties and aqueous solubility to aromatic cores. We thus rationalized that coupling this readily‐available motif with a range of commercially available anhydride cores would yield a library of interesting amphiphiles in one step, allowing for fast diversification and generation of novel G4 binding motifs (Scheme 1). As a proof of concept, we started by examining seven different commercially available dianhydrides, which together probe a variety of features important for G4 binding, such as the flexibility of the central scaffold, the role of electronic and steric effects, conformational freedom, and the number and presentation of the aromatic rings. Nine new compounds were produced in total (Figure 1, B). The imide coupling reaction is compatible with a wide range of solvents; good results were obtained in toluene for many of the dianhydrides examined, whilst imidazole was used as the solvent for the synthesis of perylene diimide (PDI) **3**, due to the limited solubility of perylene‐3,4,9,10‐tetracarboxylic dianhydride. Purification of the resulting compounds by reverse‐phase flash chromatography (water+0.1 % trifluoroacetate (TFA)/MeCN, 9 : 1 to 5 : 95) was sufficient to protonate the methylpiperazine rings, yielding the final compounds as water soluble TFA salts.

Compounds **3**–**9** were prepared in yields ranging from 16–79 % as di‐substituted bisimides, while tetra‐substituted compounds **10** and **11** were obtained serendipitously in the presence of an excess of amine in 40 and 75 % yield, respectively (Scheme 1). **PIPER** was also synthesized according to the reported procedure,^[12]^ and used as a positive control. Curiously, the dianhydrides used in **10** and **11** were the only cores where tetra‐substitution was observed. Further investigation of LC‐MS results from the isolated fractions of **11** revealed the presence of tri‐substituted species containing an imide and two amides as a minor product (MH_2_^2+^=479). There was no evidence of tri‐substituted species containing three amides and one carboxylic acid (expected MH_2_^2+^=488). Analysis of LC‐MS data from the isolated fractions of **10** revealed the presence of multiple species with different core substitution patterns, amongst them the tri‐substituted species with an imide and two amides (MH_2_^2+^=380) as well as the di‐substituted bisimide, and the bis(amide/carboxylic acid). Again, no evidence of the tri‐substituted species containing three amides and one carboxylic acid was found (expected MH_2_^2+^=389). The presence of multiple species with a lower degree of substitution is in accordance with the lower yield obtained for compound **10** (40 %) compared to **11** (75 %) and indicates that this reaction proceeded to a lesser degree of completion. These results suggest that tetra‐substitution of **10** and **11** goes through the bisimide intermediate, which is presumably ring‐opened by further nucleophilic addition. The variation in isolated yields can be explained by several factors. Comparing the synthesis of PDI **3** (yield 39 %) with **PIPER** (yield 77 %), it is clear that water is a better solvent than molten imidazole for the synthesis of amphiphilic PDIs. The low yields for compounds **7** (yield 33 %) and **9** (yield 16 %) can be explained by competition between the desired di‐substituted product and undesired tri‐ and tetra‐substituted side products, which are removed during purification. Also in the case of **9**, it would appear that DMSO/NEt_3_ are not favourable reaction conditions for imide condensation. The remaining variation in yield likely results from interplay between the sterics and electronics of the cores, though no obvious trends stand out.

With the library in hand, we began our investigation into G4 stabilization by conducting a Förster resonance energy transfer (FRET) melting assay to assess DNA stabilization using the procedure outlined by De Cian *et. al*.^[13]^ Briefly, compounds **1** and **3**–**11** were tested against four model G4‐forming oligonucleotides and a hairpin duplex sequence to assess G4:Duplex selectivity as well as G4:G4 selectivity. The sequences examined (at 200 nM) were the human telomeric G4 in potassium buffer (F21T‐K^+^, mixed parallel/hybrid G4)^[14]^ and sodium buffer (F21T‐Na^+^, antiparallel G4),^[15]^ the c‐Myc promoter G‐quadruplex (FmycT‐K^+^, parallel G4),^[16]^ a polymorphic G4 found in *T. brucei* (FEBR1T‐K^+^, a mixed G4 topology),^[6b]^ and a hairpin duplex DNA sequence (F10T‐K^+^). Together, these sequences represent targets for therapeutic intervention to treat parasitic infections,^[6b]^ or cancer.^[1b]^ The topology of each sequence was confirmed independently via circular dichroism (CD, Figure S1). Initially, all compounds were tested at 10 μM concentration (Table S1 and Figure S2), with active ligands also being tested at 5 μM (Table 1) and 1 μM (Table S2). Results revealed that only compounds **3**, **9** and **11** were capable of stabilizing G4 structures (Table 1 and Figure S2), although compound **9** only exhibited stabilization at the highest concentration of 10 μM. Whilst PDI based ligands **PIPER** and **3** exhibited large shifts in Δ*T*_m_ for all G4 structures and minimal shifts in Δ*T*_m_ for duplex structures (Figure S3–4), spectral overlap between the PDI core and the FAM/TAMRA FRET pair in the 450–650 nm region prevents definitive conclusions about DNA stabilization from FRET melting experiments. Despite this, a significant difference was observed between the apparent Δ*T*_m_ for G4 sequences and the duplex sequence. Compounds **9** and **11** also displayed some mild stabilization of G4 DNA, with minimal stabilization of the F10T duplex (Figure S5‐6). Tetra‐substituted ligand **11** had superior stabilization over di‐substituted **9**, in agreement with the number of basic residues being a primary driver for G4 stabilization.[^9^, ^10d^, ^17^] Lead compound **1** also exhibited modest stabilization of the parasitic Febr1T‐K^+^ G_4_ (Figure S7). The lack of G4 stabilization of compounds **4**–**8** and **10** can likely be attributed to the increased conformational freedom and reduced hydrophobic aromatic surfaces present in many of the structures. Thus, our data suggests that aromatic π‐π stacking interactions and structural rigidity of the core motif are key driving forces for G‐quadruplex stabilization, and should be considered as important factors alongside the number and location of basic residues when designing optimum G‐quadruplex interactive compounds. More rigid cores not only provide hydrophobic and π‐π stacking interactions with terminal G‐tetrads, but also facilitate the correct orientation of side chains into the G4 grooves, leading to a lower entropic penalty for adopting the optimal conformation compared to more flexible cores. Interestingly, our approach also reveals that flexible cores with enough positively charged groups, such as in compound **11**, can show G4 binding ability with a certain selectivity over duplex DNA.

**Table 1:** DNA stabilization of **PIPER** and **1**, **3**, **9** and **11** assessed via FRET Melting Assay at 5 μM ligand and 200 nM DNA concentration. (Δ*T*_m_ reported to 1 °C using Δ*T*_max_, error is reported as σ). Buffers composition was 10 mM KCl, 90 mM LiCl and 10 mM Li cacodylate for F21T‐K^+^, Febr1T‐K^+^ and F10T‐K^+^; 1 mM KCl, 99 mM LiCl and 10 mM Li cacodylate for FmycT‐K^+^; and 100 mM NaCl and 10 mM Li cacodylate for F21T‐Na^+^. Data for **1** against non‐parasitic G4 s has been previously reported and is reproduced with permission.^[9]^

Compound	F21T‐K^+^	F21T‐Na^+^	FMycT‐K^+^	Febr1T‐K^+^	F10T‐K^+^
**PIPER** ^[a]^	37±2	17±2	21±2	33±1	1±1
**1**	14±2	‐2±1	7±2	9±1	1±1
**3** ^[a]^	34±1	35±4	37±2	33±1	6±2
**9**	−1±1	−4±1	−1±2	−4±1	0±1
**11**	7±2	1±1	5±1	6±1	0±1

[a] Spectral overlap between the ligand and the FAM/TAMRA FRET pair may interfere with these results. Additionally, the oligonucleotide did not completely unfold under these conditions. These results are therefore not reliable indications of DNA Stabilization.

**Figure 2:**
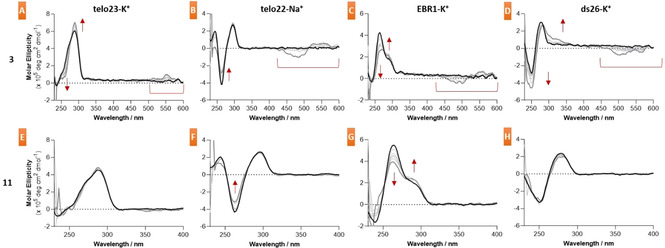
CD titrations of compounds **3** and **11** with telo23‐K^+^, telo22‐Na^+^, EBR1‐K^+^ (G‐quadruplex) and ds26‐K^+^ (duplex) DNA structures, probing the effects of the compounds on DNA topology. (A–D) Effects of **3** on the (A) telo23‐K^+^, (B) telo22‐Na^+^, and (C) EBR1‐K^+^ G‐quadruplex structures, and (D) the ds26‐K^+^ duplex. (E–H) Effects of **11** on the (E) telo23‐K^+^, (F) telo22‐Na^+^, and (G) EBR1‐K^+^ G‐quadruplex structures, and (H) the ds26‐K^+^ duplex. Red arrows denote changes in molar ellipticity observed, whilst the red underlined region denotes induced CD from **3**. Oligonucleotide concentration was 5 μM, with ligand concentration varied up to 10 equivalents (50 μM). For full details, see Supporting Information.

To further probe the G4 and duplex DNA stabilization potential of ligands **1**, **3**, **9**, and **11**, Circular Dichroism (CD) studies were conducted on four different oligonucleotide structures (Figure 2 and S8–9). These were: telo23‐K^+^ (which forms a hybrid G4),^[14]^ telo22‐Na^+^ (which forms an antiparallel G4),^[15]^ EBR1‐K^+^ (which forms a mixed G4)^[6b]^ and ds26‐K^+^ (which forms a self‐complementary duplex). Together, these sequences cover a range of DNA topologies with therapeutic potential. The EBR1‐K^+^ G4 is specific to *T. brucei*, whilst the telomeric G4s (telo23‐K^+^ and telo22‐Na^+^) are common to both human and *T. brucei* genomes. Oligonucleotide concentrations were 5 μM in all cases, whilst ligand concentration was varied from 1 to 10 equivalents. Results from the titration of **1** with the EBR1‐K^+^ G4 and ds26‐K^+^ duplex revealed perturbation of both structures upon binding (Figure S8). This is similar to the G4 disruption previously observed for the perturbation of **1** with telo23‐K^+^ and telo22‐Na^+^G4’s.^[9]^ These results are also in accordance with previous observations of the G4:duplex selectivity of **1**, where a significant reduction in G4 stabilization was observed via FRET melting experiments when competitor duplex DNA was present.^[9]^ Titration of **3** with each oligonucleotide revealed perturbations in the observed CD signal for every structure, and were more significant with the EBR1‐K^+^ G4 and ds26‐K^+^ duplex. It is interesting to note the presence of an induced CD band at 400–600 nm that is either due to induced CD from interaction with the oligonucleotide, or from the ligand itself, as PDIs are known to aggregate into helical structures with CD bands in this region.^[18]^ However a control titration of the ligand into buffer in the absence of DNA revealed no CD signal (data not shown). The induced CD signal can therefore be attributed to the binding of ligand **3** to the chiral DNA structures. Di‐substituted ligand **9** exhibited no effect on any DNA structure examined via CD (Figure S9). In contrast, tetra‐substituted ligand **11** modified the CD signal of the telo22‐Na^+^ G4 and the EBR1‐K^+^ G4 upon binding, showing no change when binding to the telo23‐K^+^ G4 or the ds26‐K^+^ duplex structure.

**Figure 3:**
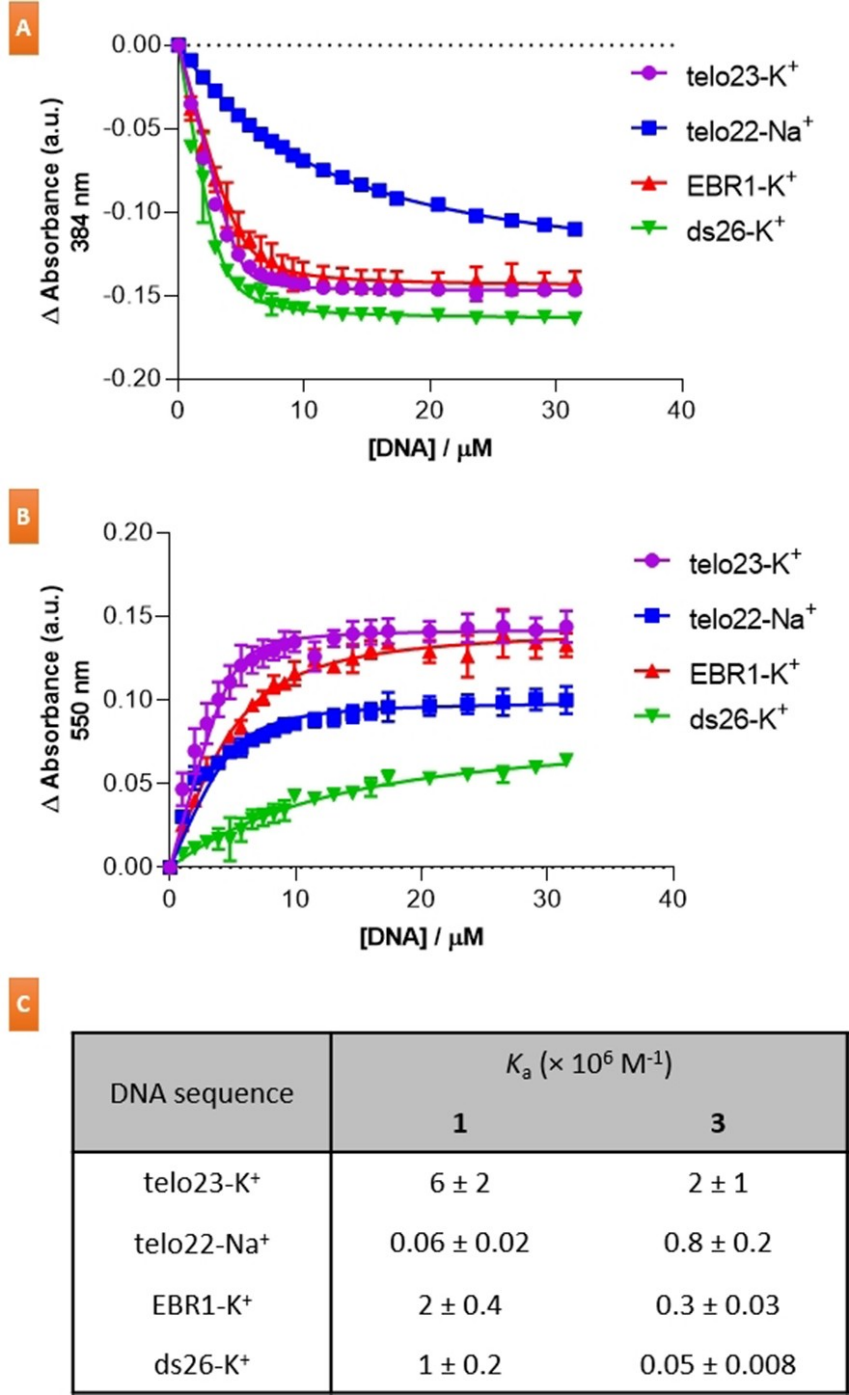
UV/Vis binding isotherms and binding constants (*K*_a_) for the association between compounds **1** (A) and **3** (B) with telo23‐K^+^, telo22‐Na^+^, EBR1‐K^+^, and ds26‐K^+^ DNA structures, following the change in ligand absorbance at 384 nm for **1** (A) or 550 nm for **3** (B). (C) Binding constants determined for the association of **1** and **3** with each of the DNA structures examined, fitted using an independent/equivalent sites binding model, with 2 : 1 ligand:DNA stoichiometry. Ligand concentration was 10 μM, with oligonucleotide concentration varied up to 30 μM. For full details, see Supporting Information.

To obtain quantitative information on the binding of NDI **1** and PDI **3** to the previously assessed G4 and duplex DNA structures, ultraviolet‐visible absorbance spectroscopy (UV‐Vis) titrations were conducted (Figure 3 and S10). In all cases the ligand concentration was kept constant at 10 μM, whilst oligonucleotide was titrated in up to a final concentration of 30 μM. The observed binding isotherms were fitted to an independent/equivalent sites binding model, and binding constants (*K*_a_) were determined. Results from the titration of telo23‐K^+^, telo22‐Na^+^, EBR1‐K^+^ and ds26‐K^+^ oligonucleotides into a solution of NDI **1** (Figure 3 and S10) revealed hypochromicity, and a red‐shift in the absorbance of the ligand, which is indicative of end‐stacking interactions with the G4 sequences. The titration with telo23‐K^+^ and telo22‐Na^+^ yielded a *K*_a_ of 6×10^6^ and 6×10^4^, respectively. The *K*_a_ determined for the binding of **1** to the telo23‐K^+^ G4 via UV‐Vis is in agreement with the *K*_a_ determined previously via isothermal titration calorimetry (ITC).^[9]^ The 100‐fold selectivity observed for the hybrid form of the telomeric G4 over the anti‐parallel form is also in agreement with previous observations from FRET melting assays,^[9]^ providing quantitative information on the unique interactions **1** has with different topologies of the telomeric G4. Examples of G4 ligands with this level of discrimination between different G4 topologies are comparatively rare, and further examination of this apparent selectivity might yield useful insights into the future developments of compounds with interesting G4:G4 discrimination. Compound **1** also exhibited significant binding to the EBR1‐K^+^ G4 and ds26‐K^+^ duplex (*K*_a_=2×10^6^ and 1×10^6^, respectively). These results confirm the significant binding to duplex DNA observed for compound **1**, which is similar to its affinity for the EBR1‐K^+^ G4, and indicate that it is not as selective for G4 structures over duplex structures as FRET measurements suggest. Attempts to fit the observed binding isotherms to a 1 : 1 binding model were unsuccessful, resulting in poor fitted curves, whilst a 2 : 1 model described the data more convincingly. This is in agreement with the 2 : 1 stoichiometry observed via ITC for the telo23‐K^+^ G4 observed previously for compound **1**,^[9]^ and is also in agreement with potential end‐stacking of the ligand on terminal G‐tetrads. Results from the titration of the various oligonucleotides into a solution of PDI **3** revealed a similar affinity for the telo23‐K^+^ G4 (*K*_a_=2×10^6^), but a much better selectivity profile, with 40‐fold selectivity over the ds26‐K^+^ duplex (*K*_a_=5×10^4^) and ∼7‐fold selectivity over the EBR1‐K^+^ G4 (*K*_a_=3×10^5^) versus 6‐fold and 3‐fold, respectively, for **1**.

Unlike NDI **1**, PDI **3** displayed appreciable binding to the telo22‐Na^+^ G4 (*K*_a_=8×10^5^), indicating that the specific selectivity observed for **1** is not applicable to the larger PDI core of **3** (Figure 3 and S10). Like compound **1**, **3** was also best described by a 2 : 1 binding model over a 1 : 1 model. The initial absorbance profile of PDI **3** was consistent with literature reports of aggregated perylene bisimide dyes, and inconsistent with the absorbance spectra of monomeric PDIs, indicating that **3** exists in an aggregated form under these conditions.[^18^, ^19^] Additionally, we observed the presence of a shoulder at 550 nm, which is associated with the presence of face‐to‐face π‐π stacks of the PDI core (*H*‐aggregates)[^18^, ^19a^] and comparable to the charge‐transfer exciton band reported for the perylene analogue perylenetetracarboxylic dianhydride,^[20]^ indicating that the PDI cores are in close proximity to each other. The aggregation of **3** under aqueous conditions was further reinforced by comparing the NMR spectra of **3** in water with that in CDCl_3_ (Figure S11). In CDCl_3_, the spectrum is well resolved, with observable fine structure. In water, significant line‐broadening is observed, with a complete loss in fine structure which is in accordance with aggregated species.^[21]^ Upon addition of DNA, a hyperchromic shift in absorbance is observed, with the absorbance profile of **3** exhibiting well‐resolved vibrionic structure, resembling that of monomeric PDIs.[^18^, ^19^, ^22^] This data demonstrates that PDI **3** undergoes disaggregation in the presence of the DNA sequences examined here, leading to monomeric PDIs that are bound to G4 or duplex DNA. These results imply that the *K*_a_ for the binding of **3** to the G4 and duplex sequences examined here must be higher than the *K*_a_ for the self‐association of **3** which leads to aggregation. Thus, the presence of the G4/duplex sequences examined is sufficient to induce the disaggregation of PDI **3**. This observation is in agreement with previous work by Kern et. al.[^19a^, ^22^] who showed that the binding of PDIs to G4 and duplex DNA is mediated through ligand disaggregation. Unfortunately, the absorbance of compounds **9** and **11** overlapped with the absorbance from the oligonucleotide (λ_abs_ <300 nm), preventing analysis via UV‐Vis titration.

**Figure 4:**
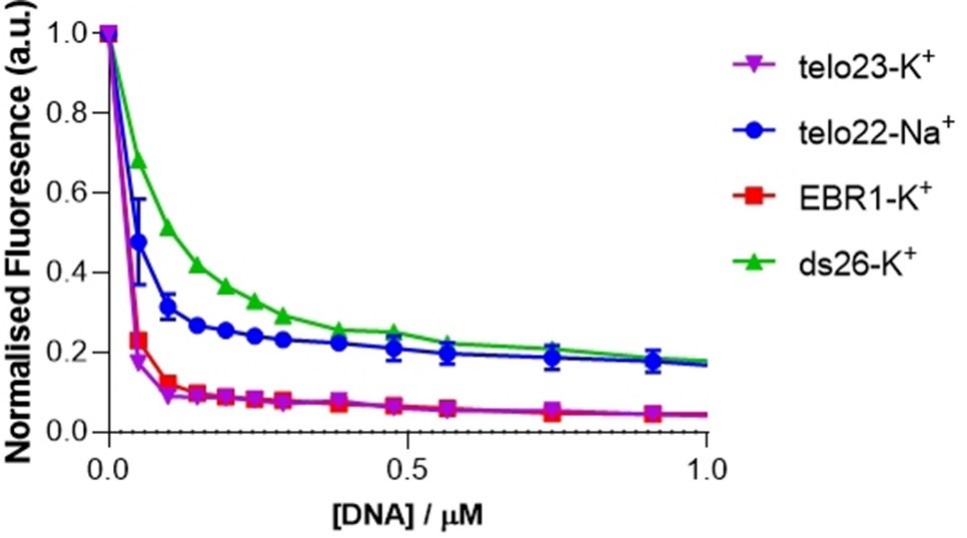
Binding isotherms for association of **3** (1 μM) with up to 1 μM of (A) telo23‐K^+^, (B) telo22‐Na^+^, and (C) EBR1‐K^+^ G‐quadruplex and (D) ds26‐K^+^ DNA, following the change in fluorescence of **3** at 550 nm. For full details, see the Supporting Information.

To further investigate the effects of DNA binding on PDI **3**, fluorescence titrations were conducted on the previously assessed G4 and duplex DNA structures (telo23‐K^+^, telo22‐Na^+^, EBR1‐K^+^ and ds26‐K^+^). The concentration of **3** was kept constant at 1 μM, and the concentration of oligonucleotide was varied up to 1 μM. The results (Figure 4 and S12) reveal that **3** undergoes significant fluorescence quenching in the presence of DNA. Interestingly, a high level of fluorescence quenching was observed at very low levels of DNA, with a ligand:DNA ratio of 5 : 1 for ds26‐K^+^ and between 7 : 1 to 10 : 1 for G4 DNA. This phenomenon has been previously observed for the binding of PDI ligands to G4 and duplex DNA.^[19a]^ The stoichiometry of >5 : 1 is in stark contrast to the stoichiometry observed via UV‐Vis and implies that phenomena other than specific binding to DNA may be responsible for the observed fluorescence quenching. Considering that PDI **3** has been shown to undergo aggregation in aqueous conditions, we hypothesize that the reduction in fluorescence is likely due to the disaggregation phenomena, which may result in increased fluorescence quenching. The sharpest fluorescence quenching was observed for telo23‐K^+^, which is in accordance with UV‐Vis data that suggested **3** has the strongest interaction with this structure. The lowest fluorescence quenching was observed for ds26‐K^+^, which is also in agreement with UV‐Vis data that suggested **3** has the weakest interaction with duplex DNA. Whilst the EBR1‐K^+^ G4 also displayed a similar fluorescence quenching profile to telo23‐K^+^, telo22‐Na^+^ induced a lower level of fluorescence quenching, similar to that of ds26‐K^+^, which is in contrast to the binding observed via UV‐Vis. Overall, fluorescence titrations revealed that **3** undergoes significant fluorescence quenching in the presence of G4 DNA, and a lower degree of fluorescence quenching in the presence of duplex DNA.

**Figure 5:**
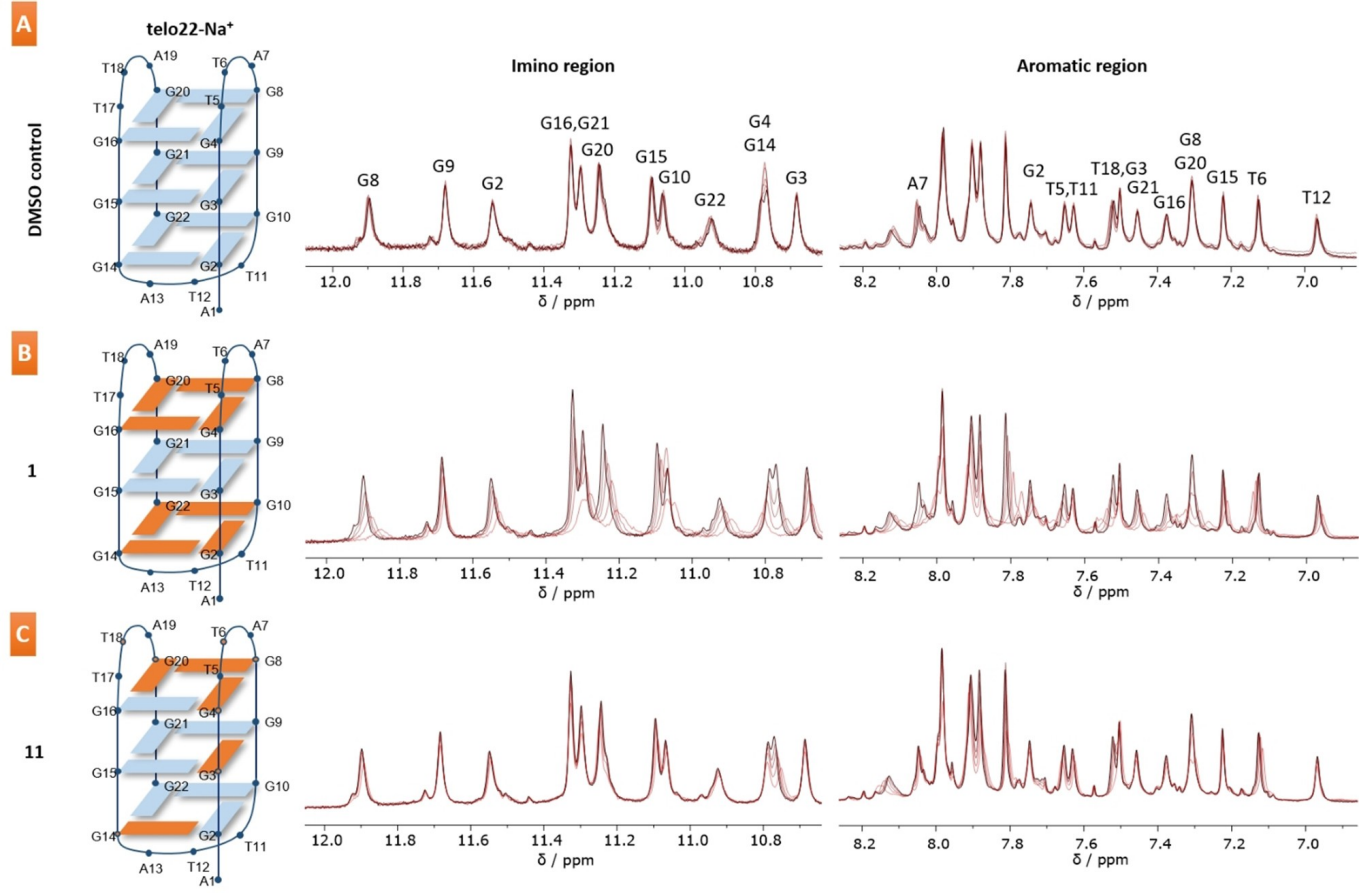
(A) NMR titration of the telo22‐Na^+^ G‐quadruplex with DMSO as control ligand. (B–C) NMR titrations of (B) **1** and (C) **11** with the telo22‐Na^+^ G‐quadruplex. Telo22‐Na^+^ concentration was 185 μM in all cases, with 0 (black trace), 0.25, 0.50, 1, and 2 equivalents of ligand added (red traces). Orange residues in the schematics denote interactions between the ligand and quadruplex. Solutions of DNA were prepared in 25 mM sodium phosphate buffer, pH 7.0 containing 70 mM sodium chloride and 10 % D_2_O. For full details, see Supporting Information.

To further assess how compounds **1**, **3**, **9** and **11** interacted with G4 DNA, NMR titrations were conducted with the telo22‐Na^+^ G4, as it forms a single, well‐defined topology – the antiparallel basket (Figure 5 and S13). The oligonucleotide concentration was kept constant at 185 μM, and 0.25, 0.5, 1, or 2 equivalents of ligand were added. The spectrum for the oligonucleotide in aqueous buffer was indicative of an antiparallel basket (Figure 5A) and addition of DMSO as a control ligand barely changed the NMR signals. Results for the titration with **1** revealed significant perturbations of all protons in the imino and aromatic regions and a minor shift for the G9 imino proton (Figure 5B). This could be consistent with **1** interacting with the terminal G‐tetrads and a stoichiometry of 2 : 1, if, at the same time, it is inducing significant topological changes to the quadruplex upon binding that also affects the internal G4 tetrad. In fact, end‐stacking would mean significantly affecting the lateral and diagonal loops configuration since they may prevent the NDI core motif from efficiently stacking on the terminal G tetrad. These results are also consistent with previous molecular docking studies of **1**, which also suggested an end‐stacking binding mode.^[9]^ Furthermore, this scenario may also provide a plausible explanation for the low binding constant observed for **1** against the telo22‐Na^+^ G4 due to this potential induced‐fit binding mechanism.

In contrast, PDI **3** had no discernible change on the chemical shift of the telo22‐Na^+^ G4 despite the interaction being observable via CD, UV‐Vis, and fluorescence (Figure S13B). This effect has been observed for other G4 ligands such as Neidle's diphenyl triazole ligands when binding to telo22‐Na^+^ or HIVPRO1 G4’s and has been attributed to unspecific binding.^[23]^ In case of compound **3** which shows concentration‐dependant aggregation, the high concentrations used in the NMR titration could alter the equilibrium between the PDI aggregates and binding to G4 DNA, favouring PDI aggregation and a weak association with the telo22‐Na^+^ G4. Similarly, di‐substituted **9** also exhibited minimal effects on the telo22‐Na^+^ G4 (Figure S13C), which is in accordance with the minimal topological change upon binding revealed by the CD titrations, and also points to potential unspecific binding. In contrast, tetra‐substituted **11** exhibited small specific perturbations of imino protons G4 and G14 as well as aromatic protons both in the tetrads (G3, G8 and G20) and in the loops (T5, T6, T11 and T18, Figure 5C). This indicates binding interactions predominantly located at the upper tetrad and on one of the grooves, with a clear different mode to that observed for compounds **1**, **3** and **9**, possibly due to its tetra‐substitution with four positive charged groups in an octopus‐like arrangement.

To investigate the potential anticancer and antiparasitic activity of compounds **1** and **3**–**11**, cytotoxicity assays were conducted on MRC5 non‐tumoral cells and on HeLa cervical carcinoma cells, as well as *T. brucei* and *L. major* parasites. Doxorubicin, suramin and miltefosine were used as control compounds.[^6b^, ^9^] After incubation with varied concentrations of the compounds for 72 h, cell viability was measured using either an alamarBlue™ assay (for MRC5, HeLa and *T. brucei*) or MTT assay (for *L. major*). Absolute EC_50_ values and selectivity indexes were then calculated from dose‐response curves (Table 2 and S14). The results revealed that G4 interactive compounds **1**, **3**, **9**, and **11** all inhibited cell viability, with the highest selectivity index for HeLa cells over MRC5 cells obtained by compound **1** at 10‐fold. The most active compounds against parasites were **1**, **3** and **11**, which also exhibited the highest G4 stabilization. All three were particularly effective against *T. brucei*, with **3** exhibiting the best EC_50_ value in the nanomolar range. Compounds **3** and **11** showed the highest selectivity for *T. brucei* over MRC5, with **3** displaying a 78‐fold selectivity. This represents a large potential therapeutic window, since considerably lower toxicity is observed towards non‐tumoral MRC5 cells (EC_50_=2 μM). The efficacy and selectivity of **1**, **3** and **11** for *T. brucei* over MRC5 was higher than for other typical G4 interactive compounds such as Pyridostatin and BRACO‐19 (EC_50_ values of 5.5 and 7.8 μM, and selectivity index (SI) of 0.7 and 1.5, respectively).^[6b]^ In the case of the activity towards *L. major*, only compounds **1** and **11** displayed relevant toxicity (EC_50_ values of 0.47 and 18 μM, respectively). Whilst no correlation between G4 stabilization and anticancer activity was observed for these compounds, cytotoxicity towards *T. brucei* was correlated with G4 stabilization, with **1**, **3** and **11** all exhibiting antiparasitic activity. Certainly, other mechanisms of action must also be considered and will be studied in the future.

**Table 2:** Cellular cytotoxicity and antiparasitic activity after 72 h incubation with **1** and **3**–**11** as well as **Doxorubicin** (MRC5/HeLa positive control), **Suramin** (*T. brucei* positive control) and **Miltefosine** (*L. major* positive control). Reported as absolute EC_50_ values measured in μM using alamarBlue™ fluorescence (for MRC5, HeLa and *T. brucei*) or MTT absorbance (for *L. major*) to assess cellular metabolism, with error represented as σ. SI = selectivity index. The best results are highlighted in **bold**. Some data for **1** and **Doxorubicin** have been previously reported.^[9]^

Compound	MRC5	HeLa	*T. brucei*	*L. major*	SI (MRC5/HeLa)	SI (MRC5/*T. brucei*)	SI (MRC5/ *L. major*)
Doxorubicin	–	0.40±0.05		–			–
Suramin	–	–	0.044±0.006		–		–
Miltefosine		–	–	5.8±0.01	–	–	
**1**	**3.9±0.5**	**0.5±0.05**	**0.61±0.3**	**0.47±0.07**	**9.5**	**6.4**	**8.3**
**3**	**2.1±0.6**	**53±6.2**	**0.027±0.01**	**>100**	**0.04**	**78**	**–**
**4**	>100	>100	57±10	>100	–	–	–
**5**	>100	>100	67±18	>100	–	–	–
**6**	>100	>100	36±2	>100	–	–	–
**7**	>100	>100	70±13	>100	–	–	–
**8**	>100	>100	52±7	>100	–	–	–
**9**	88±10	29±3.5	33±24	>100	3.0	2.7	–
**10**	>100	>100	55±3	>100	–	–	–
**11**	**59±37**	**14±1**	**2.8±2**	**18±1**	**4.2**	**21**	**3.2**

**Figure 6:**
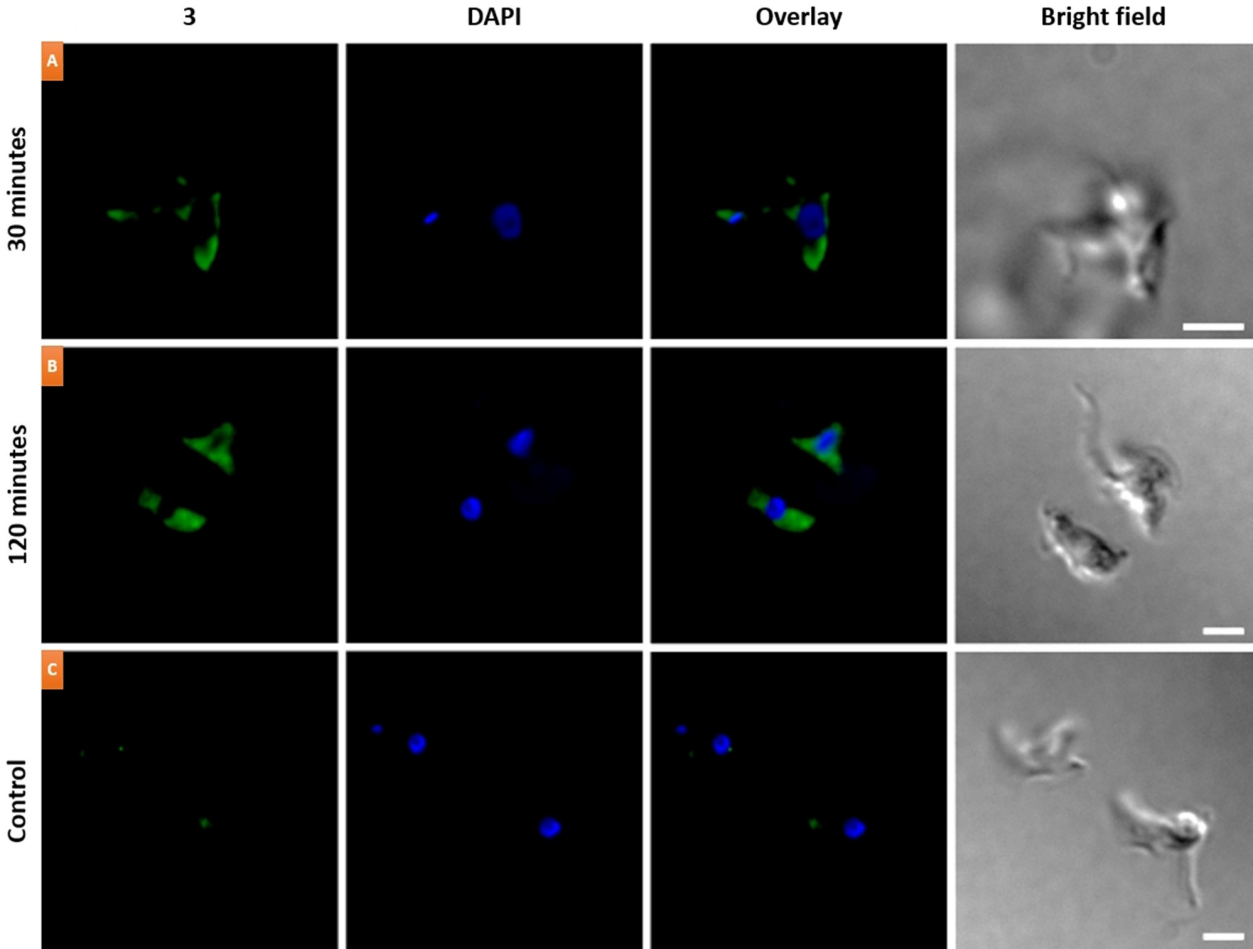
Fluorescence microscopy images of *T. brucei* parasites after incubation with **3** (5 μM) for (A) 30 min or (B) 120 min respectively. Control parasites are displayed in (C). Nuclear staining was performed using DAPI (blue fluorescence). Excitation was performed with the 350–450 and 492–518 filters for DAPI and **3**, respectively. A triple filter (437–474, 508–550 and 595–670 nm) was used to detect the fluorescence emission of both DAPI and **3**. Scale bar=5 μm.

The inherent fluorescence of PDI **3** enabled us to track its localization in both cells and parasites. Preliminary absorbance and fluorescence experiments indicated that **3** exists in *H‐*aggregated form in PBS at 25 μM (Figure S15). The ability of PDI **3** to undergo internalization into both HeLa (human cervical carcinoma) cells and *T. brucei* parasites was then assessed via fluorescence microscopy (Figure 6 and Figure S16). After incubating HeLa cells with **3** (5 μM) for 30 minutes, limited uptake was observed (Figure S16A), however greater uptake was detected after 120 minutes (Figure S16B). The lack of colocalized fluorescence with DAPI staining suggested limited presence of compound **3** in the nucleus, although it can be observed in the rest of the cell. A similar scenario occurred for **3** when assayed in *T. brucei*. Uptake of **3** was observed after just 30 minutes incubation (Figure 6A), and remained after 120 minutes (Figure 6B). DAPI staining of the nucleus and kinetoplast also revealed no overlap with the fluorescence emission of **3**. In both cases, the lack of fluorescence emission from **3** in the nucleus or kinetoplast can be expected given that **3** exhibits significant fluorescence quenching in the presence of small quantities of DNA. In any case, further experiments would be needed to rule out the possibility that **3** is unable to localize into the nucleus and the kinetoplast.

## Conclusion

In conclusion, we have developed a practical strategy for the preparation of a diverse array of amphiphilic compounds using simple yet versatile imide coupling chemistry, examining their potential use as G4 ligands and as therapeutic agents. A library of nine compounds with different core motifs were produced in one step, from cheap, commercially available starting materials. We have examined the role of the core motif in facilitating G4 binding, highlighting the importance of core rigidity such as in the case of compounds **1** and **3**. Most compounds with a flexible or non‐fused core did not induce G4 stabilization. However, it is interesting to note that compound **11**, containing a flexible core and four positively charged groups arranged in an octopus‐like pose, is capable of G4 binding thus opening a potential alternative strategy for developing G4 binders. In total, four compounds showed binding to G4 DNA using FRET and their interactions with a variety of G4 and duplex DNA structures was assessed via CD, UV‐Vis, Fluorescence, and NMR titrations. The results revealed that previous NDI **1** exhibits appreciable binding to duplex DNA, though **1** also displayed 100‐fold selectivity for the mixed parallel/hybrid telo23‐K^+^ G4 over the antiparallel telo22‐Na^+^ G4. Compound **1** also seems to interact with the telo22‐Na^+^ G4 through end‐stacking, which corroborates the 2 : 1 binding stoichiometry previously determined. The analogous PDI, **3**, exhibited significant binding to G4 DNA, and superior selectivity for G4 DNA over duplex DNA, with a 40‐fold selectivity for the telo23‐K^+^ G4 over the ds26‐K^+^ duplex. The core‐diversification yielded a new linear, flexible core with four non‐fused aromatic rings and a much greater degree of conformational freedom, that marks a departure from the fused polycyclic aromatic NDI core on which it was based. Di‐substitution of this core (compound **9**) was insufficient to induce DNA stabilization, however the tetra‐substituted analogue **11** exhibited selective stabilization of G4 DNA over duplex DNA, and interacted with specific residues at the top tetrad and on one of the groves of the telo22‐Na^+^ G4. The anticancer and antiparasitic activity of the library was also examined, with PDI **3** displaying potent toxicity towards *T. brucei*, and 78‐fold selectivity over control MRC5 cells. The potent antiparasitic activity of **3** correlated with strong stabilization of G4 DNA, although other modes of action could be operating. Compound **3** was internalized into both HeLa cells and *T. brucei*, and can be imaged at therapeutically relevant concentrations (5 μM) with uptake that is time dependent. No evidence for nuclear or kinetoplast uptake of **3** was found, which could be due to the fluorescence quenching of **3** in the presence of DNA. We hope this approach to diversify the core of future G4 ligands may inspire other library‐based convenient approaches to G4 interactive compounds that could help identify potent and selective G4 ligands with promising therapeutic activity.

## Supporting Information

Experimental Details, including further FRET melting experiments, CD experiments, UV‐Vis experiments, fluorescence experiments, NMR experiments, cytotoxicity studies and fluorescence microscopy is available in the supporting information.

## Conflict of interest

The authors declare no conflict of interest.

## Supporting information

As a service to our authors and readers, this journal provides supporting information supplied by the authors. Such materials are peer reviewed and may be re‐organized for online delivery, but are not copy‐edited or typeset. Technical support issues arising from supporting information (other than missing files) should be addressed to the authors.

Supplementary
