# Supplementary material for: Imide Condensation as a Strategy for the Synthesis of Core‐Diversified G‐Quadruplex Ligands with Anticancer and Antiparasitic Activity
Source: Chemistry. 2021 May 2;27(28):7712–21. doi: 10.1002/chem.202100040 (PMC8251916; doi:10.1002/chem.202100040)
Supplement: Supplementary file 1 — Supplementary [file CHEM-27-7712-s001.pdf]

Chemistry–A European Journal

Supporting Information

Imide Condensation as a Strategy for the Synthesis of Core-Diversified G-Quadruplex Ligands with Anticancer and Antiparasitic Activity**

Steven T. G. Street, Pablo Peñalver, Michael P. O'Hagan, Gregory J. Hollingworth, Juan C. Morales,* and M. Carmen Galan*

Table of Contents

Experimental Details	3
General Experimental Details	3
NMR Spectroscopy	4
Mass Spectrometry	4
Infrared Spectroscopy	4
Chromatography	5
FRET Melting Assays	5
Circular Dichroism	7
UV-Vis Absorbance Spectroscopy	7
Fluorescence Spectroscopy	8
NMR Titration experiments	9
Cell Culture Protocols	9
Toxicity assays	10
Fluorescence Microscopy	11
Synthetic Experimental Procedures and Data	13
Supplementary Figures & Tables	23
NMR Spectra of Novel Compounds	38
References	53

Experimental Details

General Experimental Details

Chemicals were purchased from Science Warehouse and used without further purification. All oligonucleotides used were purchased from Eurogentec (Belgium), purified by HPLC and delivered dry. Oligonucleotide concentrations were determined by UV-absorbance using a NanoDrop 2000 Spectrophotometer from Thermo Scientific. All oligonucleotides were annealed prior to use by heating for 2 minutes at 90 °C and then placed immediately into ice. Dry solvents were obtained by distillation using standard procedures, or by passage through a column of anhydrous alumina using equipment from Anhydrous Engineering (University of Bristol) based on the Grubbs' design.[S1] Reactions requiring anhydrous conditions were performed under N_2; glassware and needles were either flame dried immediately prior to use, or placed in an oven (150 °C) for at least 2 h and allowed to cool under reduced pressure. Liquid reagents, solutions or solvents were added via syringe through rubber septa; solid reagents were added via Schlenk type adapters. Reactions were monitored by TLC on Kieselgel 60 F_{254} (Merck). Aromatic compounds were detected with UV light (254 or 365 nm), and amines were detected by staining with ninhydrin. Flash column chromatography was performed according to Still and co-workers,[S2] using silica gel [Merck, 230−400 mesh (40−63 µm)]. The crude material was applied to the column by pre-adsorption onto silica, as appropriate. Reverse phase flash chromatography was performed on a Teledyne Isco Combiflash purification system, with RediSep Rf Gold C18 cartridges. Solvents for flash column chromatography (FCC) and thin layer chromatography (TLC) are listed in volume:volume percentages. Extracts were concentrated in vacuo using both a Heidolph Hei-VAP Advantage rotary evaporator (bath temperatures up to 50 °C) at a pressure of 15 mmHg (diaphragm pump) or 0.1 mmHg (oil pump) as appropriate, and a high vacuum line at room temperature. Water soluble compounds were freeze dried on a Lyotrap Plus (LTE Scientific LTD).

NMR Spectroscopy

^{1}H-NMR, ^{19}F-NMR and ^{13}C-NMR spectra were measured at 25 °C in the solvent specified with Varian or Bruker spectrometers (some equipped with a cryoprobe), operating at the field strengths listed. Chemical shifts are quoted in parts per million with spectra referenced to the residual solvent peak. Multiplicities are abbreviated as: br (broad), s (singlet), d (doublet), t (triplet), q (quartet), p (pentet), m (multiplet) and *app.* (apparent) or combinations thereof. Assignments of ^{1}H-NMR and ^{13}C-NMR signals were made where possible, using COSY, HSQC and HMBC experiments. ^{19}F-NMR spectra were recorded with 2-fluorobenzoic acid present as an internal standard to quantify the amount of trifluoracetate (TFA) counterion. For TFA quantification, the integration of the internal standard was compared with TFA in ^{19}F-NMR and the cation in ^{1}H-NMR and the ratio between the two taken.

Mass Spectrometry

High-resolution mass spectra were obtained by the University of Bristol mass spectrometry service by electrospray ionisation (ESI) or matrix assisted laser desorption ionisation (MALDI) modes. Reactions followed by MALDI were analysed using a Bruker Ultraflextreme time-of-flight/time-of-flight mass spectrometer running in reflector mode. Samples were prepared using 2,5-dihydroxybenzoic acid as the matrix.

Infrared Spectroscopy

Infrared spectra were recorded in the range 4000 - 650 cm^{-1} on a Perkin Elmer Spectrum Two Spectrometer either as neat films or solids compressed onto a diamond window.

Chromatography

Analytical high-performance liquid chromatography (HPLC) was performed on a Waters system with a Waters 2707 Autosampler, a Waters 2535 Quaternary Gradient Module, a Waters In-Line Degasser, a Waters Temperature Control Module II, a Waters 2424 ELS Detector and a Waters 2998 Photodiode Array Detector (PDA) set to monitor 210 – 450 nm. Compound purity was assessed by monitoring the PDA at the wavelength specified (λ_{max} of the aromatic core), using the conditions as follows: The analytical column was a Phenomenex Luna 3 µm C18(2) 100 Å (250 × 4.6 mm). For the analytical method, the flow rate was 0.75 mL / min and the mobile phases used were 0.05 % formic acid in water for the aqueous phase and 0.05 % formic acid in methanol for the organic phase. The gradient was 5 % organic phase for 5 min at the start before increasing to 40 % organic phase over 25 min, then increasing to 95 % organic phase over 1 min before holding at 95 % for 9 min.

Analytical liquid chromatography-mass spectrometry (LC-MS) was performed on a Waters Acquity Ultra Performance LC-MS with PDA detector, set to monitor 100 - 1600 m/z on the MS and 210 – 450 nm on the PDA. The column used was a Waters Acquity UPLC HSS T3 1.8 µm (2.1 × 50 mm). For the method, the flow rate was 1 mL / min, the mobile phases used were 0.05 % formic acid and 0.05 % ammonium acetate in water for the aqueous phase and 0.04 % formic acid in acetonitrile for the organic phase. The gradient was from 5 % organic phase at the start to 98 % organic phase over 1.4 min, then holding at 98 % organic for 0.3 min before returning to 5 % organic over 0.1 min and holding at 5 % organic for 0.2 min.

FRET Melting Assays

FRET melting assays were performed to assess ligand affinity for duplex and G-quadruplex DNA. Briefly, oligonucleotides of interest were obtained labelled at the 5' and 3' ends with FAM (a fluorescence donor) and TAMRA (a fluorescence quencher), respectively. In the folded state, proximity of the donor and quencher result in no observed fluorescence from FAM, since energy is transferred non-radiatively to TAMRA by FRET. As the temperature is raised and the secondary structure denatures, the fluorophores move further apart and the fluorescence signal increases. From the resulting curve, the characteristic melting temperature (T_{max}, also referred to as T_m) is defined as the temperature which corresponds to the maxima of the first derivative of the normalised fluorescence signal. The change in melting temperature (ΔT_m) induced by the presence of a small molecule ligand provides an indication of the ligand's ability to stabilise the DNA structure. FRET experiments were performed according to the

procedure reported by De Cian and co-workers[S3] on a Stratagene MX3005P. The method consisted of holding at 25 °C for 5 min, before heating at 1 °C/min to 96 °C in 1 °C increments, followed by monitoring the fluorescence output at each increment for 1 min. The fluorescence emission of FAM was followed at 516 nm, with a 10 nm full width at half-maximum filter and an 8-fold gain, after excitation at 492 nm with a 9 nm full width at half-maximum filter. The oligonucleotides used were as follows:

Quadruplex Model	Sequence
F21T (Human Telomeric G-quadruplex)[S3,S4]	5′-FAM-GGG-TTA-GGG-TTA-GGG-TTA-GGG-TAMRA-3′
FMycT (Predominant G-quadruplex formed in c-Myc Pu27 promoter)[S5]	5′-FAM-TTG-AGG-GTG-GGT-AGG-GTG-GGT-AA-TAMRA-3′
Febr1T (*T.brucei* G-quadruplex)[S6]	5’-FAM-GGGCAGGGGGTGATGGGGAGGAGCCAGGG-TAMRA-3’
F10T (Duplex DNA)[S7]	5′-FAM-TAT-AGC-TAT-A-*HEG*-TAT-AGC-TAT-A-TAMRA-3′

Where FAM = 6-carboxyfluorescein, TAMRA = 6-carboxy-tetramethylrhodamine and HEG = [(-CH_2CH_2O-)$_6$]

The final concentration of oligonucleotide was 200 nM in all cases. The buffer used depended on the sequence in question, and follows that used by the Mergny group.[S3] For F21T in Na^+ conditions, the final buffer concentration was 100 mM NaCl, and 10 mM Li Cacodylate. For FMycT (K^+ Conditions), 1 mM KCl, 99 mM LiCl and 10 mM Li Cacodylate was used. For F21T in K^+ conditions as well as for Febr1T and F10T, 10 mM KCl, 90 mM LiCl and 10 mM Li Cacodylate was used. Ligand concentrations were 1-10 μM. Each sample was tested in duplicate, and each experiment was tested in at least triplicate to assess the reproducibility of all results. Appropriate control experiments were also carried out for each sample set, using the ligand TmPyP4 as a positive control (1 μM). PIPER, Pyridostatin, and Doxorubicin were also tested as positive controls. Data processing was carried out using Origin 9, with ΔT_{max} used to represent ΔT_m.

Circular Dichroism

Circular Dichroism (CD) titrations were recorded using a Jasco J-810 spectrometer fitted with a Peltier temperature controller. Measurements were taken in a quartz cuvette with a path length of 5 mm, at 20°C, at a 500 nm / min scanning speed at 2 nm intervals, with a 1 nm bandwidth. The CD spectra were recorded between 400 and 230 nm for compounds **1**, **9** and **11** and 600 and 230 nm for compound **3**. Spectra were baseline corrected for the buffer used. The oligonucleotide sequences used were:

Oligonucleotide	Sequence
telo23-K^+ (hybrid type human telomeric G4)[S8]	5'-TAG-GGT-TAG-GGT-TAG-GGT-TAG-GG-3'
telo22-Na^+ (antiparallel basket human telomeric G4)[S9]	5'-AGG-GTT-AGG-GTT-AGG-GTT-AGG-G-3'
EBR1-K^+ (mixed G4 from T.brucei)[S6]	5’-GGG-CAG-GGG-GTG-ATG-GGG-AGG-AGC-CAG-GG-3’
ds26-K^+ (self-complementary duplex)[S10]	5’-CAA-TCG-GAT-CGA-ATT-CGA-TCC-GAT-TG-3’

telo23 and telo22 were selected as models for the human telomeric G-quadruplex under K^+ and Na^+ conditions, respectively, as they have been the subject of detailed structural studies,[S8,S9] and have been shown to form well characterized G-quadruplex topologies under the conditions used. telo23 forms primarily a (3+1) hybrid structure under K+ conditions,[S8] whilst telo22 forms a single antiparallel fold.[S9]

The oligonucleotides were at a concentration of 5 μM and the buffers used were either potassium phosphate (100 mM, pH 7.4) for telo23-K^+, EBR1-K^+ and ds26-K^+ or sodium phosphate (100 mM, pH 7.4) for telo22-Na^+. The reported spectrum for each sample represents the average of 3 scans and is baseline corrected for the buffer used. Data processing was carried out using Prism 7 (GraphPad Software) with a 4-point second order smoothing polynomial applied to all spectra. Observed ellipticities were converted to molar ellipticity.

UV-Visible Absorbance Spectroscopy

Absorbance spectra were recorded on a Cary 60 UV-Visible (UV-Vis) spectrophotometer using a room-light immune fibre optic probe with 10 mm path length. The UV-Vis spectra were recorded between 500 – 300 nm (for compound **1**) and 650 – 300 nm (for compound **3**). Spectra were corrected for background and buffer absorbance. The oligonucleotides and buffer conditions were the same as employed in the CD experiments.

In the titrations, the concentration of ligand was fixed at 10 μM in an initial volume of 500 μL. Aliquots of oligonucleotide were added using a microsyringe from a 100 μM stock solution in appropriate buffer containing also 10 μM ligand to maintain constant ligand concentration. Note: the oligonucleotide solution was annealed by heating to 90 °C for 2 minutes and then cooling on ice prior to the addition of ligand (to avoid annealing in the presence of ligand). Following addition, the solution was mixed thoroughly and the UV-visible spectrum was acquired immediately. Data were fitted to an independent-and-equivalent-sites binding model (Equation 1) using Prism 7 software, a full derivation of which is provided by (amongst others) Thordarson,[S11] adapted to an independent and equivalent sites model by (amongst others) Buurma and Gade.[S12] The stoichiometry of the complex (*N*) was set to 2 (ligand:G4) in order to afford satisfactory fits for the resulting isotherms. The data presented in Figure 3 shows the average values obtained from two independent experiments.

Equation 1:

$$\Delta A = \varepsilon_{\Delta complex}([\text{complex}])$$

$$where:$$

$$[\text{complex}] = \frac{1 + N \cdot K_\text{a} \cdot [\text{DNA}]_{tot} + K_\text{a} \cdot [\text{ligand}]_{tot} - \sqrt{(1 + N \cdot K_\text{a} \cdot [\text{DNA}]_{tot} + K_\text{a} \cdot [\text{ligand}]_{tot})^2 - 4 \cdot N \cdot {K_\text{a}}^2 \cdot [\text{DNA}]_{tot} \cdot [\text{ligand}]_{tot}}}{2 \cdot K_\text{a}}$$

$\Delta A = absorbance\ change\ at\ each\ titration\ point\ relative\ to\ free\ ligand\ (observed\ parameter)$

$\varepsilon_{\Delta complex} = change\ in\ the\ molar\ extinction\ coefficient\ between\ free\ ligand\ and\ DNA\ /ligand\ complex\ (fitted\ parameter)$

$N = the\ binding\ stoichiometery\ of\ ligand\ to\ DNA\ (selected\ parameter)$

$K_\text{a} = the\ apparent\ association\ constant$ (fitted parameter), $K_\text{d} = \frac{1}{K_\text{a}}$

$[\text{DNA}]_{tot} = the\ concentration\ of\ added\ DNA\ (known\ parameter)$

$[\text{ligand}]_{tot} = the\ total\ ligand\ concentration\ (fixed\ parameter)$

Fluorescence Spectroscopy

For Figure 4 (Fluorescence titrations):

Steady-state fluorescence measurements were performed on a Perkin-Elmer spectrofluorometer, in a 10 × 10 mm quartz fluorescence cuvette (Thorlabs). In the titrations, the concentration of ligand was fixed at 1 μM in an initial volume of 2000 μL. Aliquots of oligonucleotide were added using a micropipette from a 10 μM stock solution in appropriate buffer containing also 1 μM ligand to maintain constant ligand concentration. The oligonucleotides and buffer conditions were the same as employed in the CD experiments. The oligonucleotide/ligand solution was prepared in a similar fashion to the UV experiments. Following addition, the solution was mixed thoroughly and the emission spectrum was acquired immediately. Emission spectra were obtained following excitation at 500 nm, and the emission was collected between 520 – 670 nm. A bandwidth of 2 nm was used for the excitation and emission monochromators, and data was collected every 1 nm and normalized to the emission in the absence of ligand using GraphPad Prism 7 Software. The data presented in Figure 4 shows the average values obtained from two independent experiments.

For Figure S13:

Steady-state fluorescence measurements were performed on a Jasco FP-6500 spectrofluorometer, in a 10 × 10 mm quartz fluorescence cuvette (Hellma Analytics). The volume of sample used was 2 mL. Emission spectra were obtained following excitation at 488 nm, and the emission was collected between 500-900 nm. A bandwidth of 2 nm was used for the excitation and emission monochromators, and data was collected every 0.5 nm.

^{1}H-NMR Titrations experiments

All spectra were recorded at 298 K on a 600 MHz Varian VNMRS spectrometer equipped with a triple resonance cryogenically cooled probe head. The oligonucleotide used was telo22; the same as employed in the CD experiments. The DNA concentration was 185 μM in 25 mM sodium phosphate buffer, pH 7.0, containing 70 mM sodium chloride and 10% D_2O. All experiments employed sculpted excitation water suppression. Samples were referenced to the triethylammonium counterion, δ CH_3 = 1.297. During the titrations, aliquots of ligand were added from a 10 mM stock solution in DMSO-d_6 to give the required titration points. A control titration was performed in which equivalent volumes of DMSO-d_6 vehicle (containing no ligand) were added to telo22-Na^+. After addition of ligand aliquots, the sample was mixed thoroughly and the NMR spectrum acquired immediately. The imino and aromatic resonances of telo22-Na^+ in the absence of ligand were assigned where possible by comparison with previously published data.[S9] All spectra were processed with MestReNova software.

Cell Culture Protocols

Cell culture reagents were purchased from Thermo Fisher Scientific (Waltham, MA, USA) and used as-is. The Dulbecco's Minimal Essential Medium (DMEM) formulation contained either high glucose (4.5 g/L, catalogue number: 10569) or low glucose (1 g/L, catalogue number: 10567). RPMI-1640 was provided without sodium pyruvate and with L-glutamine (catalogue number: 11875). Phosphate Buffered Saline (PBS) contained NaCl (9 g/L), KH_2PO_4 (144 mg/L) and Na_2HPO_4-7.H2O (795 mg/L, catalogue number: 10010049). TrypLE Express™ was provided with EDTA (458 mg/L) and without Phenol Red (catalogue number: 12604021).

MRC-5 cells (human lung fibroblasts) were grown in monolayer (37 °C, 5 % CO_2 and 100 % humidity) in DMEM medium (1 g/L glucose), supplemented with 10% heat-inactivated Fetal Bovine Serum (hiFBS), 2 mM L-glutamine, 100 U / mL penicillin and 100 mg/mL streptomycin. HeLa (Human cervical carcinoma cell line) cells were maintained at 37 º C and 5 % CO_2 in high glucose DMEM (4.5 g / L) supplemented with 10% hiFBS, 100 U / ml penicillin, 100 mg / ml streptomycin, 2 mM L-glutamine and non-essential aminoacids (1X). Cells were cultured according to ATCC recommendations and were used for the experiments while in the exponential growth phase.

T. brucei (bloodstream forms, "single marker" S427 (S16) were cultured at 37 ºC, 5 % CO_2 in HMI-9 medium supplemented with 10% heat-inactivated fetal bovine serum (hiFBS,

Invitrogen). *L. major* promastigotes (MHOM/IL/80/Friedlin) were cultured at 28º C, 5 % CO_2 in modified RPMI-1640 medium (Thermo Fisher Scientific, Waltham, MA, USA) with 10% hiFBS. Parasites were split every other day and maintained in their experimental growth phase (below 2 million parasites per mL).

Toxicity Assays

Cytotoxicity

Cytotoxicity was measured through the alamarBlue® assay (Thermo Fisher Scientific).[S6,S13] Briefly, 5×10^3 MRC-5 or HeLa cells were seeded in 96-wells plates (100 µL / well) in the presence of increasing concentrations of compound. After 72 hrs of incubation at 37 ºC, 20 µL of alamarBlue® solution (110 ng / ml) was added to each well and cells were reincubated for 4 hrs at 37 °C. Then, 50 µL of 3 % SDS were added to each well. The plate was incubated at 37 °C for an extra hour and then analysed by fluorescence with an Infinite F200 plate reader (TECAN Austria, GmbH). The excitation wavelength was fixed at 550 nm and the emission wavelength at 590 nm. The results are expressed as the concentration of compound that reduces cell growth by 50 % versus untreated control cells (EC_{50}) using GraphPad Prism 7 (GraphPad Software) to fit the data to a sigmoidal curve. Data are presented as the average of three independent measurements all conducted in triplicate conditions.

Anti-parasitic activity

The trypanocidal activity of the compounds was assessed by the alamarBlue® assay as well. Briefly, 1×10^3 BSF *T. brucei* were incubated in 96-wells plates alone or in the presence of increasing concentrations of compounds for 72 hrs (5 % CO_2, 37 °C) and processed as described above. Data are presented as the average of three independent measurements, all conducted in triplicate conditions.

The leishmaniacidal activity of the compounds on promastigotes *L. major* (MHOM/IL/80/Friedlin) was carried out as previously described.[S14] Briefly, 4×10^6 / mL promastigotes were incubated for 72 h at 28 ºC in 96-well plates (50 µL/well) in the presence of increasing concentration of compounds. Cell proliferation was determined using an MTT-based assay (Sigma-Aldrich). Thus, after the 72 h incubation, 10 µL of MTT (5 mg/ml) were added to each well and parasites were reincubated for 4 hrs at 28 °C. Then, 50 µL of 20 % SDS were added to each well. The plate was then incubated at 37°C for 4-16 hrs and analysed using an Infinite F200 plate reader (TECAN Austria, GmbH). The absorbance was measured

at a wavelength of 540 nm and the EC_{50} was calculated as described above. Data are presented as the average of three independent measurements, all conducted in triplicate conditions.

Fluorescence Microscopy

HeLa cells (2×10^4 cells / mL) and *T. brucei* parasites (1×10^8 / mL) were incubated with **3** (5 µM), in 0.5 mL of each respective medium (without FBS) for 30 and 120 min at 37 °C and 100% humidity.

The parasitic samples were then centrifuged, the supernatant was removed, and parasites were fixed with 200 µL of cold paraformaldehyde (4%) for 30 mins in an ice bath. Then, samples were washed twice with 500 µL cold phosphate buffered saline (PBS), the supernatant was removed, and prolong DAPI staining (10 µL) was added. 15 minutes later 3 µL of the sample was loaded to a poly lysine carrier and processed by microscope observation.

Cells were grown on a cover slide in a 24 well culture plate for 24h and then treated with compound **3**. After that covers were washed 5 times (inside the wells) with 500 µL room temperature PBS and fixed in an ice bath with paraformaldehyde (2%) for 20 minutes. Two extra PBS washings (10 mins each) were also necessary. Then, the cover slide was immersed first in water and lastly in ethanol and left to dry over a whattman filter paper. In the final step Prolong DAPI (3-4 µL) was used as mounting medium and samples were processed by microscope observation.

Images were acquired using a widefield Olympus ix81 microscope. Excitation was performed with the 350-450 and 492-518 filters for DAPI and **3**, respectively. A triple filter (437-474, 508-550 and 595-670 nm) was used to detect the fluorescence emission of both DAPI and **3**.

The images were deconvoluted using Huygens Professional image processing software from Scientific Volume Imaging (http://www.svi.nl). The merge of the images was made with Fiji software (https://fiji.sc/).

Synthetic Experimental Procedures and Data

Bis-*N,N′*-(2-(1-Piperidino)ethyl)-3,4,9,10-perylenetetracarboxylic Diimide (PIPER)

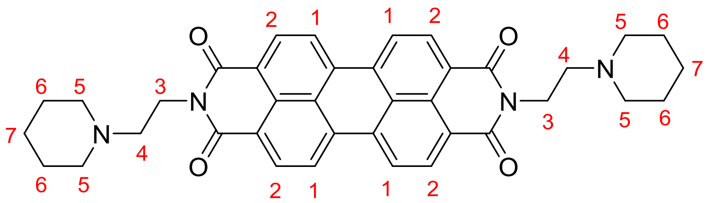

Following the reported procedure,[S15] to a solution of PTCDA (208 mg, 0.529 mmol) in water (5 mL) was added N-(3-aminopropyl)piperidine (1.00 mL, 6.29 mmol). The reaction mixture was stirred at rt for 24 h until complete consumption of the starting material was observed by MALDI (Product MH_2^{2+} = 614). The reaction mixture was filtered and washed with water (3 × 10 mL) to yield **PIPER** as a dark red solid (251 mg, 77 %). As ^{13}C-NMR has not been reported in $CDCl_3$, it is reported here. **^{1}H-NMR** (400 MHz, $CDCl_3$) δ 8.61 (4H, d, J = 8.0 Hz, H-2), 8.50 (4H, d, J = 8.1 Hz, H-1), 4.53 – 4.25 (4H, m, H-3), 2.70 (4H, app dd, J = 8.2, 6.5 Hz, H-4), 2.58 (8H, s, H-5), 1.59 (12H, q, J = 5.8, 5.3 Hz, H-6 & H-7); **^{13}C-NMR** (101 MHz, $CDCl_3$) δ 163.3 (C=O), 134.5 (Ar-C), 131.3 (C-2), 123.3 (Ar-C), 123.0 (C-1), 56.3 (C-4), 54.8 (C-5), 37.8 (C-3), 26.1 (C-6), 24.4 (C-7). Proton NMR was consistent with literature data.[S15]

To the free base (89.3 mg, 0.146 mmol) conc. HCl (4 mL) was added, and any solid was broken up with a glass rod. A brick red precipitate was observed, and water (1 mL) was added. The reaction mixture was filtered, washed with diethyl ether (3 × 10 mL) and dried to yield the quaternary ammonium salt of **PIPER** as a dark red solid (83.8 mg, 94 %). As ^{13}C-NMR has not been reported before, it is reported here. **^{1}H-NMR** (400 MHz, D_2O) δ 7.64 (4H, br s, H-2), 7.29 (4H, br s, H-1), 4.32 (4H, br s, H-3), 3.67 (4H, br s, H-5), 3.36 (4H, br s, H-4), 3.06 (4H, br s, H-5), 1.93 (4H, br s, H-6), 1.76 (6H, br s, H-6 & H-7), 1.49 (2H, br s, H-7); **^{13}C-NMR** (126 MHz, D_2O) δ 163.4 (C=O), 132.9 (Ar-C), 130.7 (C-2), 126.8 (Ar-C), 123.6 (C-1), 120.3 (Ar-C), 53.9 (C-5), 53.5 (C-4), 34.8, (C-3) 22.8 (C-6), 21.1 (C-7); **HPLC** (280 nm) t_R: 35.9 min, purity: >99 %. Proton NMR was consistent with literature data.[S15]

bis-*N,N′*-(1-(3-aminopropyl)-4-methylpiperazine)-3,4,9,10-perylenetetracarboxylic diimide (3)

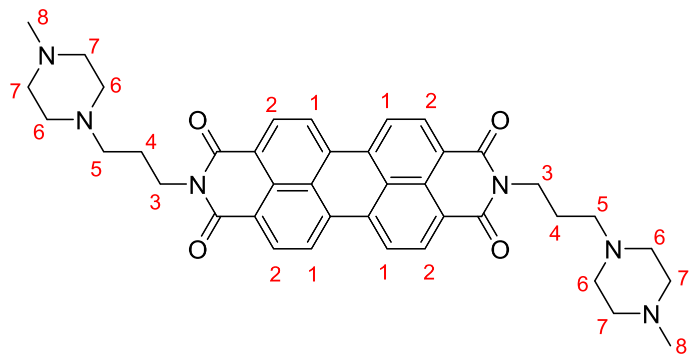

PTCDA (50.0 mg, 0.127 mmol), 1-(3-aminopropyl)-4-methylpiperazine (108 µl, 0.637 mmol) and imidazole (2.00 g, 29.4 mmol) were added under argon to a sealed tube. The tube was sealed and the reaction mixture was stirred and heated to 145 °C for 15 h until complete consumption of the starting materials was observed by LC-MS (Product MH^+ = 672). Once the reaction had reached temperature, the imidazole melted and the reaction formed a deep red solution. The reaction mixture was cooled to rt and acetone (10 mL) was added to dissolve the imidazole and precipitate the product. A red suspension was formed. The reaction mixture was filtered and a red solid was obtained. The red solid was dry-loaded in DCM and purified by flash chromatography (DCM / MeOH / TEA, 100:0:0 to 75:20:5) to yield imide **3** as a deep red solid with an orange fluorescence (33.2 mg, 39 %). **^{1}H-NMR** (400 MHz, $CDCl_3$) δ 8.66 (4H, d, *J* = 8.0 Hz, H-2), 8.59 (4H, d, *J* = 8.1 Hz, H-1), 4.33 – 4.24 (4H, m, H-3), 2.54 (4H, t, *J* = 7.1 Hz, H-5), 2.37 (16H, br s, H-6 & H-7), 2.20 (6H, s, H-8), 2.02 – 1.92 (4H, m, H-4); **^{13}C-NMR** (101 MHz, $CDCl_3$) δ 163.6 (C=O), 134.8 (Ar-C), 131.5 (C-2), 123.5 (Ar-C), 123.3 (C-1), 56.2 (C-5), 55.2 (C-7), 53.1 (C-6), 46.1 (C-8), 39.3 (C-3), 25.2 (C-4); **ESI-HRMS** for $C_{40}H_{43}N_6O_4^+$ (MH_2^{2+}) calcd: 671.3340; found: 671.3335.

To the free base (25.0 mg, 0.037 mmol) conc. HCl (1 mL) was added. A dark red precipitate was observed, which dissolved over 5 min to yield a viscous red oil in a red solution, and then a homogenous solution. The reaction mixture was concentrated *in vacuo*, washed with EtOAc (2 × 5 mL) and purified by reverse-phase flash chromatography (water + 0.1 % TFA / MeCN, 95:5 to 5:95) to yield the quaternary ammonium salt of imide **3** as a red solid (25.4 mg, 61 %). NMR analysis of the TFA counterion with 2-flurobenzoic acid reveals 4 equivalents of TFA present, indicating that the product is the tetra-salt. **^{1}H-NMR** (400 MHz, D_2O) δ 7.94 (8H, br s, H-1 & H-2), 4.13 (4H, br s, H-3), 3.61 (16H, br s, H-6 & H-7), 3.26 (4H, s, H-5), 3.03 (6H, br s, H-8), 2.16 (4H, br s, H-4); **^{13}C-NMR** (101 MHz, D_2O) δ 162.8 (q, *J* = 35.7 Hz, CF_3C=O),

130.3 (C-2), 123.2 (Ar-C), 120.7 (C-1), 116.3 (q, J = 290.7 Hz, CF$_3$C=O), 54.5 (C-5), 51.0 (C-7), 49.1 (C-6), 42.9 (C-8), 37.7 (C-3), 22.8 (C-4). Ar-C and C=O not observed; **^{19}F-NMR** (376 MHz, D_2O) δ -75.44 (CF_3C=O); **LC-MS** (MH^+ = 672) t_R: 0.7 min, purity: 99 %; **ESI-HRMS** for $C_{40}H_{43}N_6O_4^+$ (MH_2^{2+}) calcd: 671.3340; found: 671.3309; ν_{max} / cm^{-1} (film): 3675, 3432, 2979, 2903, 2409, 1778, 1662, 1593, 1443, 1402, 1343, 1255, 1178, 1122, 1019, 963, 831, 809, 797, 745, 720.

bis-*N,N′*-(1-(3-aminopropyl)-4-methylpiperazine)-3,3′,4,4′-biphenyltetracarboxylic diimide (4)

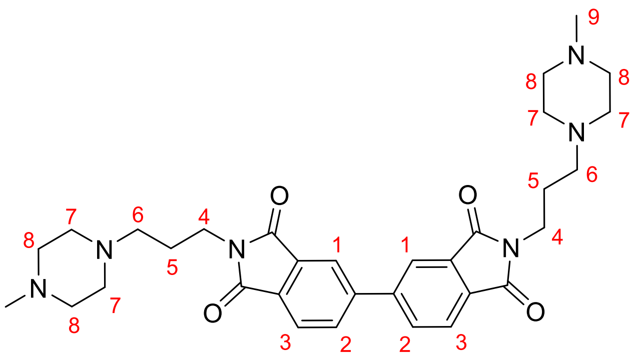

To a solution of BPDA (51.2 mg, 0.174 mmol) in toluene (5 mL) was added 1-(3-aminopropyl)-4-methylpiperazine (74.0 µL, 0.435 mmol) in a sealed tube. The tube was sealed and the reaction mixture was stirred and heated to 145 °C for 17 h until complete consumption of the starting materials was observed by LC-MS (Product MH^+ = 573). The reaction mixture was cooled to rt, concentrated *in vacuo* and purified by reverse-phase flash chromatography (water + 0.1 % TFA / MeCN, 95:5 to 5:95) to yield imide **4** as a light brown solid (110 mg, 79 %). NMR analysis of the TFA counterion with 2-flurobenzoic acid reveals 2 equivalents of TFA present, indicating that the product is the di-salt.**^{1}H-NMR** (400 MHz, D_2O) δ 8.14 - 8.03 (4H, m, H-1 & H-2), 7.98 - 7.89 (2H, m, H-3), 3.84 (4H, t, J = 6.6 Hz, H-4), 3.67 (16H, br s, H-7 & H-8), 3.43 - 3.34 (4H, m, H-6), 3.04 (6H, s, H-9), 2.26 - 2.13 (4H, m, H-5); **^{13}C-NMR** (101 MHz, D_2O) δ 169.7 (C=O), 162.9 (q, J = 35.3 Hz, CF$_3$$C$=O), 145.0 (Ar-C), 133.7 (C-2), 132.2 (Ar-C), 130.9 (Ar-C), 124.1 (C-3), 122.3 (C-1), 116.3 (q, J = 291.8 Hz, CF$_3$C=O), 54.5 (C-6), 50.4 (C-8), 48.8 (C-7), 42.8 (C-9), 34.8 (C-4), 23.0 (C-5); **^{19}F-NMR** (376 MHz, D_2O) δ -75.58 (CF_3C=O); **LC-MS** (MH^+ = 573) t_R: 0.5 min, purity: 98 %; **ESI-HRMS** for $C_{32}H_{41}N_6O_4^+$ (MH_2^{2+}) calcd: 287.1628; found: 287.1629; ν_{max} / cm^{-1} (film): 3443, 2986, 2392, 1770, 1706, 1668, 1443, 1392, 1365, 1176, 1121, 1020, 964, 831, 797, 740, 720.

bis-*N,N'*-(1-(3-aminopropyl)-4-methylpiperazine)-4,4''-oxydiphthalimide (5)

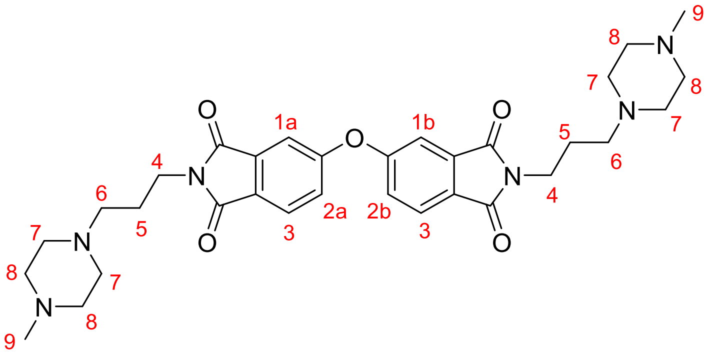

To a solution of ODPA (51.0 mg, 0.164 mmol) in toluene (5 mL) was added 1-(3-aminopropyl)-4-methylpiperazine (70.0 µL, 0.411 mmol) in a sealed tube. The tube was sealed and the reaction mixture was stirred and heated to 145 °C for 17 h until complete consumption of the starting materials was observed by LC-MS (Product MH^+ = 589). The reaction mixture was cooled to rt, concentrated *in vacuo* and purified by reverse-phase flash chromatography (water + 0.1 % TFA / MeCN, 9:1 to 5:95) to yield imide **5** as a cream solid (62.3 mg, 46 %). NMR analysis of the TFA counterion with 2-flurobenzoic acid reveals 2 equivalents of TFA present, indicating that the product is the di-salt. **^{1}H-NMR** (400 MHz, D_2O) δ 7.96 (1H, s, H-1a), 7.94 (1H, s, H-1b), 7.60 (2H, d, *J* = 2.0 Hz, H-3), 7.54 (1H, d, *J* = 2.1 Hz, H-2a), 7.52 (1H, d, *J* = 2.1 Hz, H-2b), 3.80 (4H, t, *J* = 6.7 Hz, H-4), 3.57 (16H, br s, H-7 & H-8), 3.27 - 3.19 (4H, m, H-6), 2.99 (6H, s, H-9), 2.13 (4H, dt, *J* = 14.7, 6.8 Hz, H-5); **^{13}C-NMR** (101 MHz, D_2O) δ 169.7 (C=O), 169.3 (C=O), 162.9 (q, *J* = 35.7 Hz, CF_3C=O), 161.1 (Ar-C), 134.2 (Ar-C), 126.9 (Ar-C), 125.9 (C-1), 124.7 (C-2), 116.2 (q, *J* = 290.7 Hz, CF_3C=O), 114.2 (C-3), 54.4 (C-6), 50.8 (C-8), 49.0 (C-7), 42.8 (C-9), 35.0 (C-4), 23.2 (C-5); **^{19}F-NMR** (376 MHz, D_2O) δ -75.59 (CF_3C=O); **LC-MS** (MH^+ = 589) t_R: 0.6 min, purity: 99 %; **ESI-HRMS** for $C_{32}H_{41}N_6O_5^+$ (MH_2^{2+}) calcd: 295.1602; found: 295.1603; ν_{max} / cm^{-1} (film): 3444, 2986, 2394, 1771, 1706, 1670, 1443, 1395, 1365, 1274, 1235, 1178, 1122, 1020, 963, 830, 797, 746, 720.

bis-*N,N′*-(1-(3-aminopropyl)-4-methylpiperazine)-4,4′-(hexafluoroisopropylidene)diphthalimide (6)

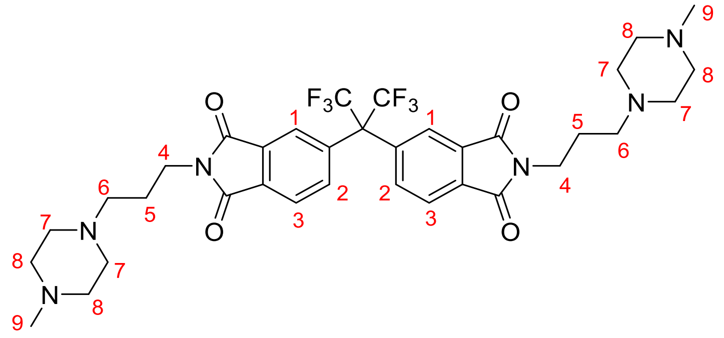

To a solution of 4,4′-(hexafluoroisopropylidene)diphthalic anhydride (51.5 mg, 0.116 mmol) in toluene (10 mL) was added 1-(3-aminopropyl)-4-methylpiperazine (49.0 µL, 0.290 mmol) in a sealed tube. The tube was sealed and the reaction mixture was stirred and heated to 145 °C for 17 h until complete consumption of the starting materials was observed by LC-MS (Product MH^+ = 723). The reaction mixture was cooled to rt, concentrated *in vacuo* and purified by reverse-phase flash chromatography (water + 0.1 % TFA / MeCN, 9:1 to 5:95) to yield imide **6** as a dark orange solid (71.3 mg, 65 %). NMR analysis of the TFA counterion with 2-flurobenzoic acid reveals 2 equivalents of TFA present, indicating that the product is the di-salt. **^{1}H-NMR** (400 MHz, D_2O) δ 8.02 (2H, s, H-1), 7.99 (2H, d, *J* = 8.1 Hz, H-3), 7.93 (2H, d, *J* = 8.1 Hz, H-2), 3.84 (4H, t, *J* = 6.7 Hz, H-4), 3.64 (16H, br s, H-7 & H-8), 3.36 - 3.28 (4H, m, H-6), 3.02 (6H, s, H-9), 2.22 - 2.12 (4H, m, H-5); **^{13}C-NMR** (101 MHz, D_2O) δ 169.3 (C=O), 169.2 (C=O), 162.9 (q, *J* = 35.6 Hz, CF_3*C*=O), 138.6 (Ar-C), 136.7 (C-2), 132.4 (Ar-C), 131.9 (Ar-C), 124.9 (C-1), 123.8 (C-3), 116.2 (q, *J* = 293.2 Hz, *C*F_3C=O), 64.9 (C), 54.4 (C-6), 50.4 (C-8), 48.9 (C-7), 42.8 (C-9), 35.0 (C-4), 22.9 (C-5). CF_3 not observed; **^{19}F-NMR** (376 MHz, D_2O) δ -63.82 (*C*F_3), -75.60 (C*F*$_3$C=O); **LC-MS** (MH^+ = 723) t_R: 0.7 min, purity: 98 %; **ESI-HRMS** for $C_{35}H_{41}F_6N_6O_4^+$ (MH_2^{2+}) calcd: 362.1580; found: 362.1573; **ν_{max}** / cm^{-1} (film): 3678, 3444, 2980, 2405, 1777, 1712, 1669, 1445, 1396, 1366, 1301, 1256, 1182, 1124, 1020, 964, 913, 831, 797, 745, 719.

bis-*N,N'*-(1-(3-aminopropyl)-4-methylpiperazine)-3,3',4,4'-benzophenonetetracarboxylic diimide (7)

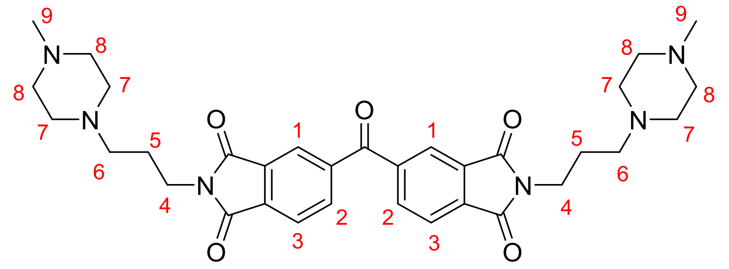

To a solution of BTDA (204 mg, 0.632 mmol) in toluene (20 mL) was added 1-(3-aminopropyl)-4-methylpiperazine (236 µL, 1.39 mmol). The reaction mixture was stirred and heated to 145 °C for 15 h until complete consumption of the starting materials was observed by LC-MS (Product MH^+ = 601). Pyridine (500 µL, 6.21 mmol) and Ac_2O (250 µL, 2.64 mmol) were added and the mixture was stirred at rt for a further 2 h until the reaction was determined to be complete by LC-MS (Product MH^+ = 601). The reaction mixture was concentrated *in vacuo* and purified by reverse-phase flash chromatography (water + 0.1 % TFA / MeCN, 95:5 to 5:95) to yield imide **7** as a brown solid (222 mg, 33 %). NMR analysis of the TFA counterion with 2-flurobenzoic acid reveals 4 equivalents of TFA present, indicating that the product is the di-salt. **^{1}H-NMR** (400 MHz, D_2O) δ 8.13 - 8.06 (4H, m, H-1 & H-2), 7.96 - 7.89 (2H, m, H-3), 3.74 (4H, t, *J* = 6.6 Hz, H-4), 3.59 (16 H, br s, H-7 & H-8), 3.35 - 3.26 (4H, m, H-6), 2.93 (6H, s, H-9), 2.10 (4H, p, *J* = 6.8 Hz, H-5); **^{13}C-NMR** (101 MHz, D_2O) δ 195.8 (C=O), 169.2 (N*C*=O), 169.1 (N*C*=O), 162.8 (q, *J* = 35.5 Hz, CF_3*C*=O), 141.4 (Ar-C), 136.6 (C-1), 135.0 (Ar-C), 131.7 (Ar-C), 124.5 (C-2), 123.8 (C-3), 116.3 (q, *J* = 291.9 Hz, *C*F_3C=O), 54.4 (C-6), 50.2 (C-8), 48.8 (C-7), 42.8 (C-9), 34.9 (C-4), 22.8 (C-5); **^{19}F-NMR** (376 MHz, D_2O) δ -75.56 (CF_3C=O); **LC-MS** (MH^+ = 601) t_R: 0.5 min, purity: 96 %; **ESI-HRMS** for $C_{33}H_{41}N_6O_5^+$ (MH_2^{2+}) calcd: 301.1602; found: 301.1592; **ν_{max}** / cm^{-1} (film): 3459, 2978, 2354, 1775, 1714, 1668, 1395, 2181, 1252, 1176, 1117, 1077, 1022, 967, 923, 867, 829, 796, 748, 720.

bis-*N,N'*-(1-(3-aminopropyl)-4-methylpiperazine)-pyromellitic diimide (8)

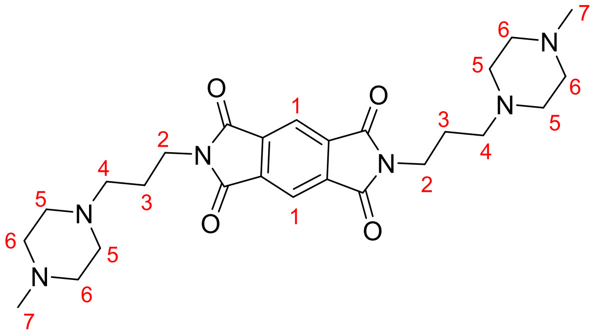

To a solution of PMDA (51.0 mg, 0.234 mmol) in toluene (5 mL) was added 1-(3-aminopropyl)-4-methylpiperazine (99.0 µL, 0.585 mmol) in a sealed tube. The tube was sealed and the reaction mixture was stirred and heated to 145 °C for 17 h until complete consumption of the starting materials was observed by LC-MS (Product MH^+ = 497.3). The reaction mixture was cooled to rt, concentrated *in vacuo* and purified by reverse-phase flash chromatography (water + 0.1 % TFA / MeCN, 95:5 to 5:95) to yield imide **8** as a light brown solid (103 mg, 46 %). NMR analysis of the TFA counterion with 2-flurobenzoic acid reveals 4 equivalents of TFA present, indicating that the product is the tetra-salt. **^{1}H-NMR** (400 MHz, D_2O) δ 8.35 (2H, s, H-1), 3.88 (4H, t, J = 6.8 Hz, H-2), 3.68 (16H, br s, H-5 & H-6), 3.43 - 3.34 (4H, m, H-4), 3.04 (6H, s, H-7), 2.21 (4H, dt, J = 14.8, 6.9 Hz, H-3); **^{13}C-NMR** (101 MHz, D_2O) δ 168.1 (C=O), 163.1 (app d, J = 69.8 Hz, CF_3*C*=O), 137.2 (Ar-C), 118.3 (C-1), 116.3 (q, J = 288.3 Hz *C*F_3C=O), 54.4 (C-4), 50.3 (C-6), 48.8 (C-5), 42.8 (C-7), 35.2 (C-2), 22.8 (C-3); **^{19}F-NMR** (376 MHz, D_2O) δ -75.60 (C*F_3*C=O); **LC-MS** (MH^+ = 497) t_R: 0.35 min, purity: 97 %; **ESI-HRMS** for $C_{26}H_{37}N_6O_4^+$ (MH_2^{2+}) calcd: 249.1471; found: 249.1467; ν_{max} / cm^{-1} (film): 3660, 3459, 2982, 2900, 2425, 1774, 1714, 1691, 1678, 1462, 1392, 1368, 1307, 1285, 1198, 1156, 1122, 1073, 1038, 1020, 965, 885, 833, 794, 721.

bis-*N,N′*-(1-(3-aminopropyl)-4-methylpiperazine)-4,4′-(4,4′-isopropylidenediphenoxy)bis(phthalimide) (9)

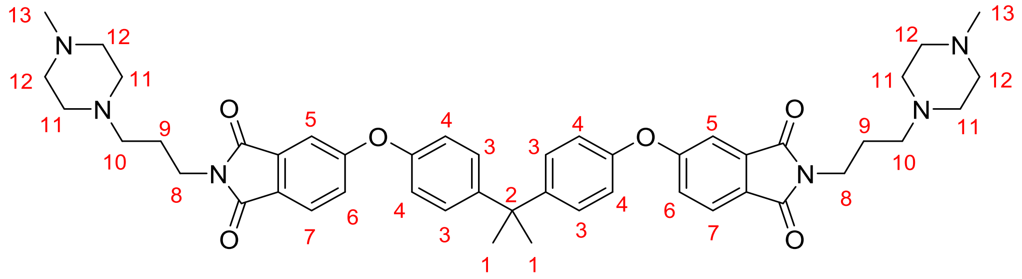

To a solution of 4,4′-(4,4′-isopropylidenediphenoxy)bis(phthalic anhydride) (200 mg, 0.384 mmol) and triethylamine (600 µL, 4.30 mmol) in DMSO (6 mL) was added 1-(3-aminopropyl)piperazine (150 µL, 0.881 mmol) in a sealed tube. The tube was sealed and the reaction mixture was stirred and heated to 80 °C for 18 h until complete consumption of the starting materials was observed by TLC (DCM / MeOH, 9:1). The reaction mixture was cooled to rt, concentrated by lyophilisation and purified by flash chromatography (DCM / MeOH, 95:5 to 8:2) to yield imide **9** as a yellow oil (50.0 mg, 16 %). **^{1}H-NMR** (500 MHz, $CDCl_3$) δ 7.75 (2H, d, J = 8.2 Hz, H-5), 7.31 – 7.23 (8H, m, H-4, H-6 & H-7), 6.98 (4H, d, J = 8.4 Hz, H-3), 3.70 (4H, t, J = 6.9 Hz, H-8), 2.62 – 2.06 (16H, br s, H-11 & H-12), 2.38 (4H, t, J = 6.9 Hz, H-10), 2.18 (6H, s, H-13), 1.81 (4H, p, J = 6.9 Hz, H-9), 1.72 (6H, s, H-1); **^{13}C-NMR** (125 MHz, $CDCl_3$) δ 168.0 (C=O), 163.4 (C=O), 152.9 (Ar-C), 147.5 (Ar-C), 134.9 (Ar-C), 128.7 (C-4), 125.8 (Ar-C), 125.1 (C-5), 122.4 (C-6), 120.0 (C-3), 111.6 (C-7), 56.0 (C-10), 55.1 (C-12), 53.1 (C-11), 46.1 (C-13), 42.6 (C-2), 36.8 (C-8), 31.1 (C-1), 25.4 (C-9); **ESI-HRMS** for $C_{47}H_{55}N_6O_6^+$ (MH^+) calcd: 799.4178; found: 799.4159; **ν_{max}** / cm^{-1} (film): 2937, 2794, 1769, 1706, 1619, 1600, 1504, 1475, 1446, 1393, 1273, 1232, 1168; **HPLC** (230 nm) t_R: 4.5 min, purity: >99 % (injected in DMSO).

4,4'-carbonylbis(N1,N2-bis(3-(4-methylpiperazin-1-yl)propyl)phthalamide) (10)

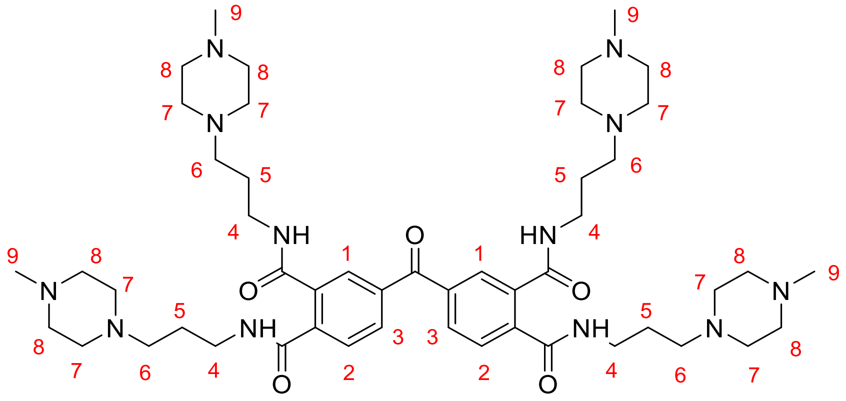

To a solution of BTDA (51.0 mg, 0.158 mmol) in toluene (10 mL) was added 1-(3-aminopropyl)-4-methylpiperazine (135 µL, 0.791 mmol) in a sealed tube. The tube was sealed and the reaction mixture was stirred and heated to 145 °C for 15 h until complete consumption of the starting materials was observed by LC-MS (Product MH_2^{2+} = 458). The reaction mixture was cooled to rt, concentrated *in vacuo* and purified by reverse-phase flash chromatography (water + 0.1 % TFA / MeCN, 9:1 to 5:95) to yield amide **10** as a viscous orange oil (117 mg, 40 %). NMR analysis of the TFA counterion with 2-flurobenzoic acid reveals 8 equivalents of TFA present, indicating that the product is the octa-salt. **^{1}H-NMR** (400 MHz, D_2O) δ 7.92 (2H, s, H-1), 7.86 (2H, d, J = 8.0 Hz, H-3), 7.64 (2H, d, J = 7.9 Hz, H-2), 3.57 (32H, br s, H-7 & H-8), 3.39 (8H, q, J = 7.0 Hz, H-4), 3.30 - 3.21 (8H, m, H-6), 2.92 (12H, s, H-9), 2.06 - 1.95 (8H, m, H-5); **^{13}C-NMR** (101 MHz, D_2O) δ 196.5 (C=O), 170.6 (NH*C*=O), 163.0 (q, J = 34.8 Hz, CF_3*C*=O), 138.4 (Ar-C), 138.0 (Ar-C), 134.9 (Ar-C), 132.9 (C-3), 128.7 (C-1), 128.1 (C-2), 116.3 (q, J = 295.2 Hz, *C*F_3C=O), 54.5 (C-6), 50.4 (C-8), 48.8 (C-7), 42.8 (C-9), 36.7 (C-4), 23.6 (C-5); **^{19}F-NMR** (376 MHz, D_2O) δ -75.58 (CF_3C=O); **LC-MS** (MH_2^{2+} = 458) t_R: 0.2 min, purity: 99 %; **ESI-HRMS** for $C_{49}H_{79}N_{12}O_5^+$ (MH_2^{2+}) calcd: 458.3181; found: 458.3174; **ν_{max}** / cm^{-1} (film): 3438, 3311, 3014, 2454, 1776, 1662, 1548, 1461, 1420, 1314, 1252, 1122, 1179, 1019, 964, 832, 798, 720.

4,4'-((propane-2,2-diylbis(4,1-phenylene))bis(oxy))bis(N1,N2-bis(3-(4-methylpiperazin-1-yl)propyl)phthalamide) (11)

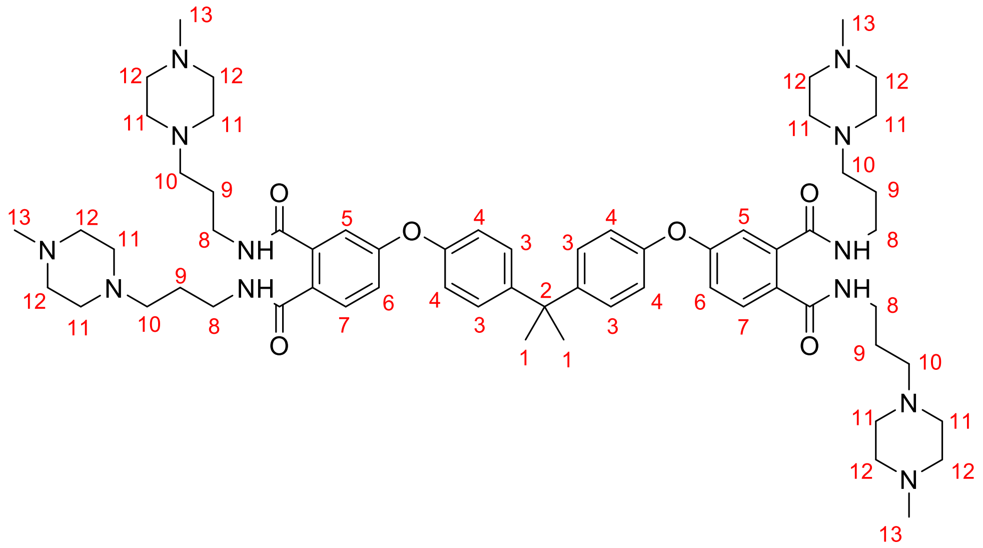

To a solution of 4,4′-(4,4′-isopropylidenediphenoxy)bis(phthalic anhydride) (51.0 mg, 0.098 mmol) in toluene (5 mL) was added 1-(3-aminopropyl)-4-methylpiperazine (83.0 µL, 0.490 mmol) in a sealed tube. The tube was sealed and the reaction mixture was stirred and heated to 145 °C for 15 h until complete consumption of the starting materials was observed by LC-MS (Product MH_2^{2+} = 558). The reaction mixture was cooled to rt, concentrated *in vacuo* and purified by reverse-phase flash chromatography (water + 0.1 % TFA / MeCN, 95:5 to 5:95) to yield amide **11** as a viscous yellow oil (149 mg, 75 %). NMR analysis of the TFA counterion with 2-flurobenzoic acid reveals 8 equivalents of TFA present, indicating that the product is the octa-salt. **^{1}H-NMR** (400 MHz, D_2O) δ 7.59 (2H, d, *J* = 8.5 Hz, H-7), 7.43 (4H, d, *J* = 8.6 Hz, H-3), 7.22 (2H, app d, *J* = 2.1 Hz, H-5), 7.17 - 7.08 (6H, m, H-4 & H-6), 3.70 (32H, br s, H-11 & H-12), 3.46 (8H, q, *J* = 6.9 Hz, H-8), 3.41 - 3.34 (8H, m, H-10), 3.05 (12H, s, H-13), 2.10 (8H, app dp, *J* = 11.4, 6.5 Hz, H-9), 1.74 (6H, s, H-1); **^{13}C-NMR** (101 MHz, D_2O) δ 171.2 (C=O), 170.9 (C=O), 163.0 (q, *J* = 36.2, 35.7 Hz, CF_3*C*=O), 159.3 (Ar-C), 153.2 (Ar-C), 147.5 (Ar-C), 136.7 (Ar-C), 129.9 (C-7), 128.6 (C-3), 119.5 (C-4), 119.0 (C-6), 116.3 (q, *J* = 294.2 Hz, *C*F_3C=O), 117.4 (C-5), 54.5 (C-10), 50.3 (C-12), 48.8 (C-11), 42.8 (C-13), 41.9 (C-2), 36.6 (C-8), 30.0 (C-1), 23.5 (C-9); **^{19}F NMR** (376 MHz, D_2O) δ -75.58 (CF_3C=O); **LC-MS** (MH_2^{2+} = 558) t_R: 0.5 min, purity: 99 %; **ESI-HRMS** for $C_{63}H_{93}N_{12}O_6^+$ (MH_2^{2+}) calcd: 557.3704; found: 557.3720; **ν_{max}** / cm^{-1} (film): 3429, 3268, 2975, 2450, 1668, 1504, 1481, 1463, 1417, 1316, 1278, 1234, 1123, 1176, 1016, 961, 833, 798, 720.

Supplementary Tables & Figures

Table S1. DNA Stabilization of **1** and **3-11** assessed by FRET Melting Assay at 10 µM concentration (ΔT_m reported to 1 °C using ΔT_{max}, error is reported as σ). Buffers used were as follows: F21T-K^+, Febr1T and F10T were 10 mM KCl, 90 mM LiCl and 10 mM Li cacodylate; FmycT was 1 mM KCl, 99 mM LiCl and 10 mM Li cacodylate; F21T-Na^+ was 100 mM NaCl and 10 mM Li cacodylate. Data for **1** against non-parasitic G4s has been previously reported and is reproduced with permission.[S16] The best results are highlighted. Data for **3** and **PIPER** is presented for information only, as spectral overlap prevents inferences about DNA stabilization.

Compound	F21T-K^+	F21T-Na^+	FmycT-K^+	Febr1T-K^+	F10T-K^+
**PIPER**[a]	**>40**[a]	**22 ± 1**	**26 ± 1**	**38 ± 7**	**1 ± 1**
**1**	**20 ± 1**	**2 ± 1**	**13 ± 1**	**11 ± 3**	**3 ± 1**
**3**[a]	**33 ± 1**	**>40** [a]	**>40** [a]	**39 ± 1**	**13 ± 2**
**4**	1 ± 1	-2 ± 2	2 ± 1	3 ± 1	0 ± 1
**5**	-3 ± 1	-3 ± 1	-3 ± 1	1 ± 1	0 ± 1
**6**	-2 ± 1	-8 ± 1	-4 ± 1	2 ± 1	0 ± 1
**7**	-3 ± 1	-3 ± 2	-3 ± 1	1 ± 1	0 ± 1
**8**	1 ± 1	1 ± 1	-2 ± 1	-2 ± 1	0 ± 1
**9**	**8 ± 3**	**6 ± 4**	**10 ± 3**	**-3 ± 1**	**0 ± 1**
**10**	-2 ± 1	-2 ± 1	-1 ± 1	1 ± 2	0 ± 1
**11**	**9 ± 1**	**9 ± 2**	**10 ± 2**	**13 ± 1**	**1 ± 1**

[a] Spectral overlap between the ligand and the FAM/TAMRA FRET pair may interfere with these results. Additionally, the oligonucleotide did not completely unfold under these conditions. These results are therefore not reliable indications of DNA Stabilization.

Table S2. DNA Stabilization of **Doxorubicin**, **TMPyP4**, **Pyridostatin**, **PIPER**, **1**, **3** and **11** assessed by FRET Melting Assay at 1 µM concentration (ΔT_m reported to 1 °C using ΔT_{max}, error is reported as σ). Buffers used were as follows: F21T-K^+, Febr1T and F10T were 10 mM KCl, 90 mM LiCl and 10 mM Li cacodylate; FmycT was 1 mM KCl, 99 mM LiCl and 10 mM Li cacodylate; F21T-Na^+ was 100 mM NaCl and 10 mM Li cacodylate. Data for 1 against non-parasitic G4s has been previously reported and is reproduced with permission.[S16] Data for **3** and **PIPER** is presented for information only, as spectral overlap prevents inferences about DNA stabilization.

Compound	F21T-K^+	F21T-Na^+	FmycT-K^+	Febr1T-K^+	F10T-K^+
**Doxorubicin**	10 ± 1	0 ± 1	8 ± 1	5 ± 1	2 ± 1
**TMPyP4**	33 ± 1	31 ± 2	34 ± 1	32 ± 2	9 ± 1
**Pyridostatin**	34 ± 1	36 ± 2	20 ± 3	29 ± 2	1 ± 1
**PIPER**[a]	33 ± 1	17 ± 2	23 ± 1	21 ± 4	0 ± 1
**1**	10 ± 1	-2 ± 1	0 ± 1	7 ± 3	0 ± 1
**3**[a]	10 ± 1	18 ± 2	24 ± 1	2 ± 1	1 ± 1
**11**	-2 ± 1	-4 ± 1	-3 ± 1	1 ± 1	0 ± 1

[a] Spectral overlap between the ligand and the FAM/TAMRA FRET pair may interfere with these results. Additionally, the oligonucleotide did not completely unfold under these conditions. These results are therefore not reliable indications of DNA Stabilization.

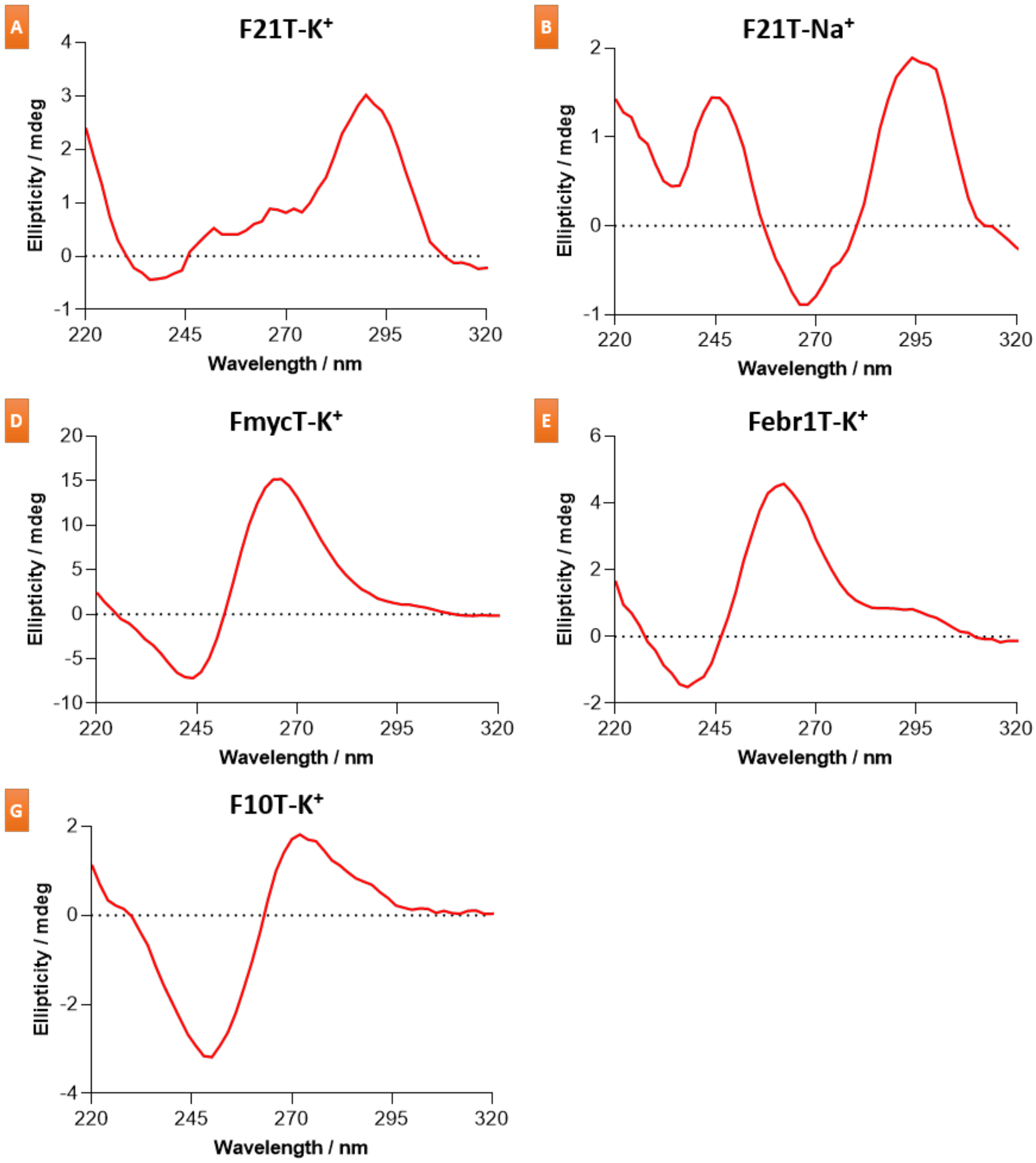

Figure S1. CD spectra of dual labelled oligonucleotides under conditions used in FRET experiments. Oligonucleotide concentration is 10 mM in all cases. Buffers used were as follows: F21T-K^+, Febr1T and F10T were 10 mM KCl, 90 mM LiCl and 10 mM Li cacodylate; FmycT was 1 mM KCl, 99 mM LiCl and 10 mM Li cacodylate; F21T-Na^+ was 100 mM NaCl and 10 mM Li cacodylate.

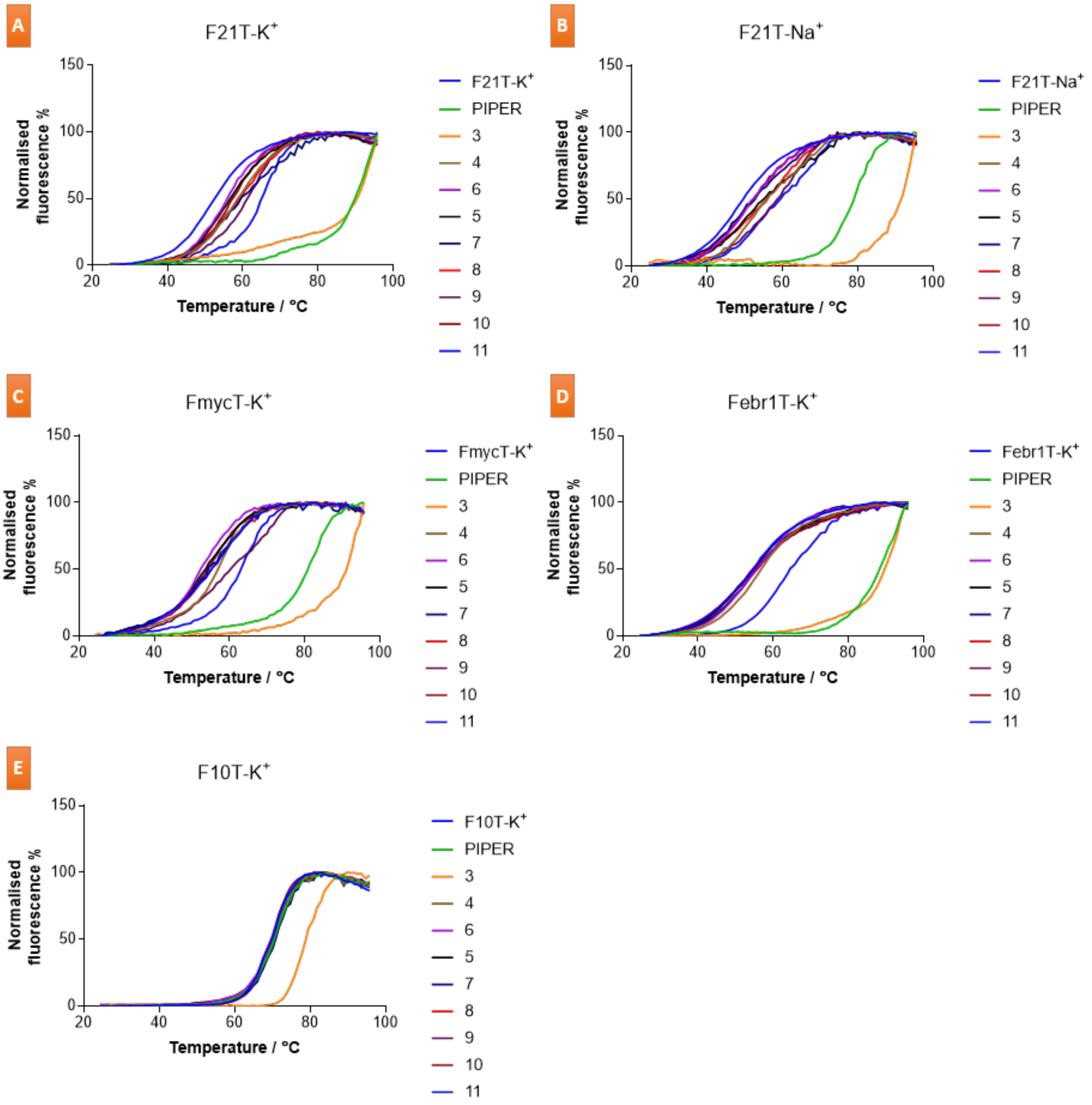

Figure S2. Raw FRET melting assay curves for all DNA structures and compounds examined in this work, at 10 µM ligand concentration. Oligonucleotide concentration was 200 nM in all cases. A) F21T-K^+ G-quadruplex, B) F21T-Na^+ G-quadruplex, C) FmycT-K^+ G-quadruplex, D) Febr1T-K^+ G-quadruplex, E) F10T-K^+ hairpin duplex. It should be noted that the fluorescence profile of **PIPER** and **3** overlaps with the fluorescence profile of the FAM/TAMRA FRET pair and are thus capable of interfering with the assay. For this reason, other methods (CD, UV-Vis, Fluorescence, NMR) were used to confirm the apparent DNA stabilization observed.

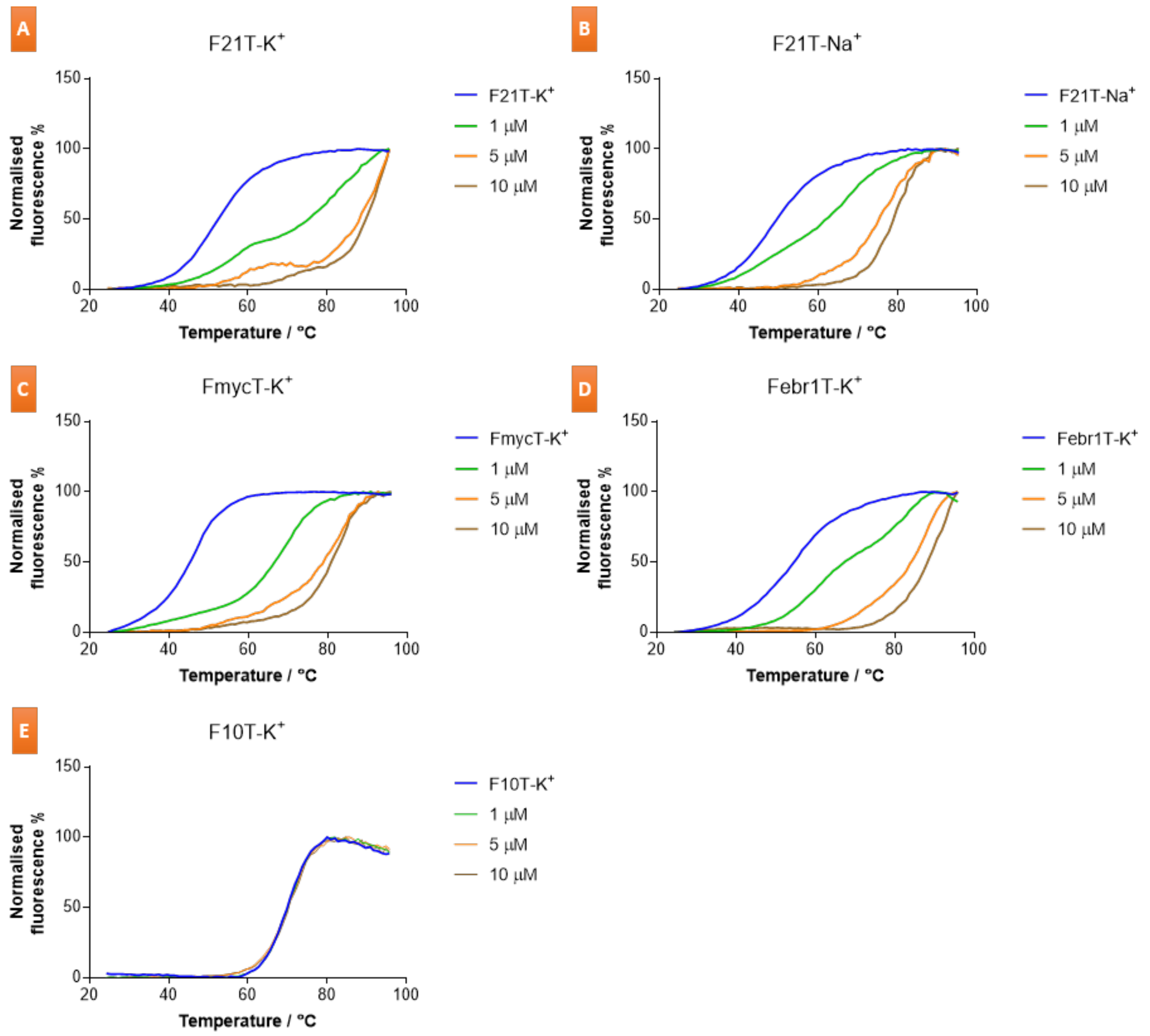

Figure S3. Raw FRET melting assay curves for **PIPER** at all concentrations against all DNA structures examined in this work. A) F21T-K^+ G-quadruplex, B) F21T-Na^+ G-quadruplex, C) FmycT-K^+ G-quadruplex, D) Febr1T-K^+ G-quadruplex, E) F10T-K^+ hairpin duplex.

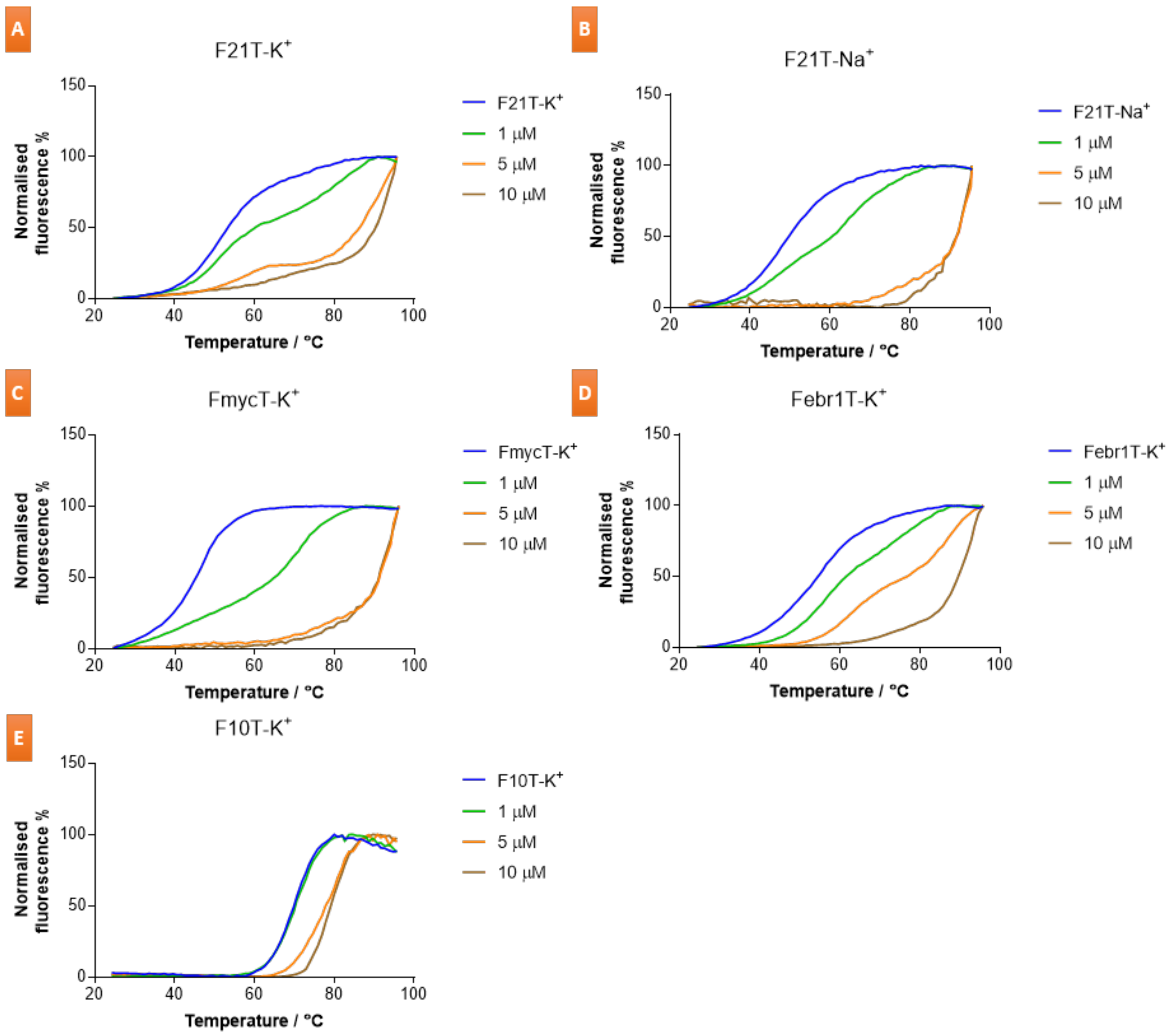

Figure S4. Raw FRET melting assay curves for **3** at all concentrations against all DNA structures examined in this work. A) F21T-K^+ G-quadruplex, B) F21T-Na^+ G-quadruplex, C) FmycT-K^+ G-quadruplex, D) Febr1T-K^+ G-quadruplex, E) F10T-K^+ hairpin duplex.

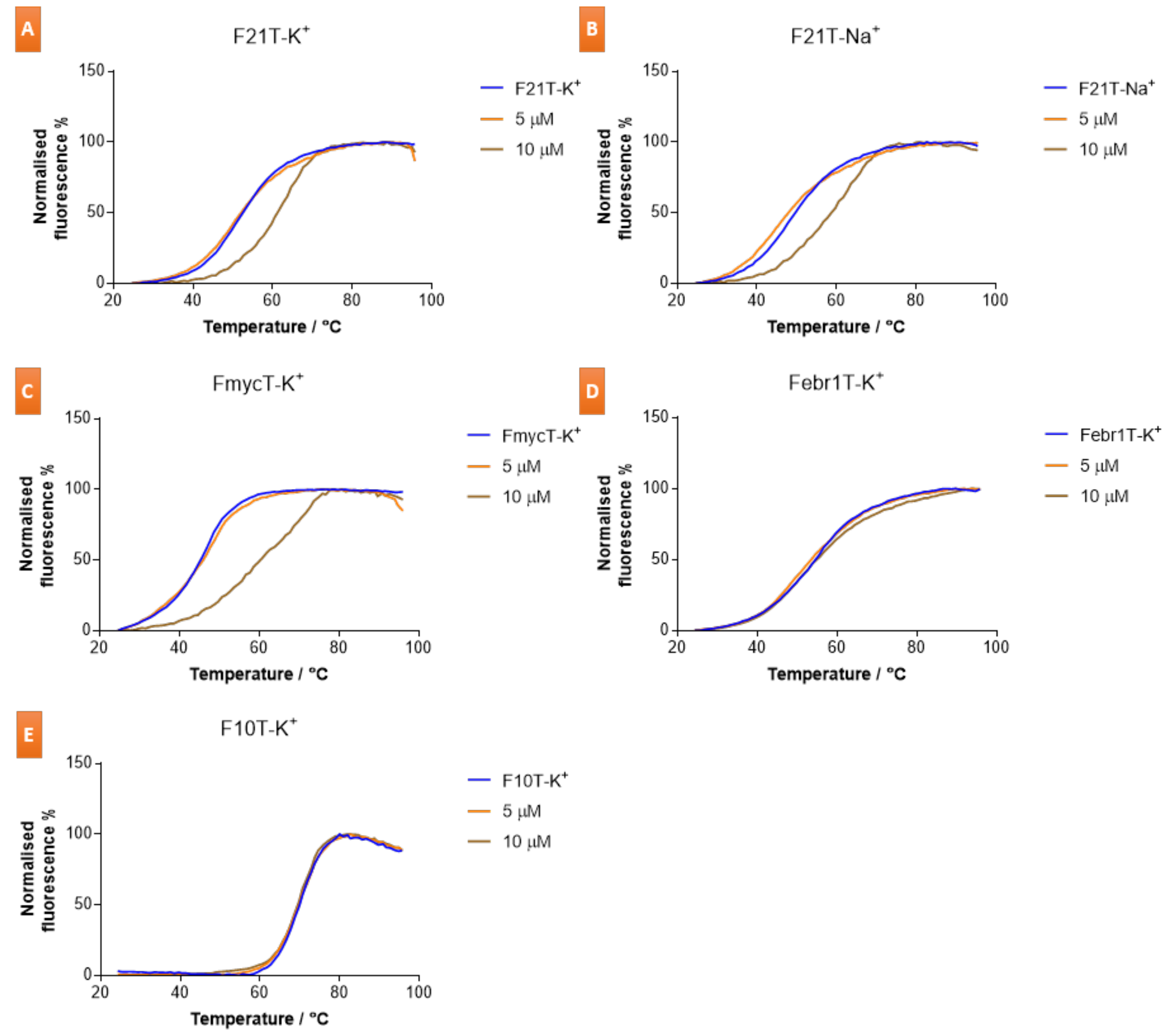

Figure S5. Raw FRET melting assay curves for **9** at 5 and 10 μM concentration against all DNA structures examined in this work. A) F21T-K^+ G-quadruplex, B) F21T-Na^+ G-quadruplex, C) FmycT-K^+ G-quadruplex, D) Febr1T-K^+ G-quadruplex, E) F10T-K^+ hairpin duplex.

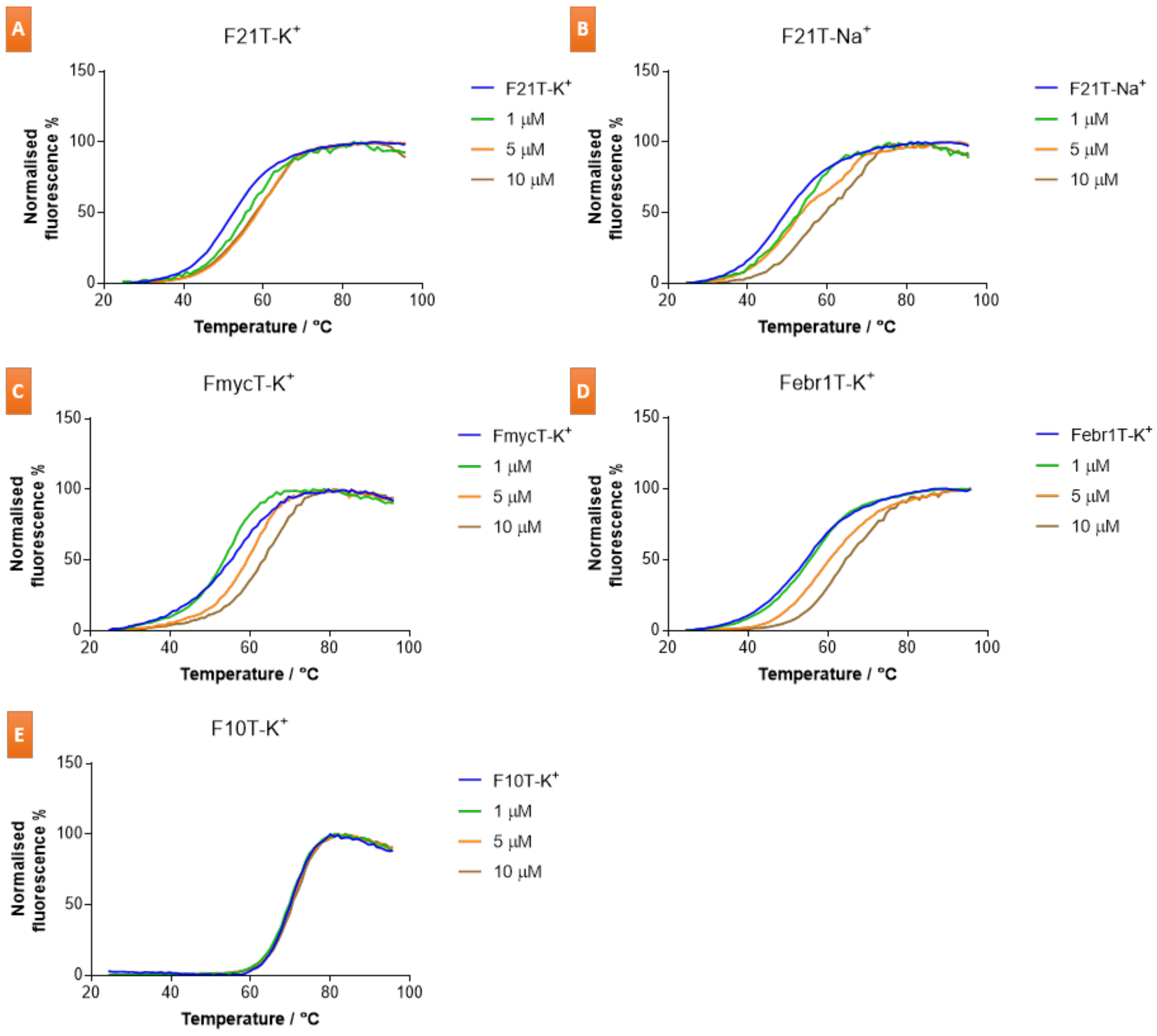

Figure S6. Raw FRET melting assay curves for **11** at all concentrations against all DNA structures examined in this work. A) F21T-K^+ G-quadruplex, B) F21T-Na^+ G-quadruplex, C) FmycT-K^+ G-quadruplex, D) Febr1T-K^+ G-quadruplex, E) F10T-K^+ hairpin duplex.

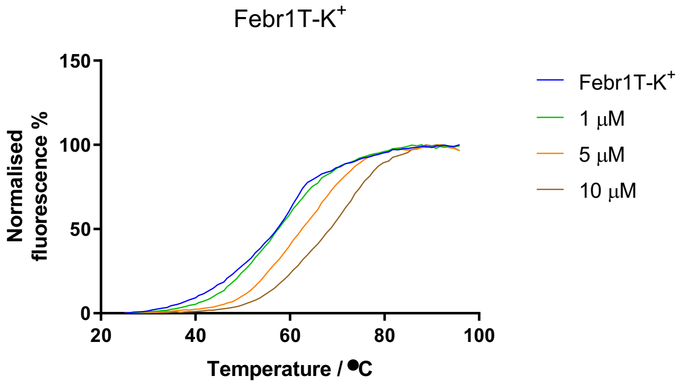

Figure S7. Raw FRET melting assay curves for **1** at all concentrations against the Febr1T-K^+ G-quadruplex.

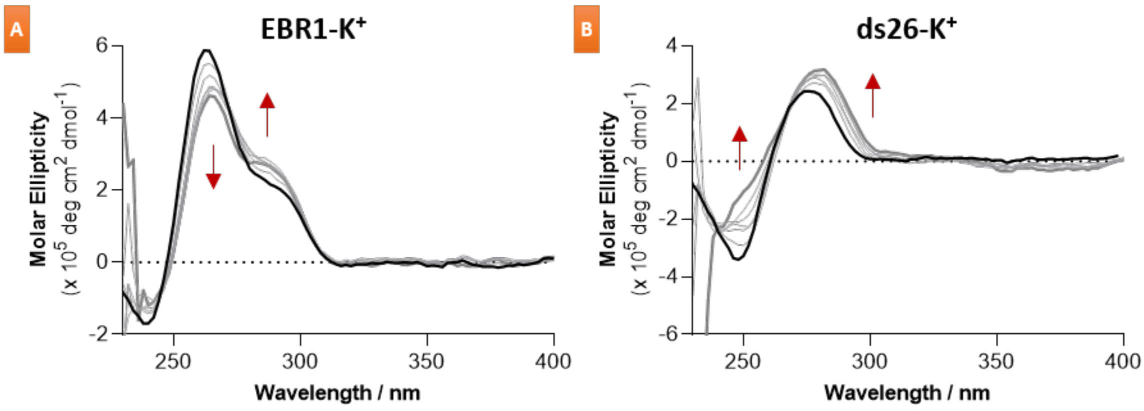

Figure S8. CD titration of **1** with (A) EBR1-K^+ G-quadruplex and (B) ds26-K^+ duplex DNA structures. Red arrows denote changes in molar ellipticity observed. Oligonucleotide concentration was 5 μM, with ligand concentration varied up to 10 equivalents (50 μM). CD titrations for telo23-K^+ and telo22-Na^+ have been previously reported.[S16]

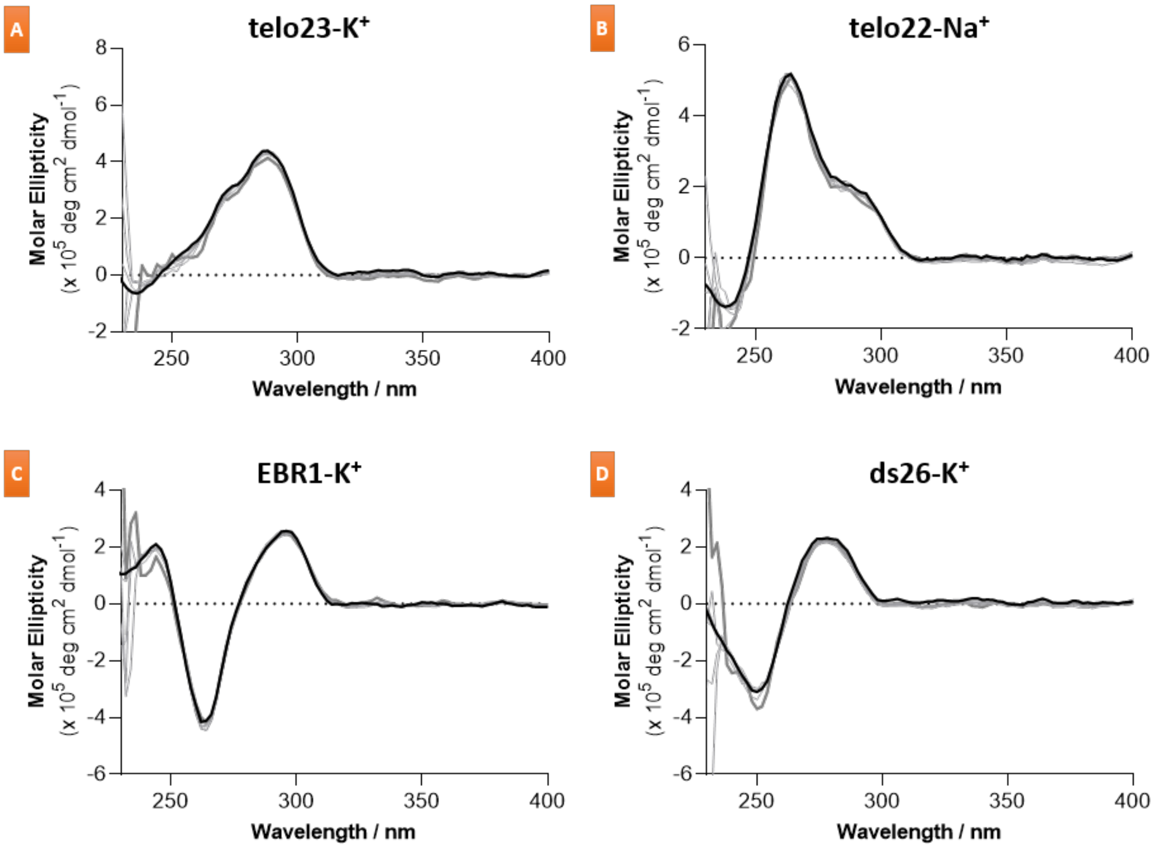

Figure S9. CD titration of **9** with (A) telo23-K^+, (B) telo22-Na^+, and (C) EBR1-K^+ G-quadruplex DNA, and (D) ds26-K^+ Duplex DNA. Oligonucleotide concentration was 5 µM, with ligand concentration varied up to 10 equivalents (50 µM).

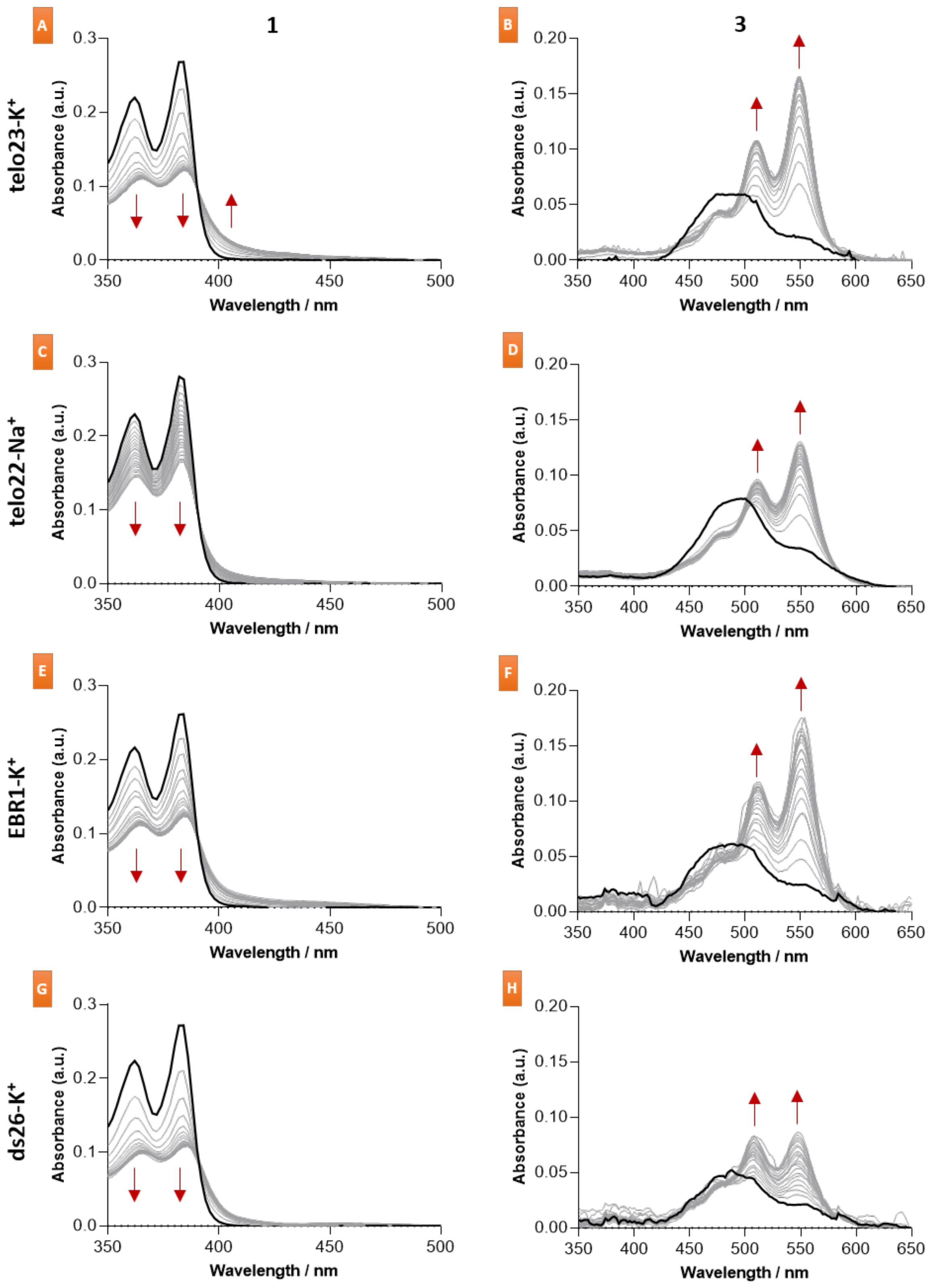

Figure S10. UV/Vis titrations for the association between compounds **1** (A, C, E, G) and **3** (B, D, F, H) with telo23-K^+ (A-B), telo22-Na^+ (C-D), EBR1-K^+ (E-F) G-quadruplex and ds26-K^+ (G-H) duplex DNA structures. Ligand concentration was 10 μM, with oligonucleotide concentration varied up to 30 μM.

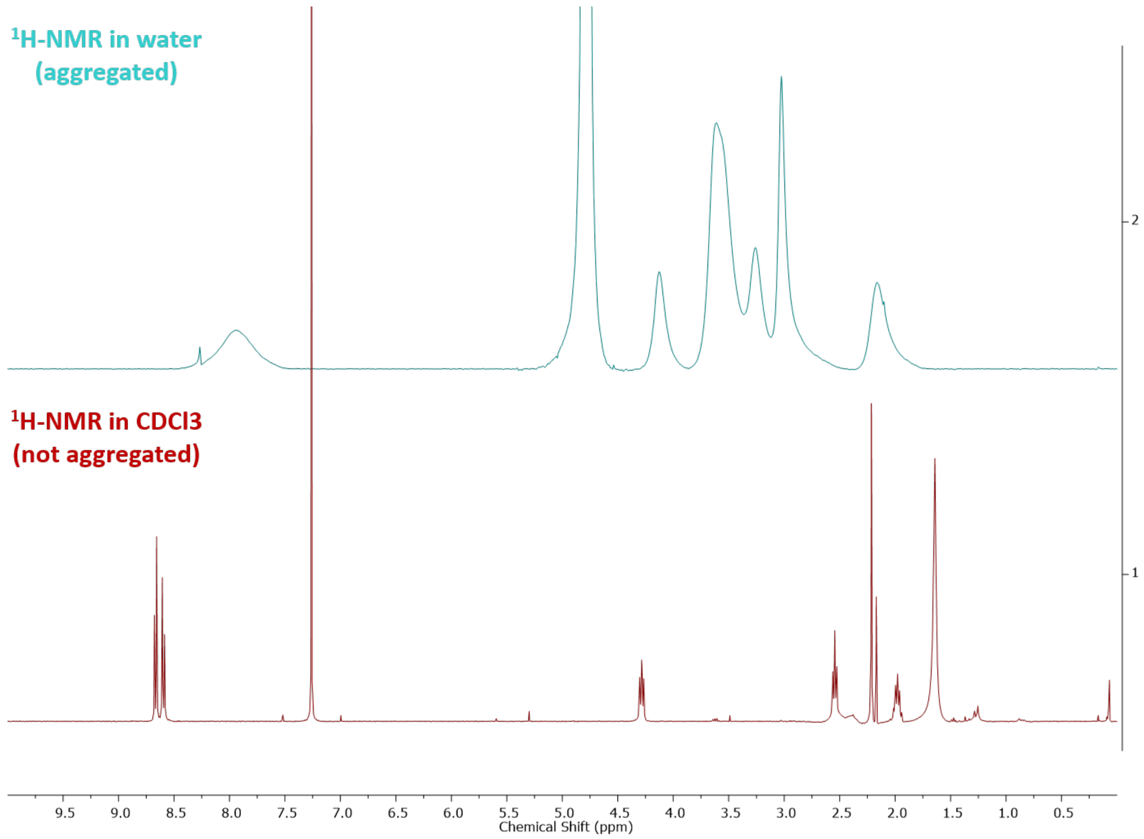

Figure S11. Comparison of the ^{1}H-NMR spectrum of **3** in water (4^+ charge, cyan spectrum) with the spectrum in chloroform-*d* (no charge, red spectrum). Compared to chloroform-*d*, in water significant line-broadening is observed which is consistent with aggregation in solution.

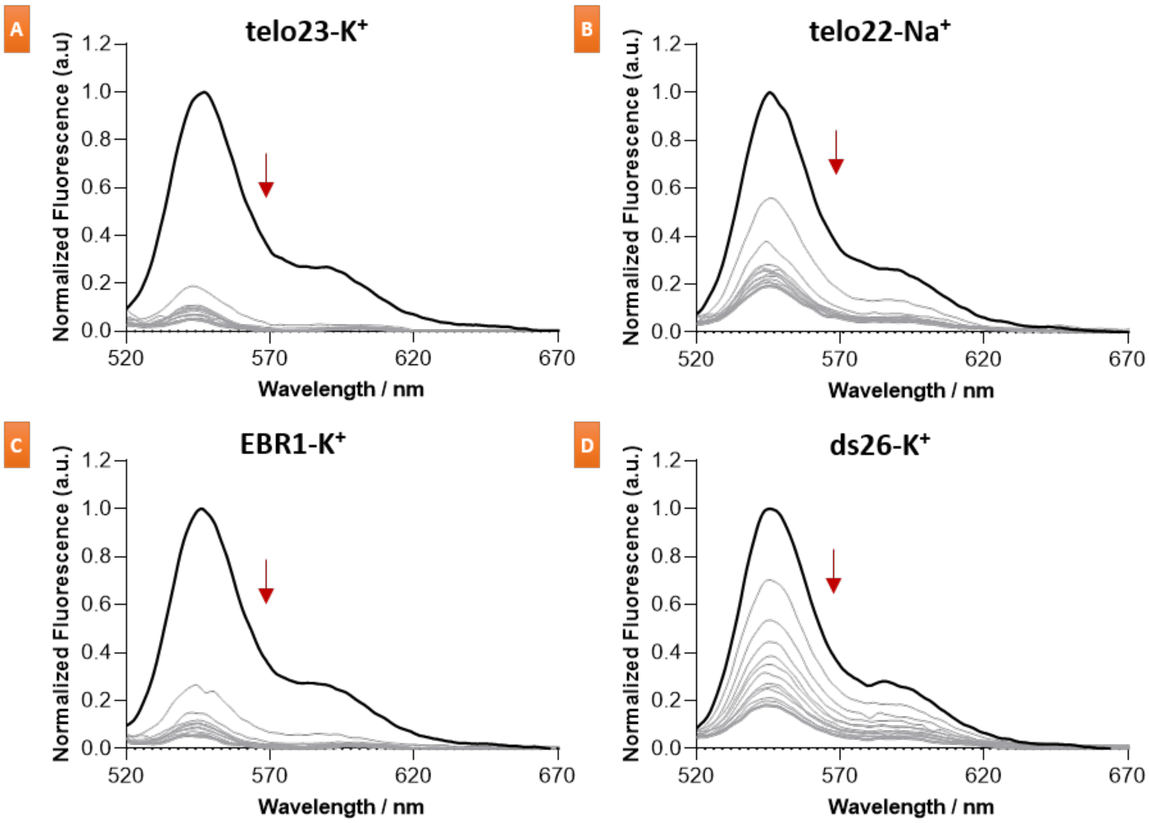

Figure S12. Normalized fluorescence emission spectra of **3** (1 µM) in the presence of up to 1 µM of (A) telo23-K^+, (B) telo22-Na^+, and (C) EBR1-K^+ G-quadurplex and (D) ds26-K^+ DNA.

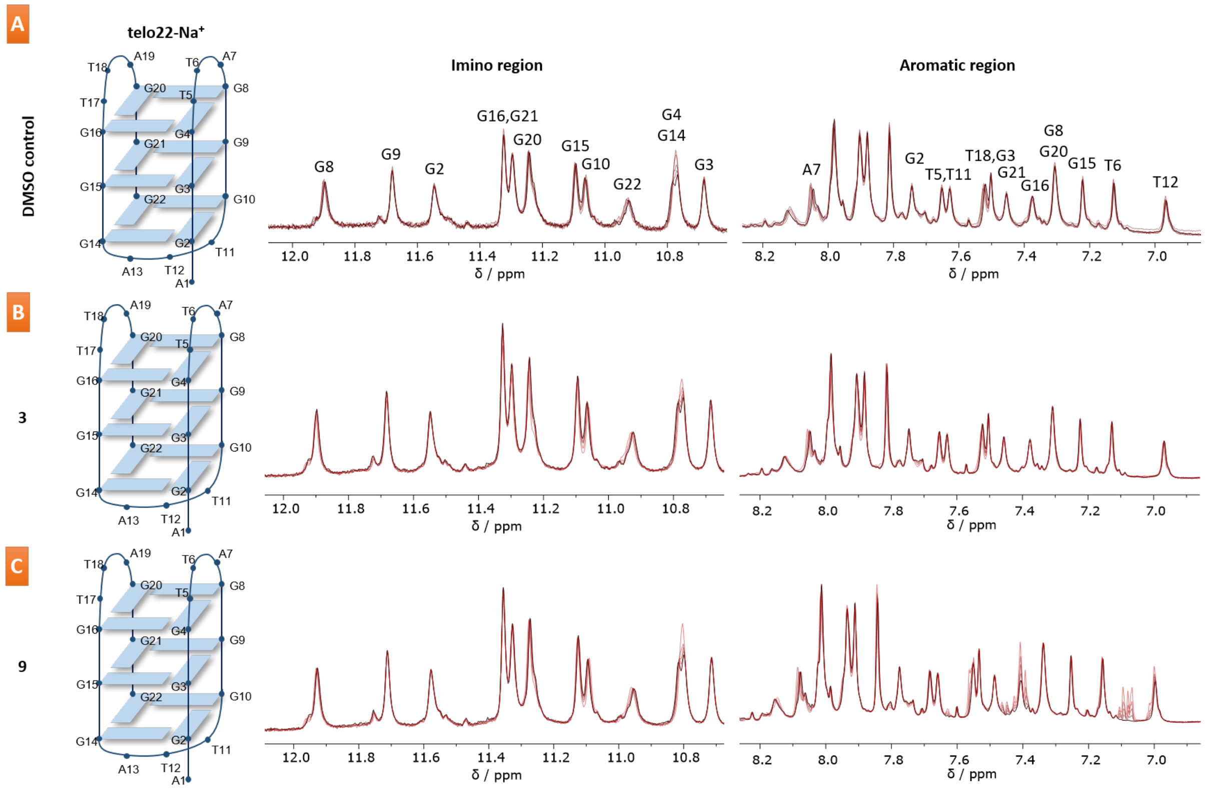

Figure S13. (A) NMR titration of the telo22-Na^+ G-quadruplex with DMSO as a control. (B-C) NMR titrations of (B) **3** and (C) **9** with the telo22-Na^+ G-quadruplex. Telo22-Na^+ concentration was 185 µM in all cases, with 0 (black trace), 0.25, 0.50, 1, and 2 equivalents of ligand added (red traces). Solutions were prepared in 25mM sodium phosphate buffer, pH 7.0 containing 70mM sodium chloride and 10% D_2O.

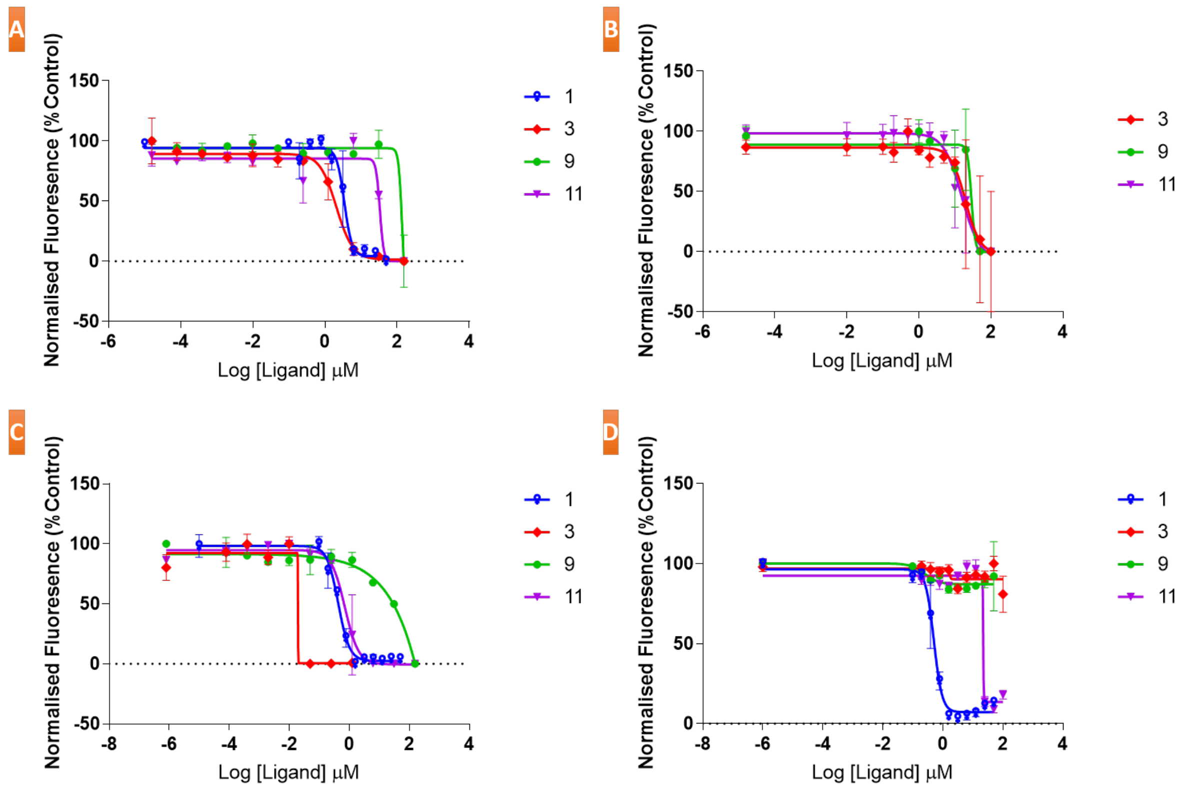

Figure S14. Dose-response curves used to determine EC_{50} values for the cytotoxicity of compounds **1**, **3**, **9** and **11** against (A) MRC5 cells, (B) HeLa cells, (C) *T. brucei* and (D) *L. major*. Data was processed in GraphPad Prism 7 using the 'log(inhibitor) vs. response -- Variable slope (four parameters)' nonlinear regression analysis.

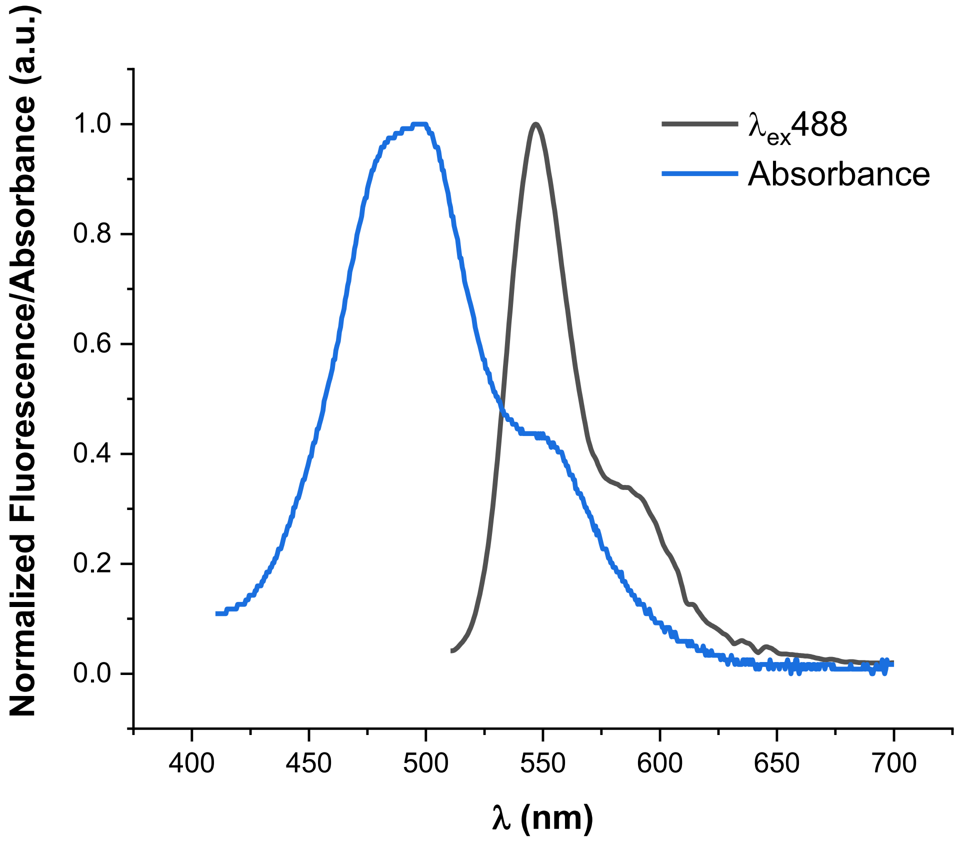

Figure S15. Fluorescence emission of **3** following excitation at 488nm (in black), and UV/Vis absorbance spectra (in blue) of **3** (25 μM) in PBS at pH 7.4.

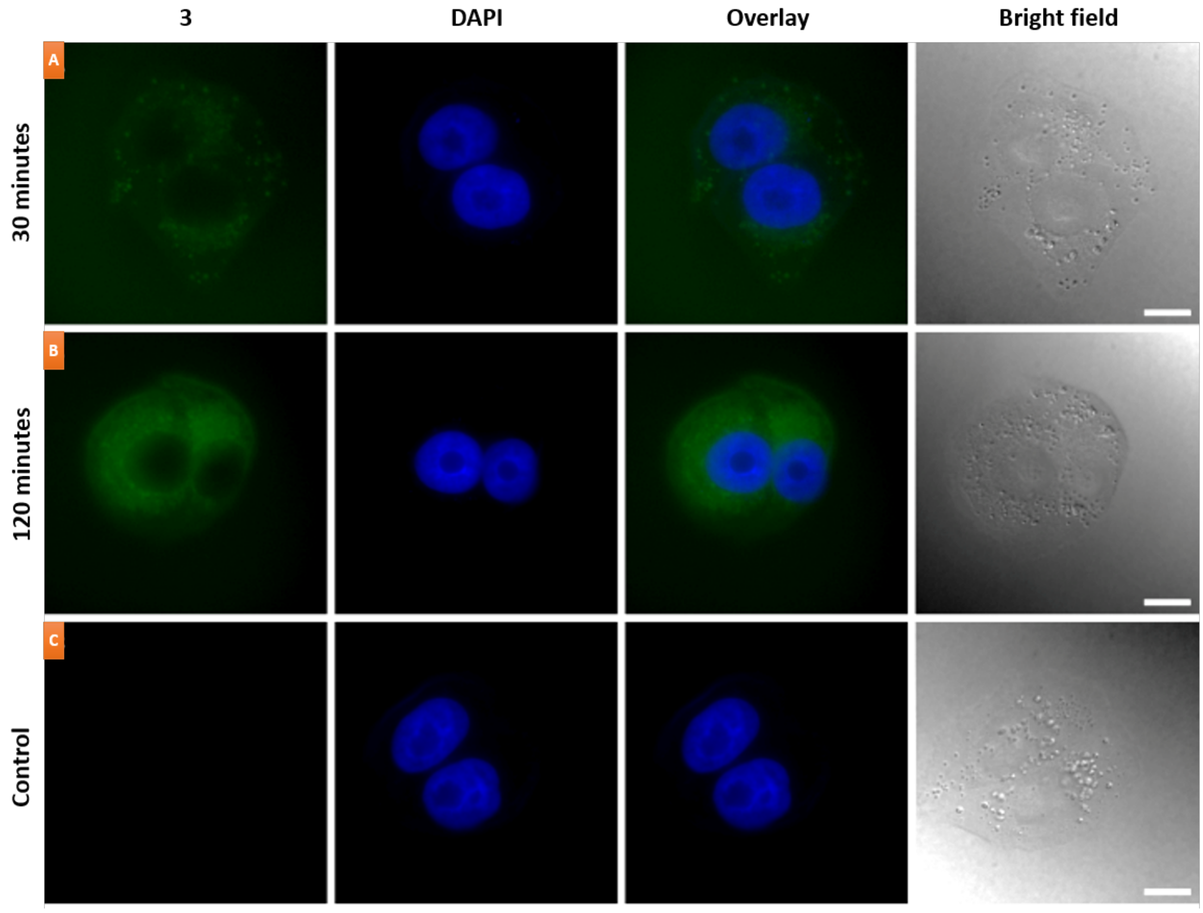

Figure S16. Fluorescence microscopy images of *HeLa* cells after incubation with **3** (5 µM) for (A) 30 min or (B) 120 min respectively. Control cells are shown in (C). Nuclear staining was performed using DAPI (blue fluorescence). Excitation was performed with the 350-450 and 492-518 filters for DAPI and **3**, respectively. A triple filter (437-474, 508-550 and 595-670 nm) was used to detect the fluorescence emission of both DAPI and **3**. Scale bar = 20 µm.

NMR Spectra

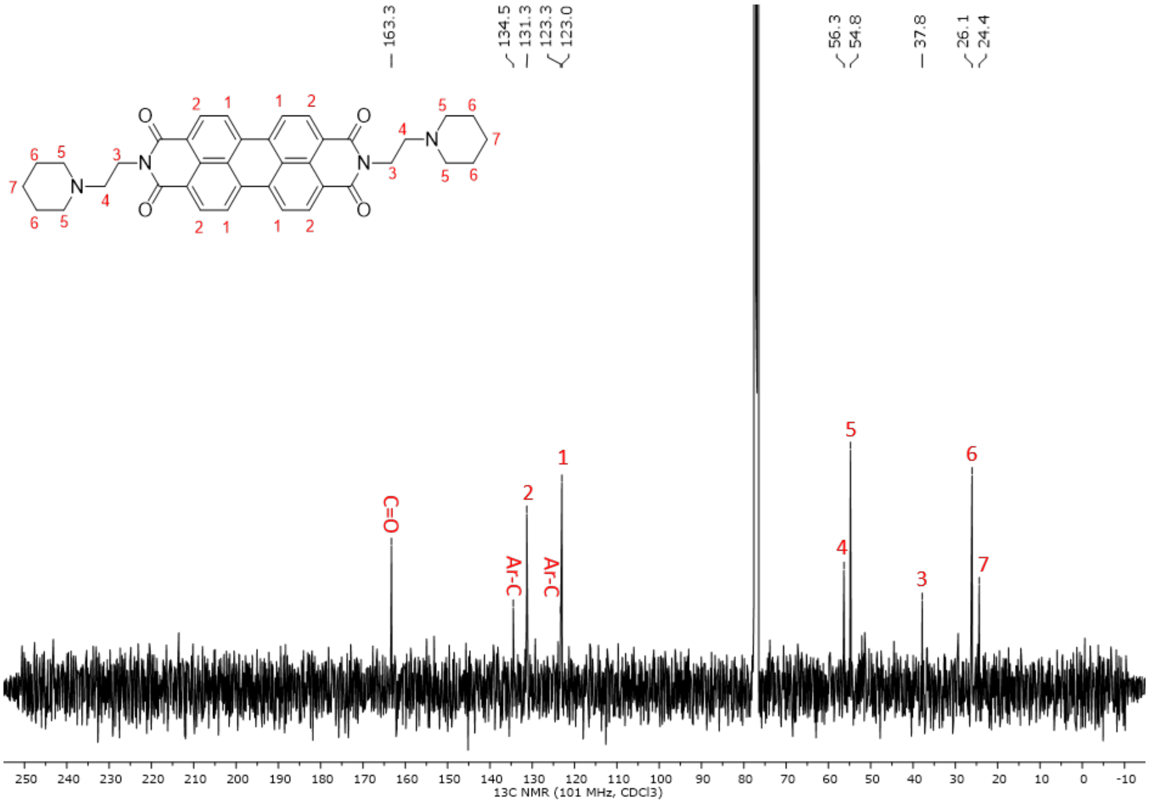

Figure S17. ^{13}C-NMR spectrum of PIPER in $CDCl_3$ (101 MHz).

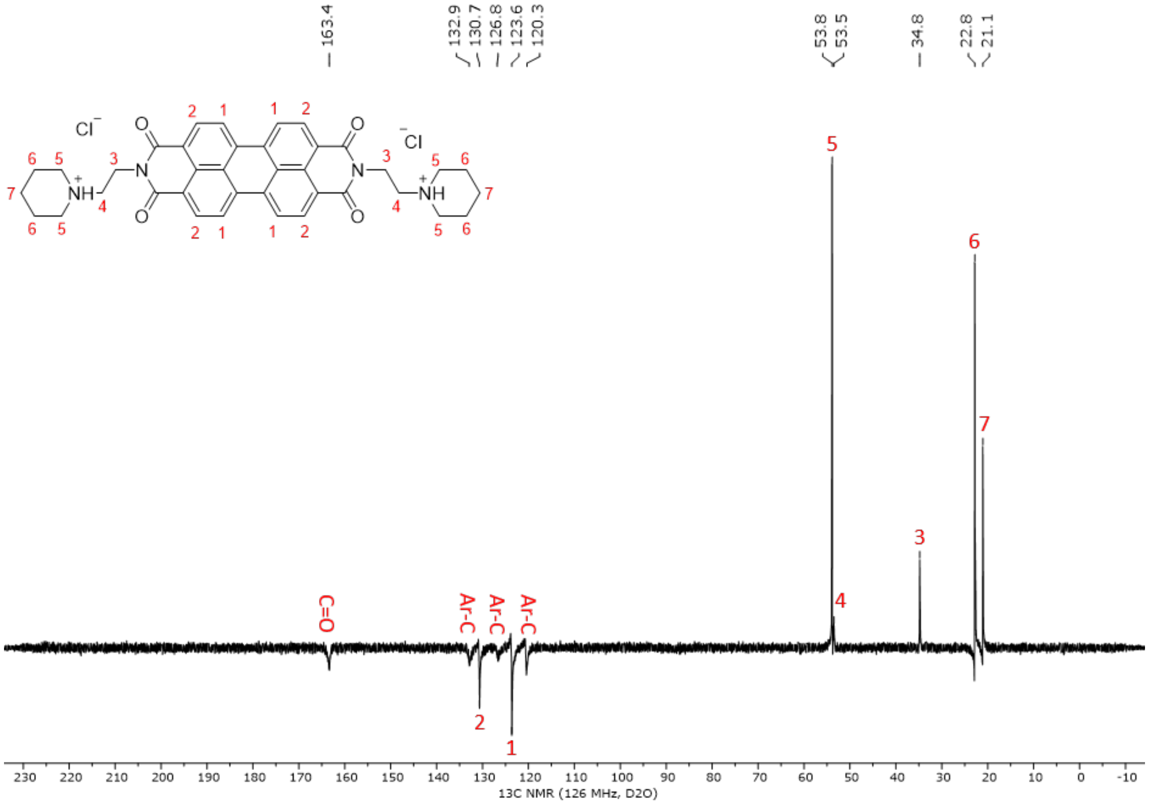

Figure S18. ^{13}C-NMR spectrum of PIPER · 2HCl in D_2O (126 MHz)

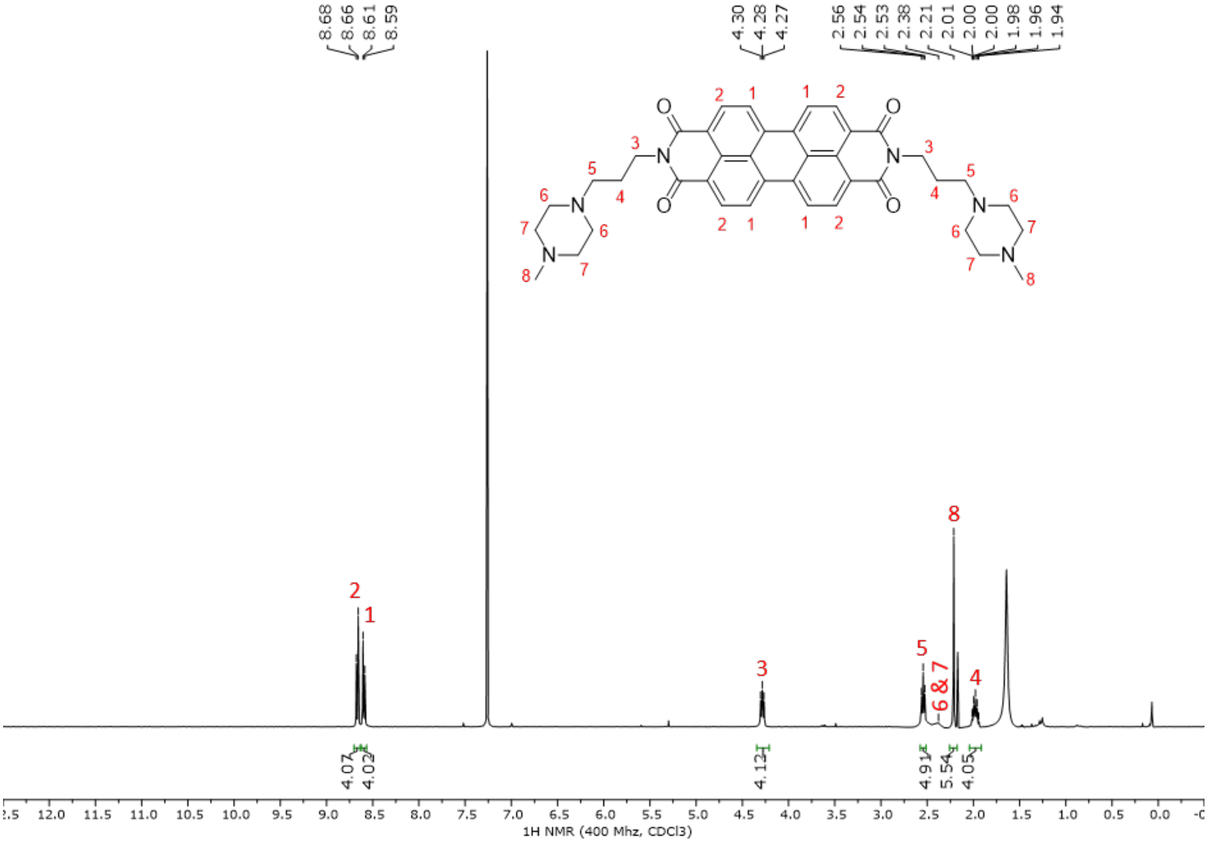

Figure S19. ^{1}H-NMR spectrum of **3** in $CDCl_3$ (400 MHz).

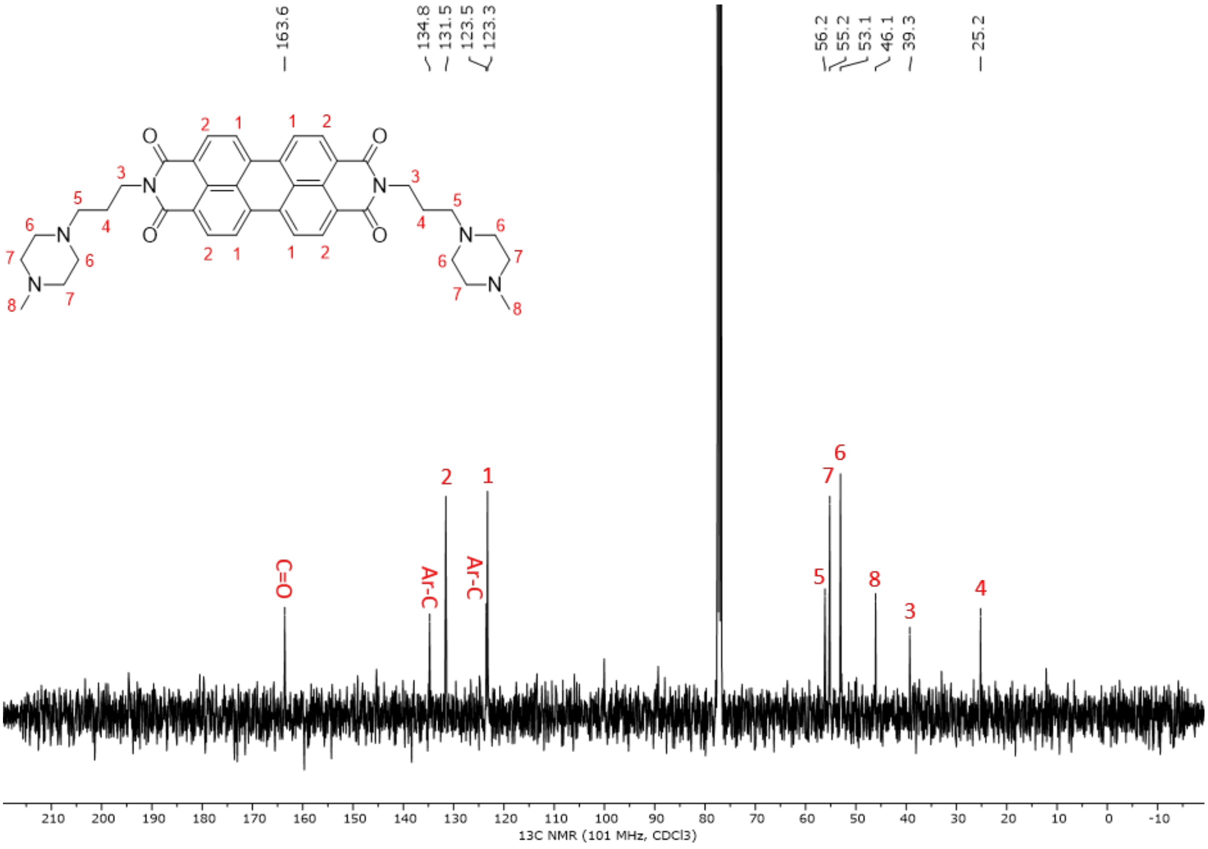

Figure S20. ^{13}C-NMR spectrum of **3** in $CDCl_3$ (101 MHz).

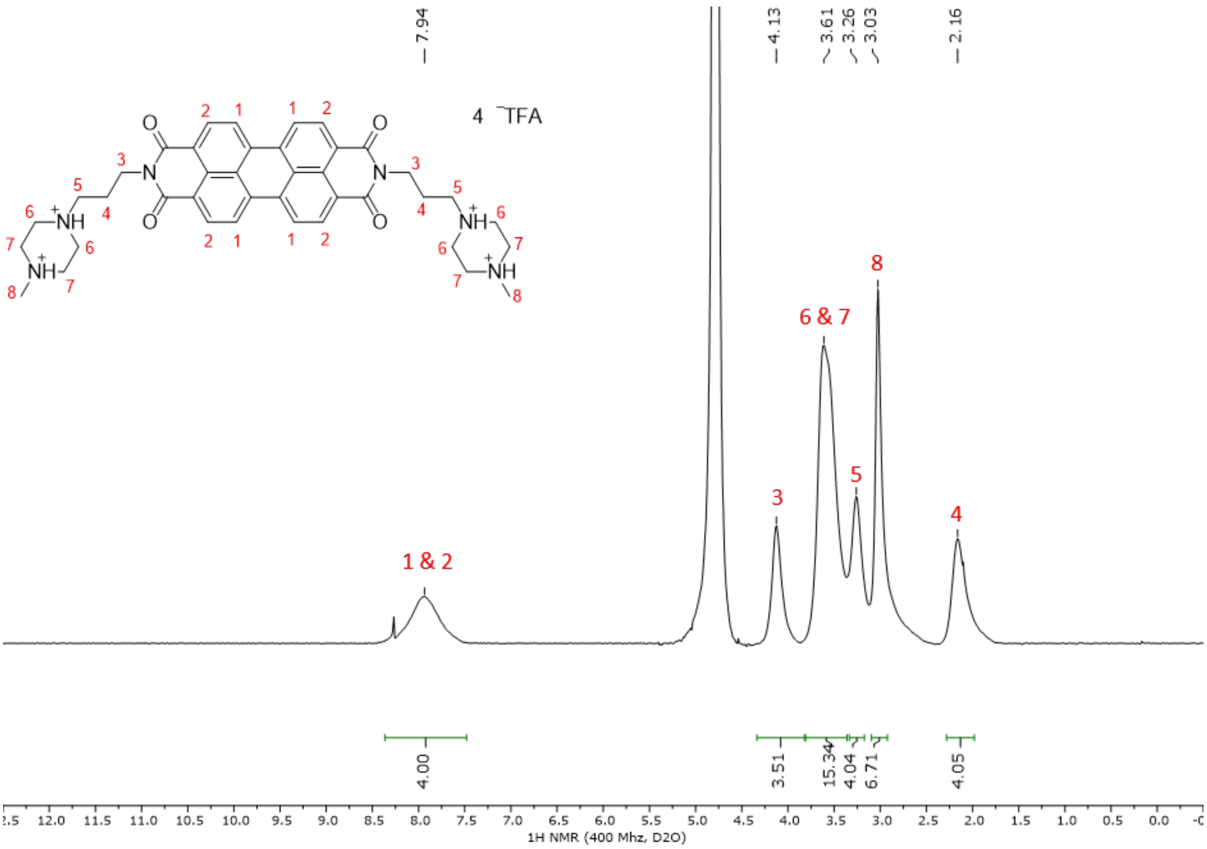

Figure S21. ^{1}H-NMR spectrum of **3** · 4TFA in D_2O (400 MHz).

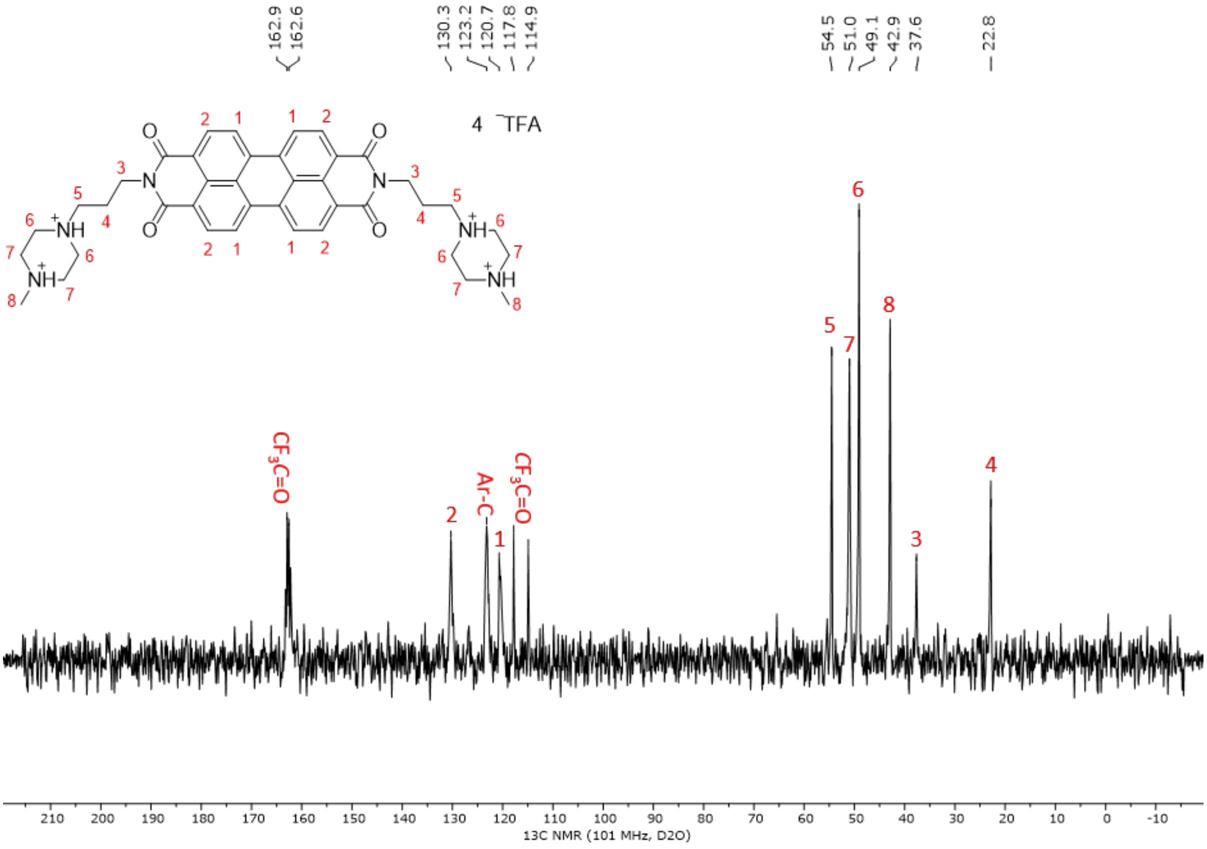

Figure S22. ^{13}C-NMR spectrum of **3** · 4TFA in D_2O (101 MHz).

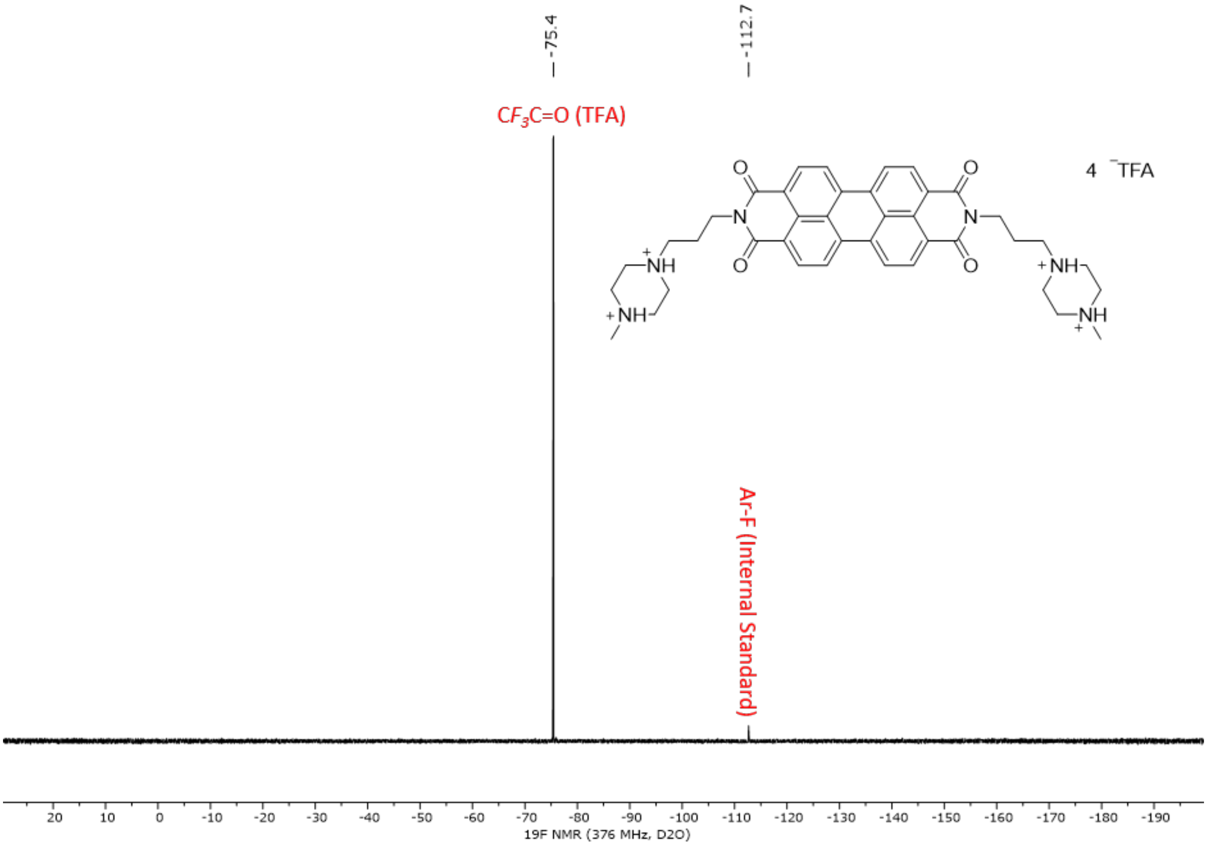

Figure S23. ^{19}F-NMR spectrum of **3** · 4TFA in D_2O (376 MHz).

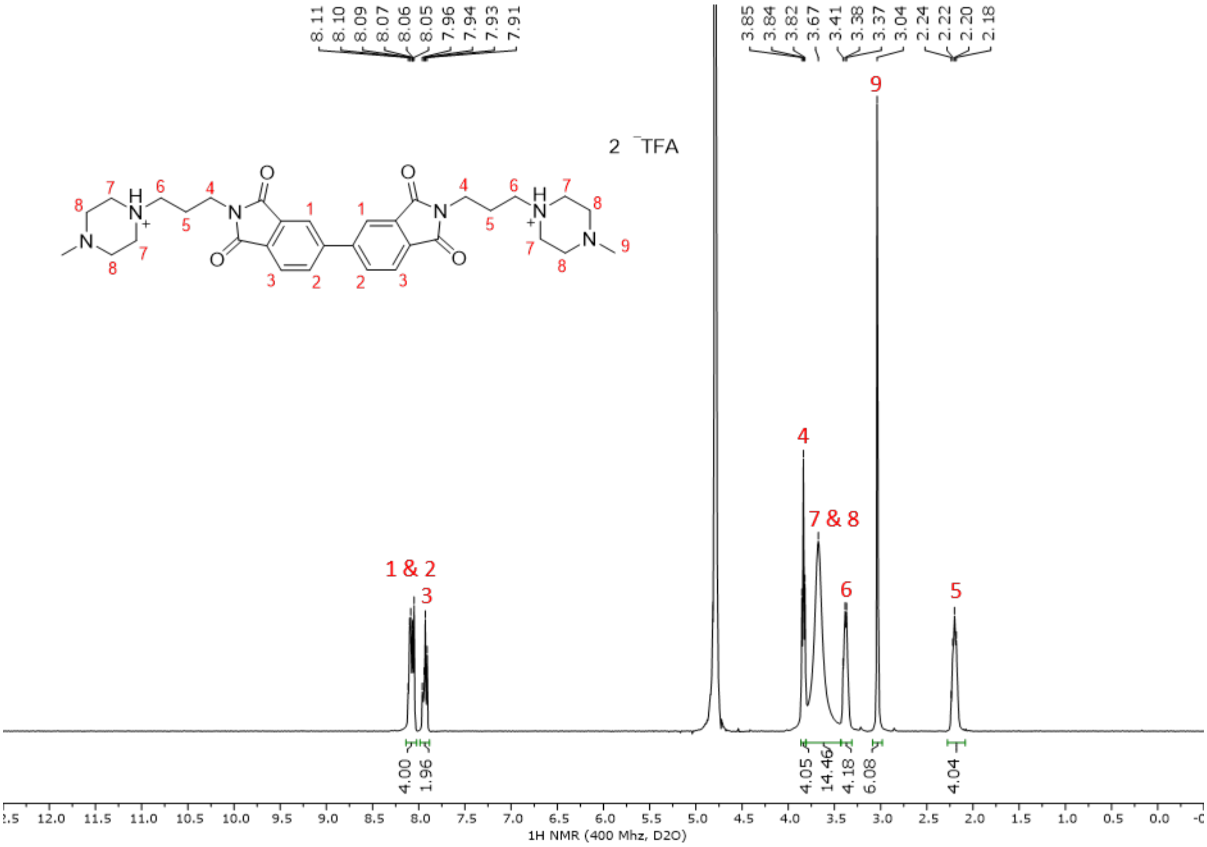

Figure S24. ^{1}H-NMR spectrum of **4** · 2TFA in D_2O (400 MHz).

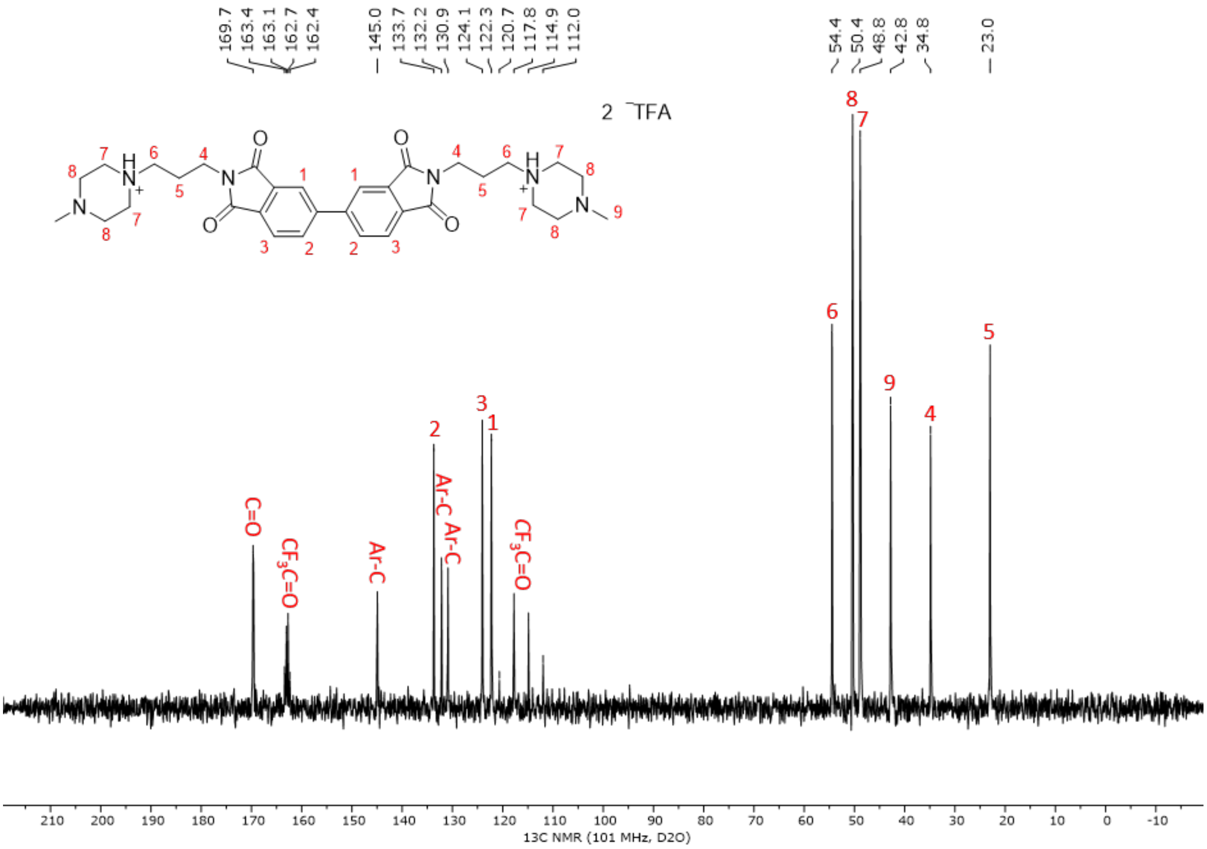

Figure S25. ^{13}C-NMR spectrum of **4** · 2TFA in D_2O (101 MHz).

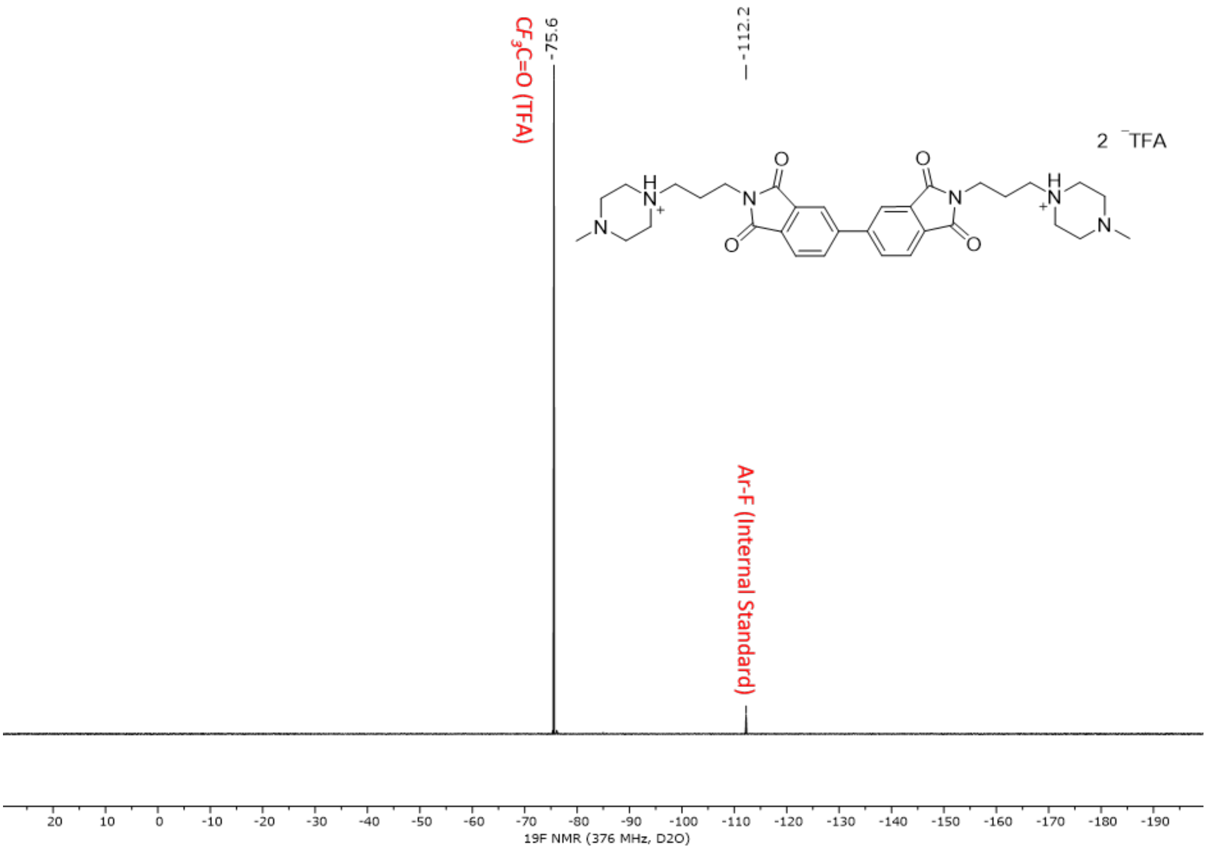

Figure S26. ^{19}F-NMR spectrum of **4** · 2TFA in D_2O (376 MHz).

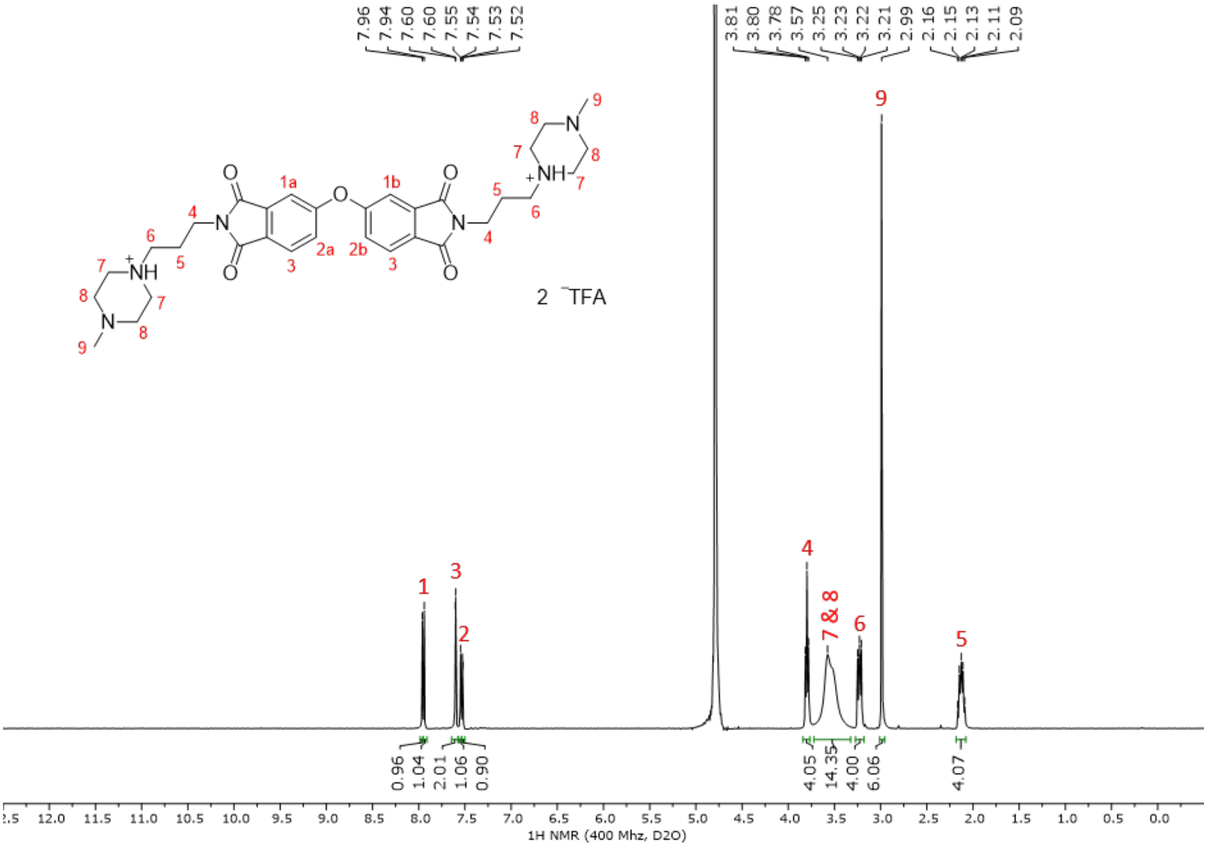

Figure S27. ^{1}H-NMR spectrum of **5** · 2TFA in D_2O (400 MHz).

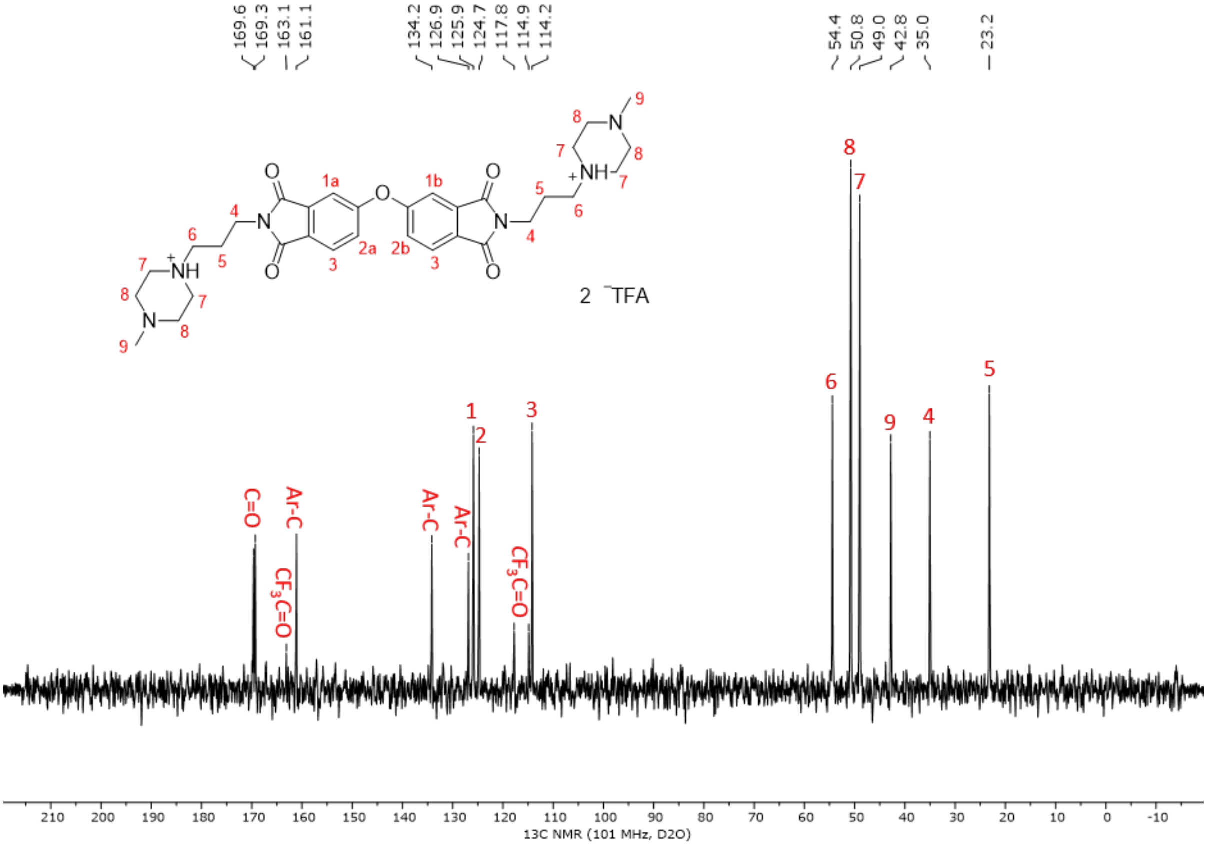

Figure S28. ^{13}C-NMR spectrum of **5** · 2TFA in D_2O (101 MHz).

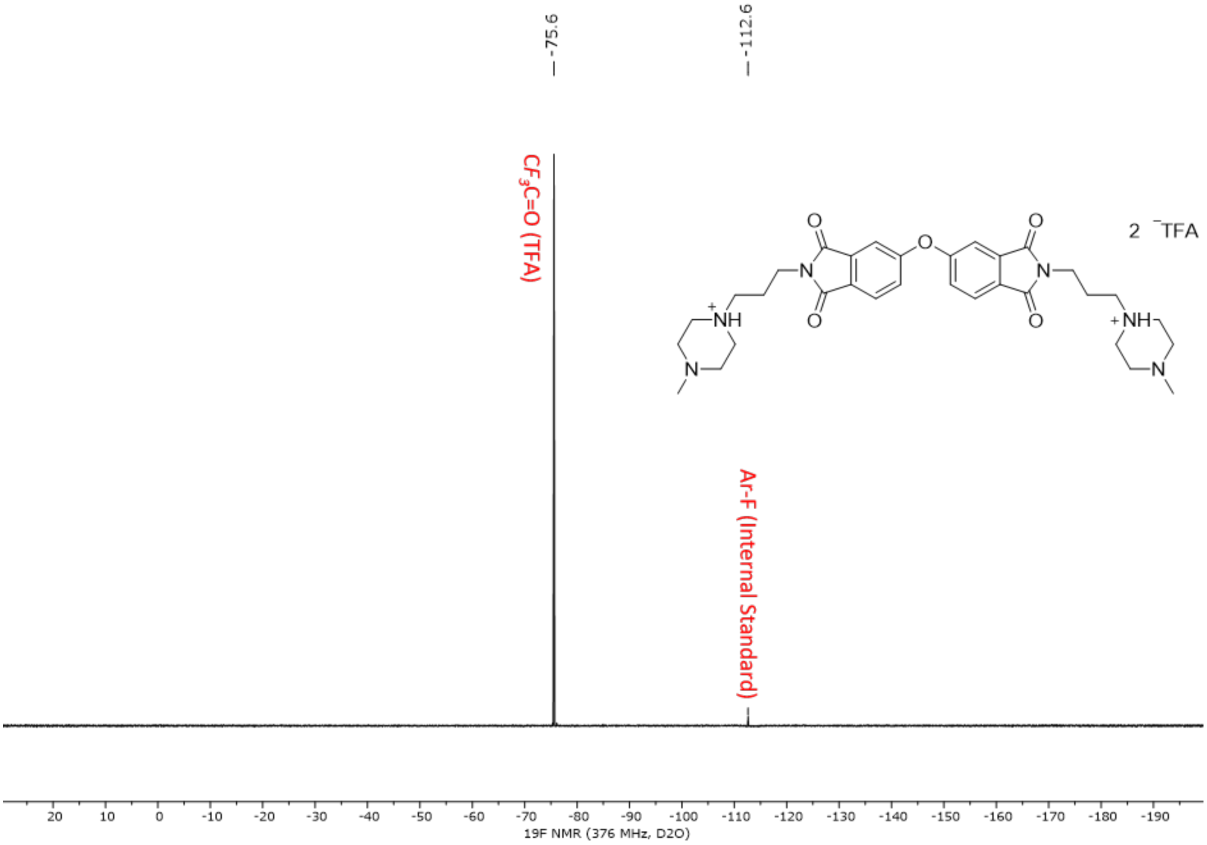

Figure S29. ^{19}F-NMR spectrum of **5** · 2TFA in D_2O (376 MHz).

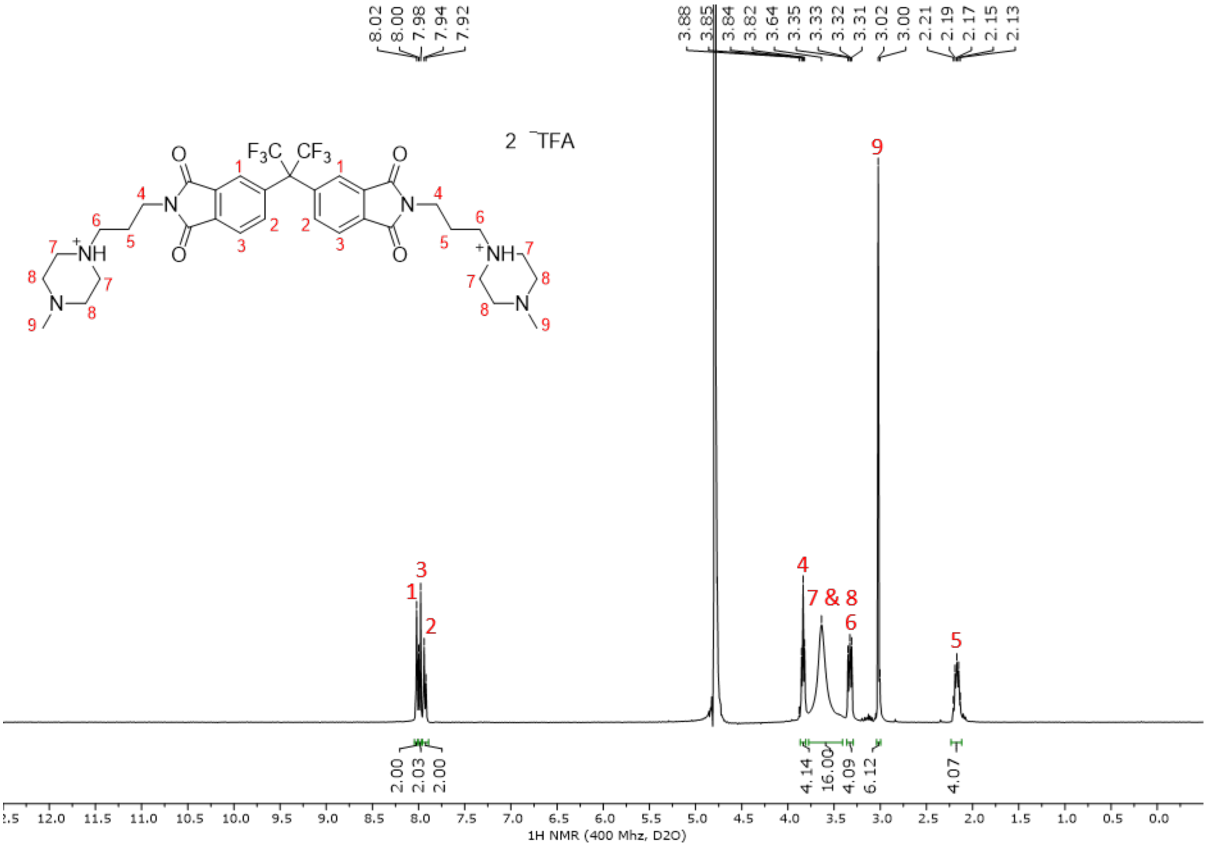

Figure S30. ^{1}H-NMR spectrum of **6** · 2TFA in D_2O (400 MHz).

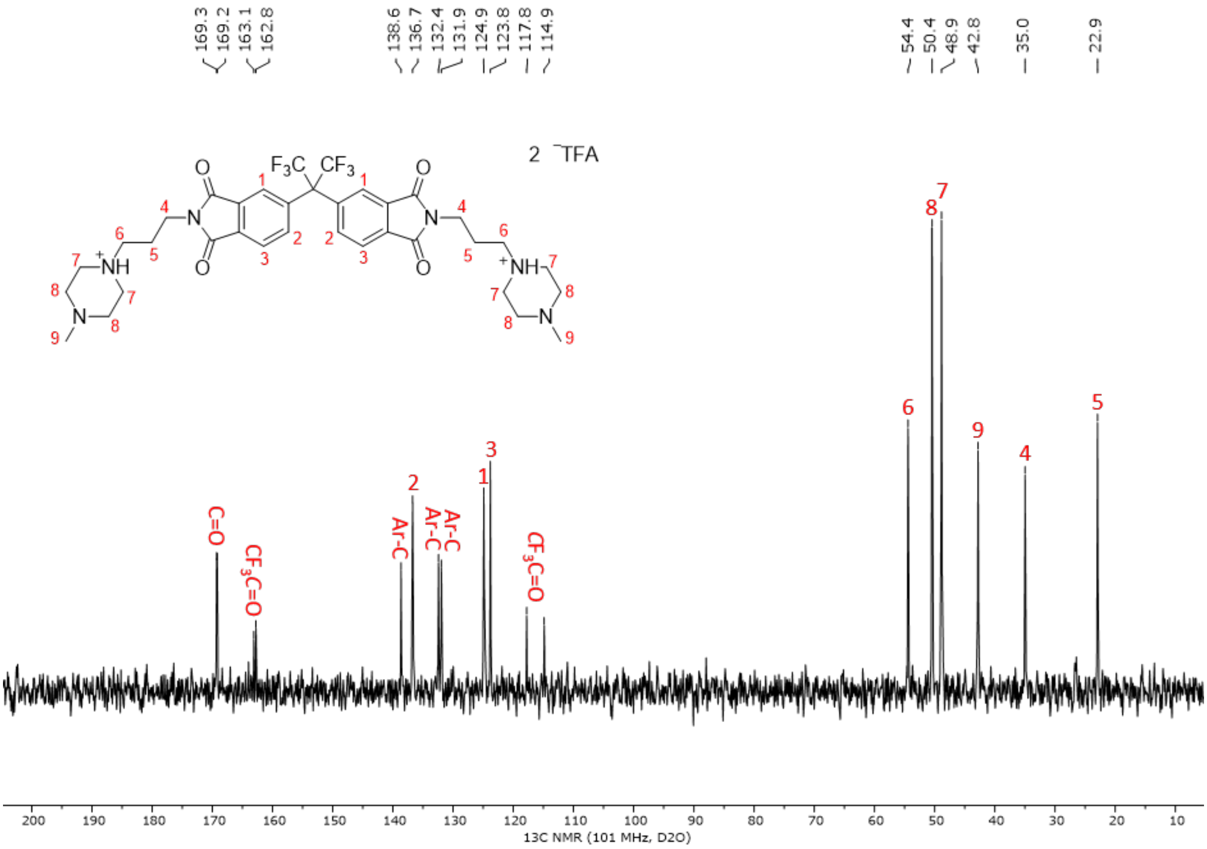

Figure S31. ^{13}C-NMR spectrum of **6** · 2TFA in D_2O (101 MHz).

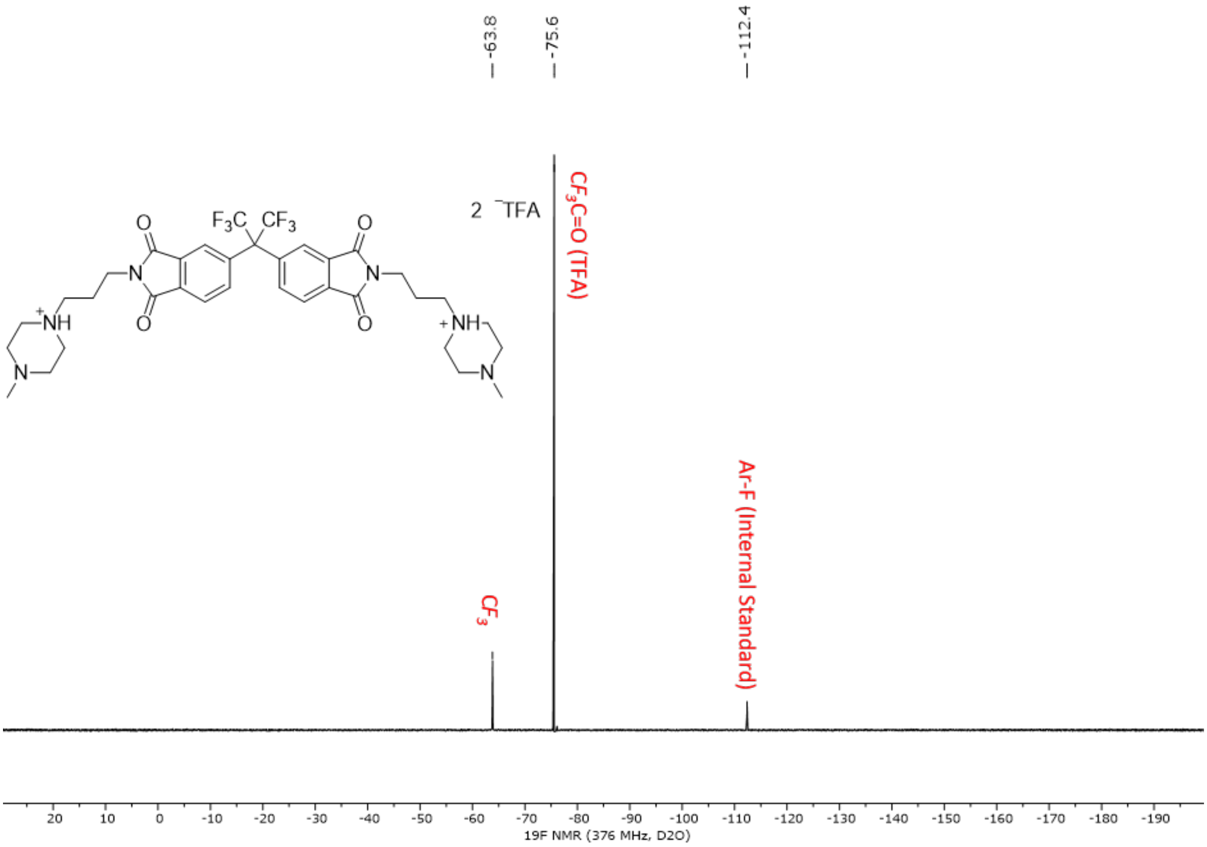

Figure S32. ^{19}F-NMR spectrum of **6** · 2TFA in D_2O (376 MHz).

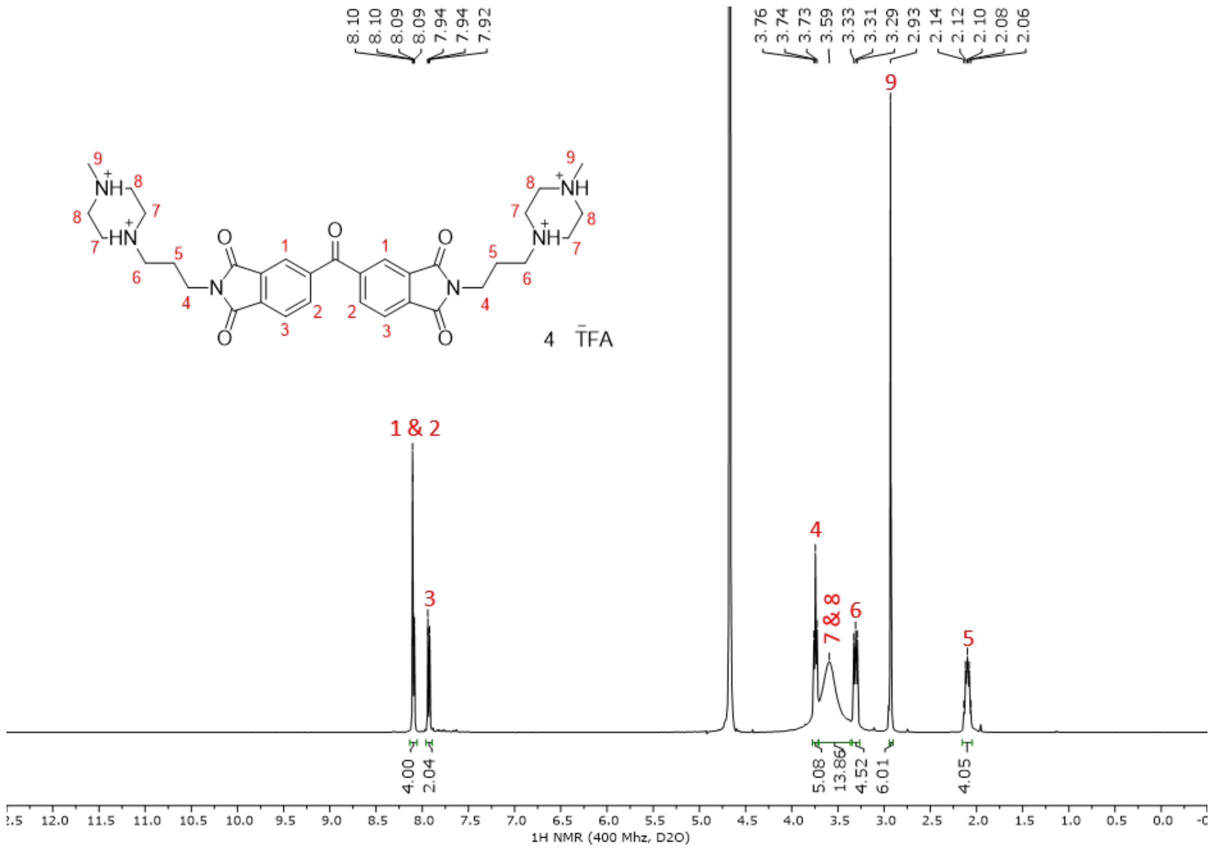

Figure S33. ^{1}H-NMR spectrum of **7** · 4TFA in D_2O (400 MHz).

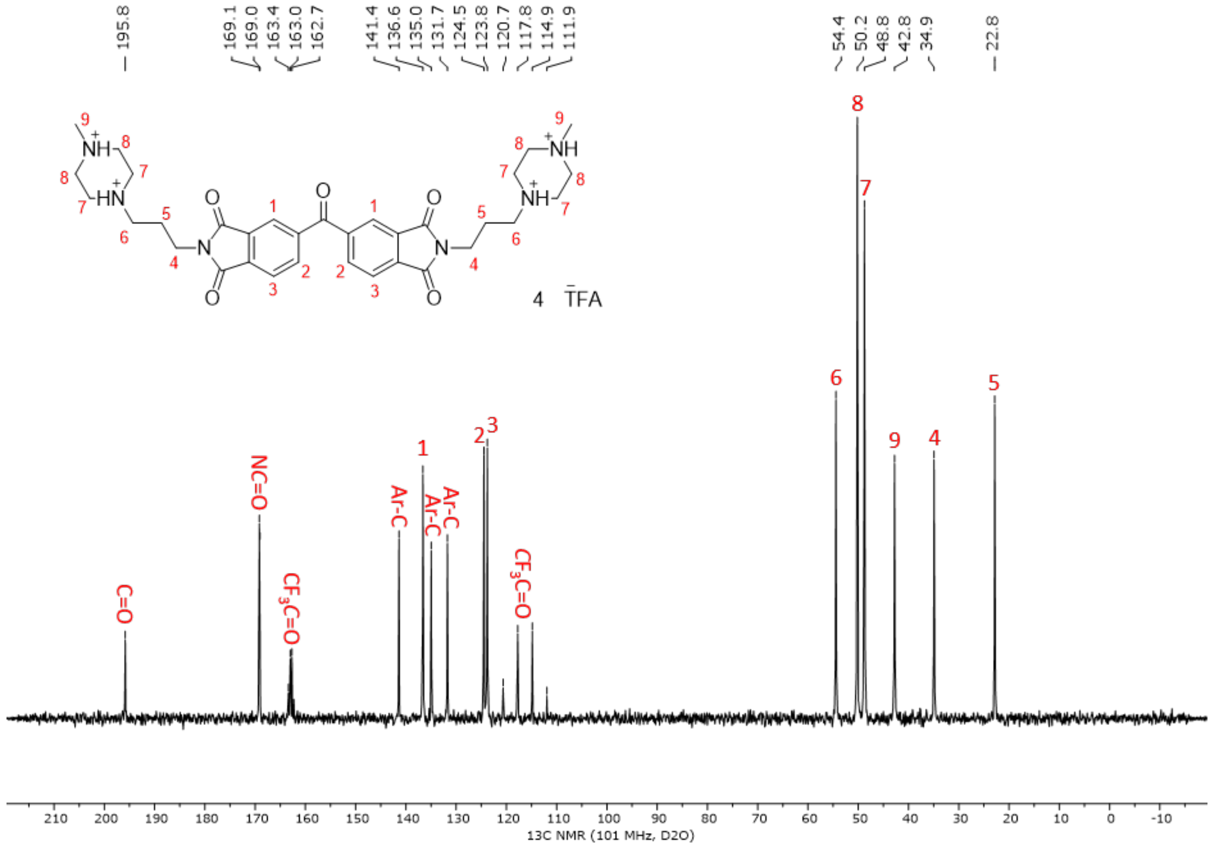

Figure S34. ^{13}C-NMR spectrum of **7** · 4TFA in D_2O (101 MHz).

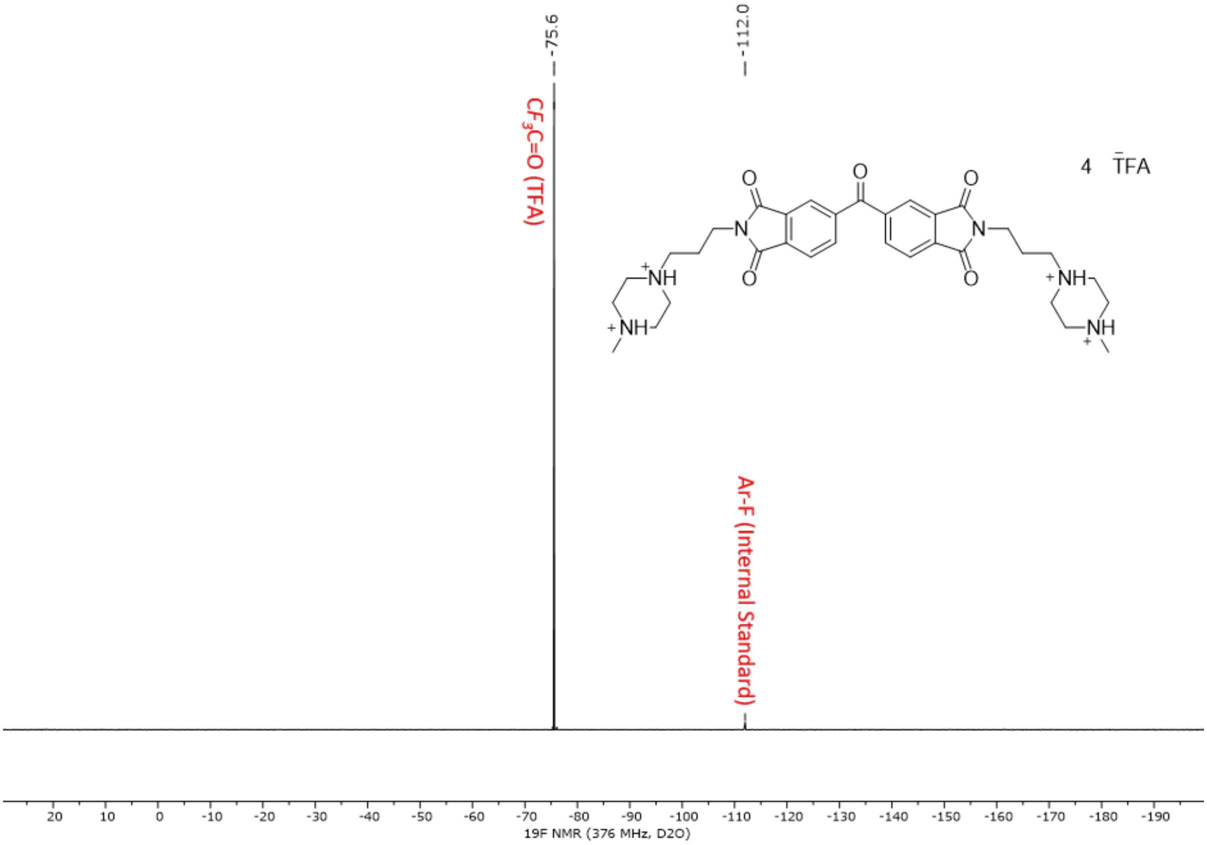

Figure S35. ^{19}F-NMR spectrum of **7** · 4TFA in D_2O (376 MHz).

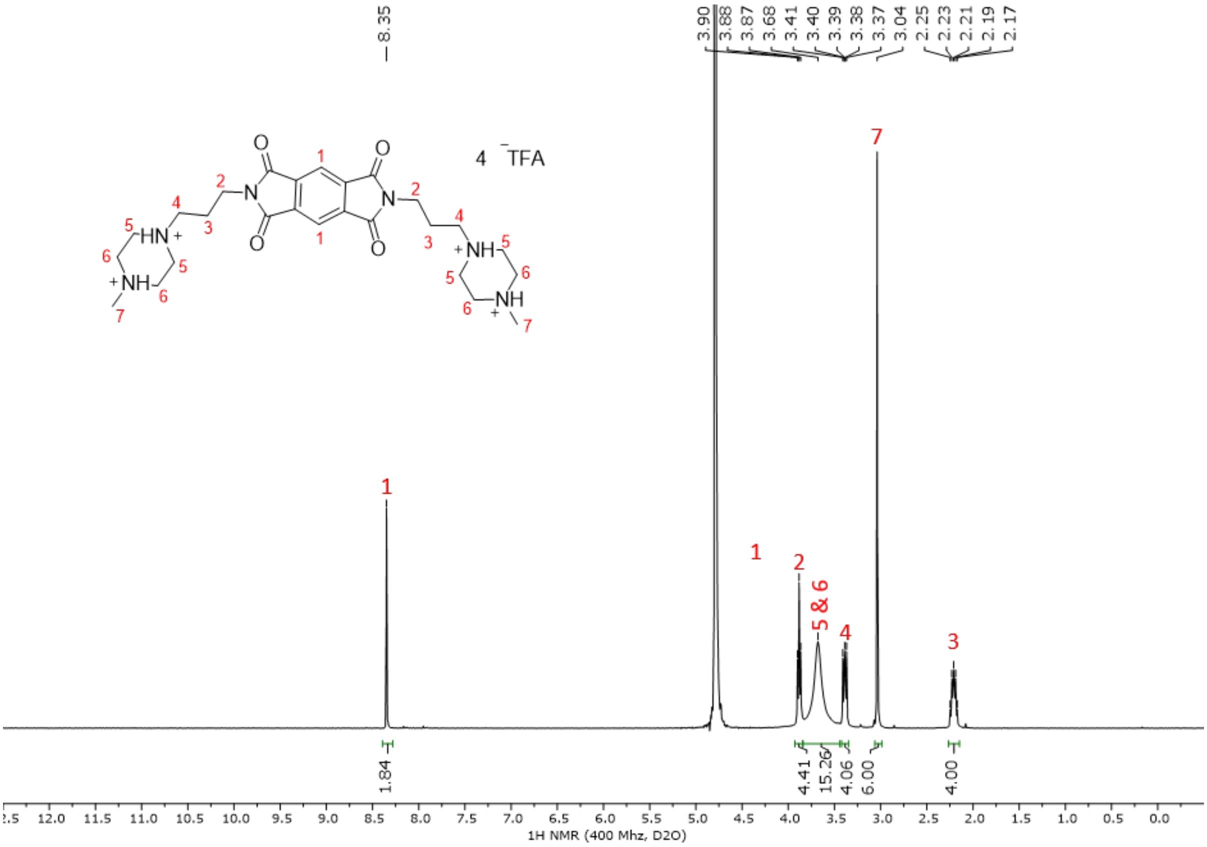

Figure S36. ^{1}H-NMR spectrum of **8** · 4TFA in D_2O (400 MHz).

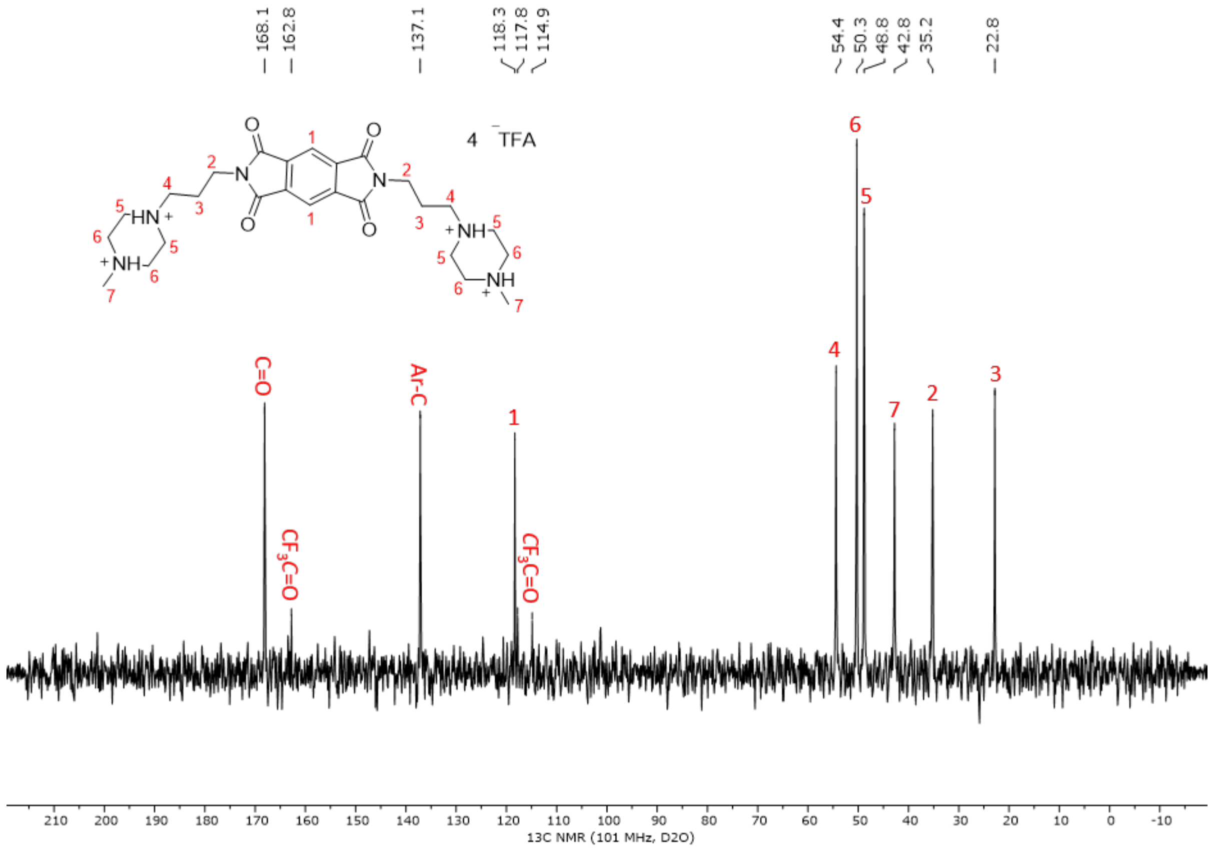

Figure S37. ^{13}C-NMR spectrum of **8** · 4TFA in D_2O (101 MHz).

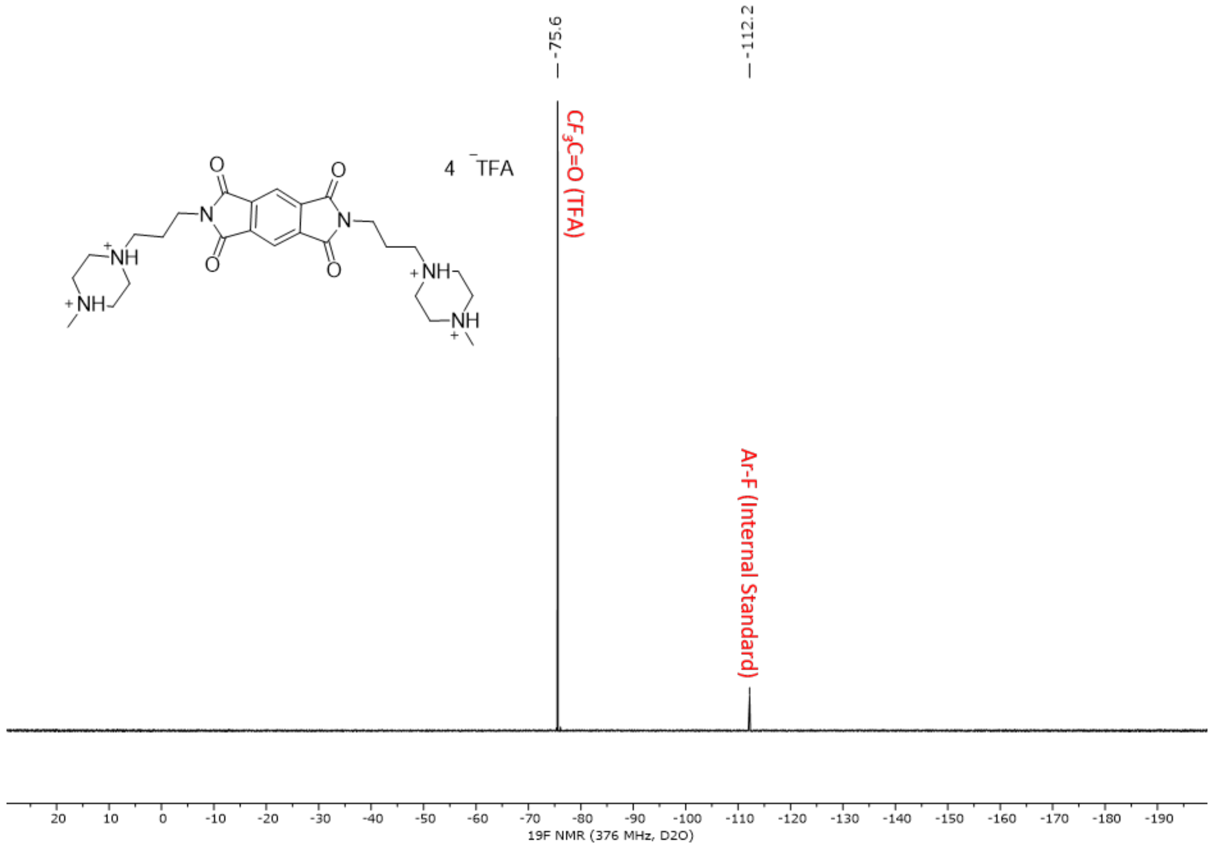

Figure S38. ^{19}F-NMR spectrum of **8** · 4TFA in D_2O (376 MHz).

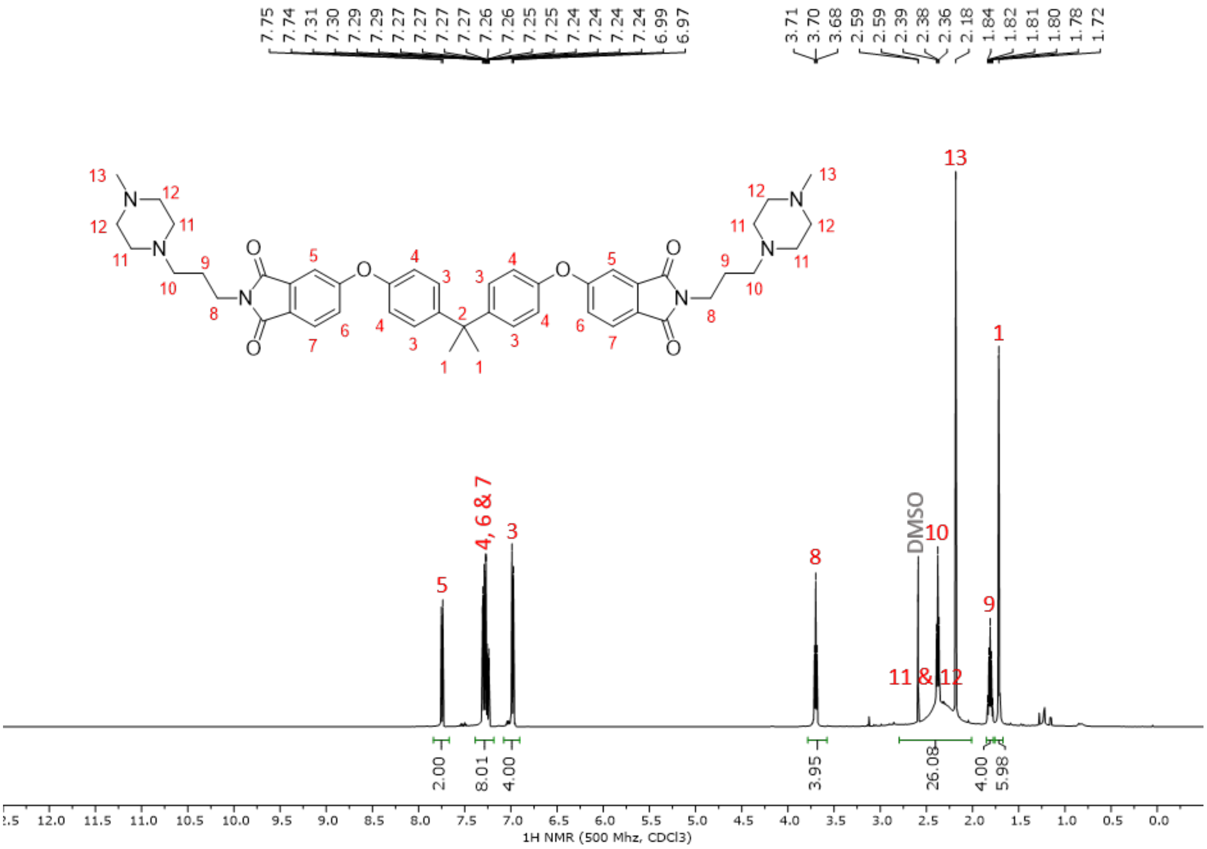

Figure S39. ^{1}H-NMR spectrum of **9** in $CDCl_3$ (500 MHz).

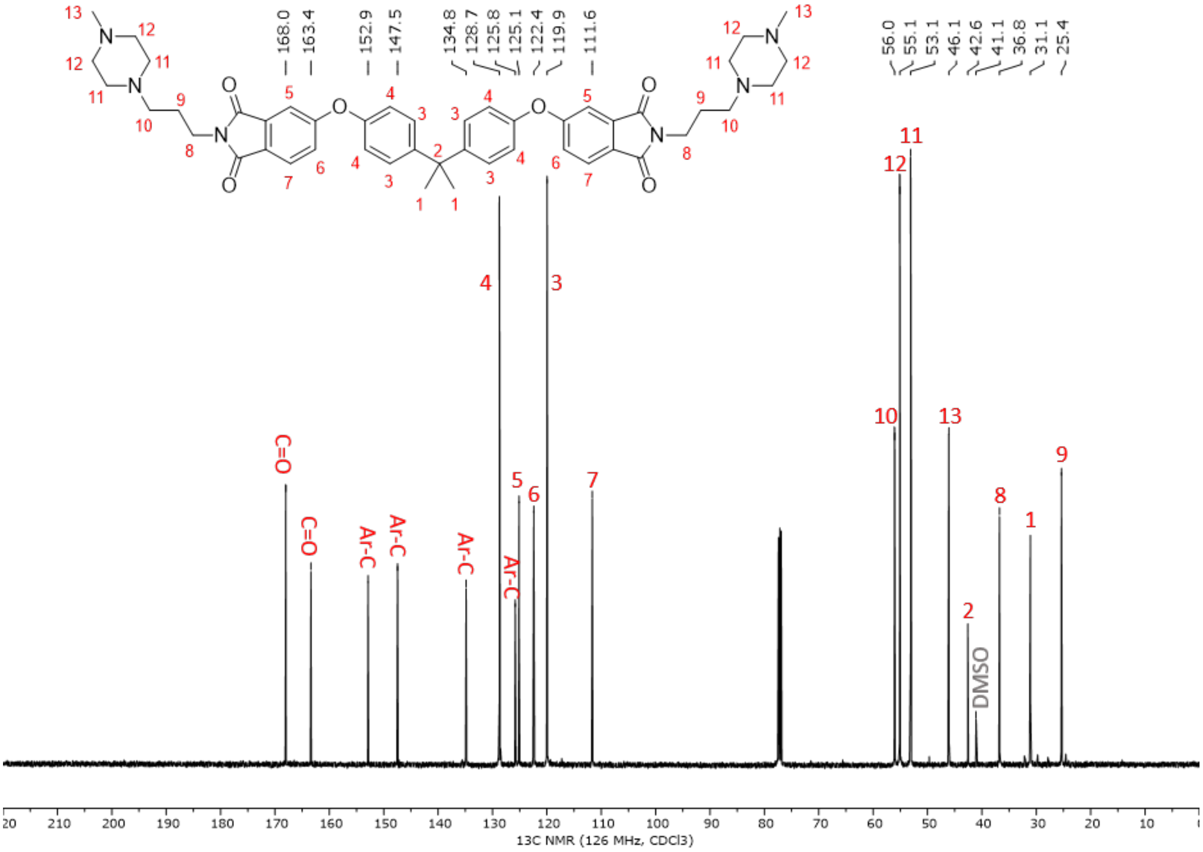

Figure S40. ^{13}C-NMR spectrum of **9** in $CDCl_3$ (126 MHz).

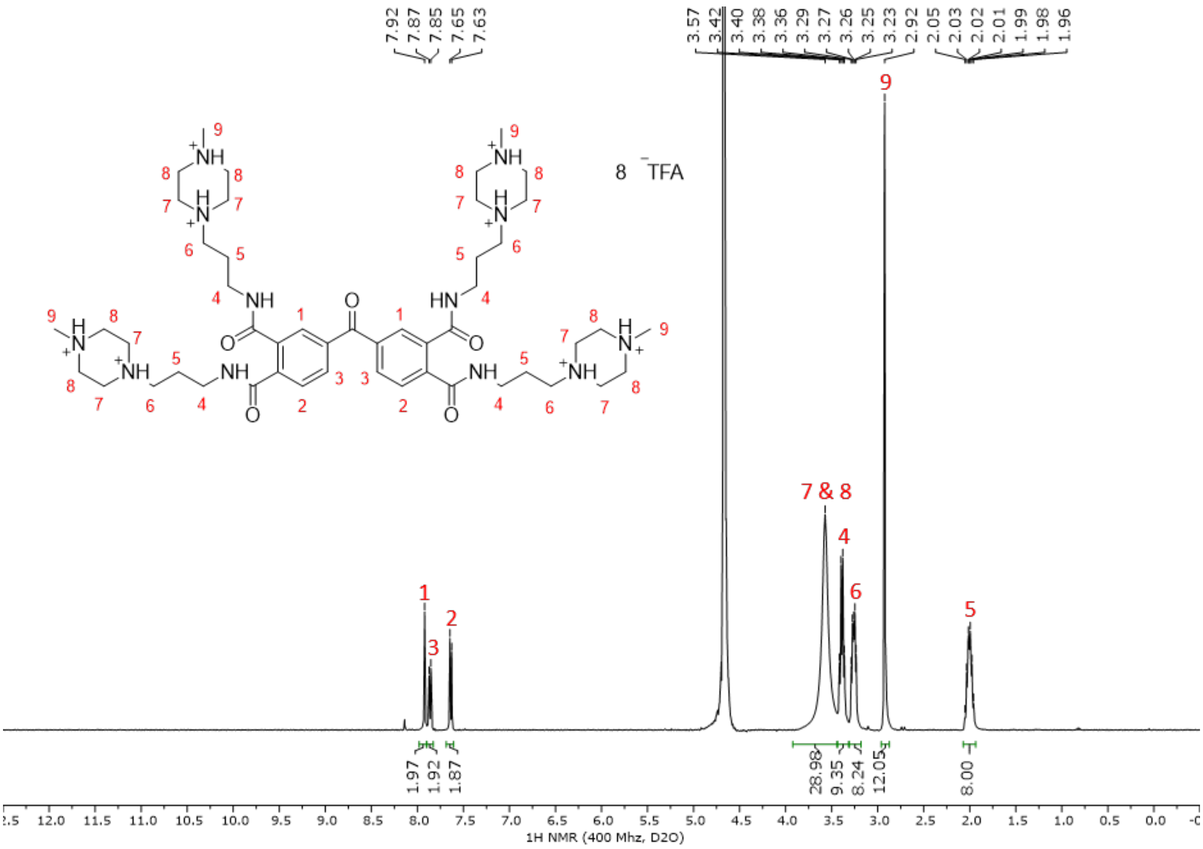

Figure S41. ^{1}H-NMR spectrum of **10** · 8TFA in D_2O (400 MHz).

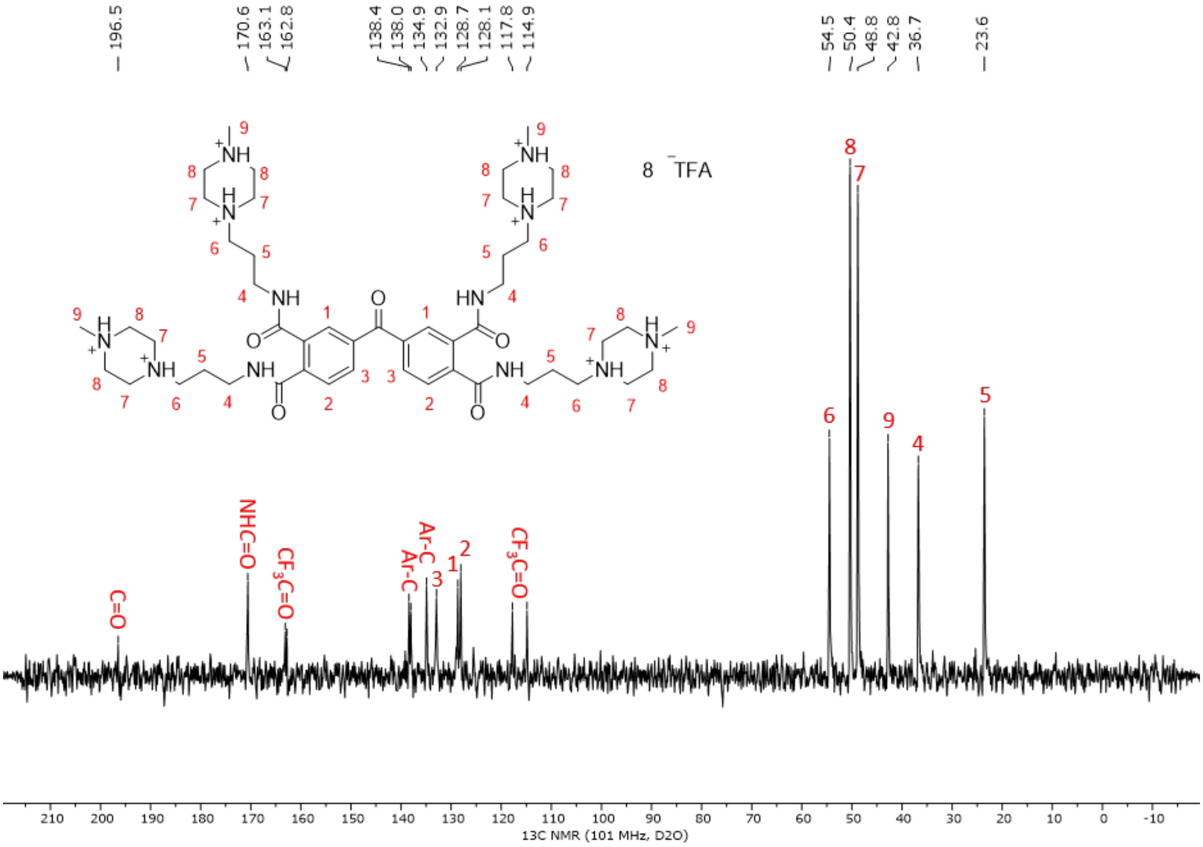

Figure S42. ^{13}C-NMR spectrum of **10** · 8TFA in D_2O (101 MHz).

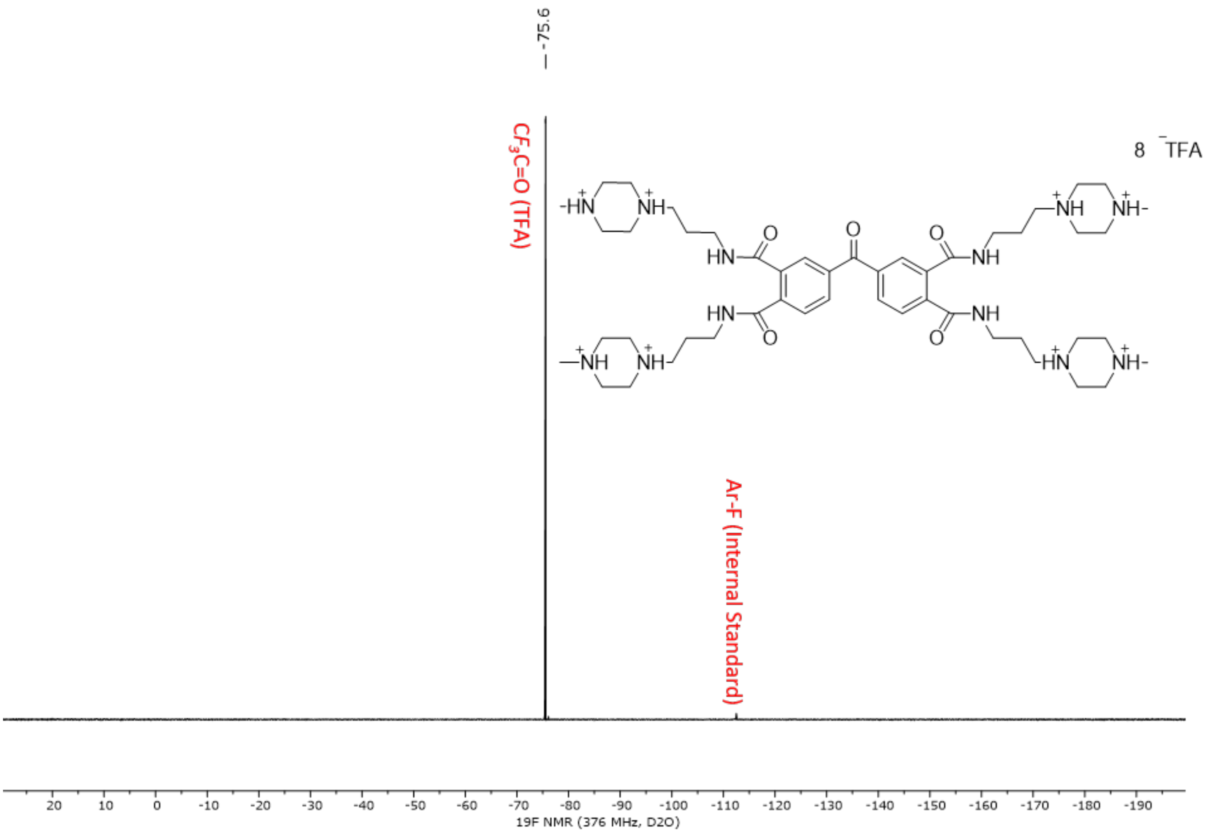

Figure S43. ^{19}F-NMR spectrum of **10** · 8TFA in D_2O (376 MHz).

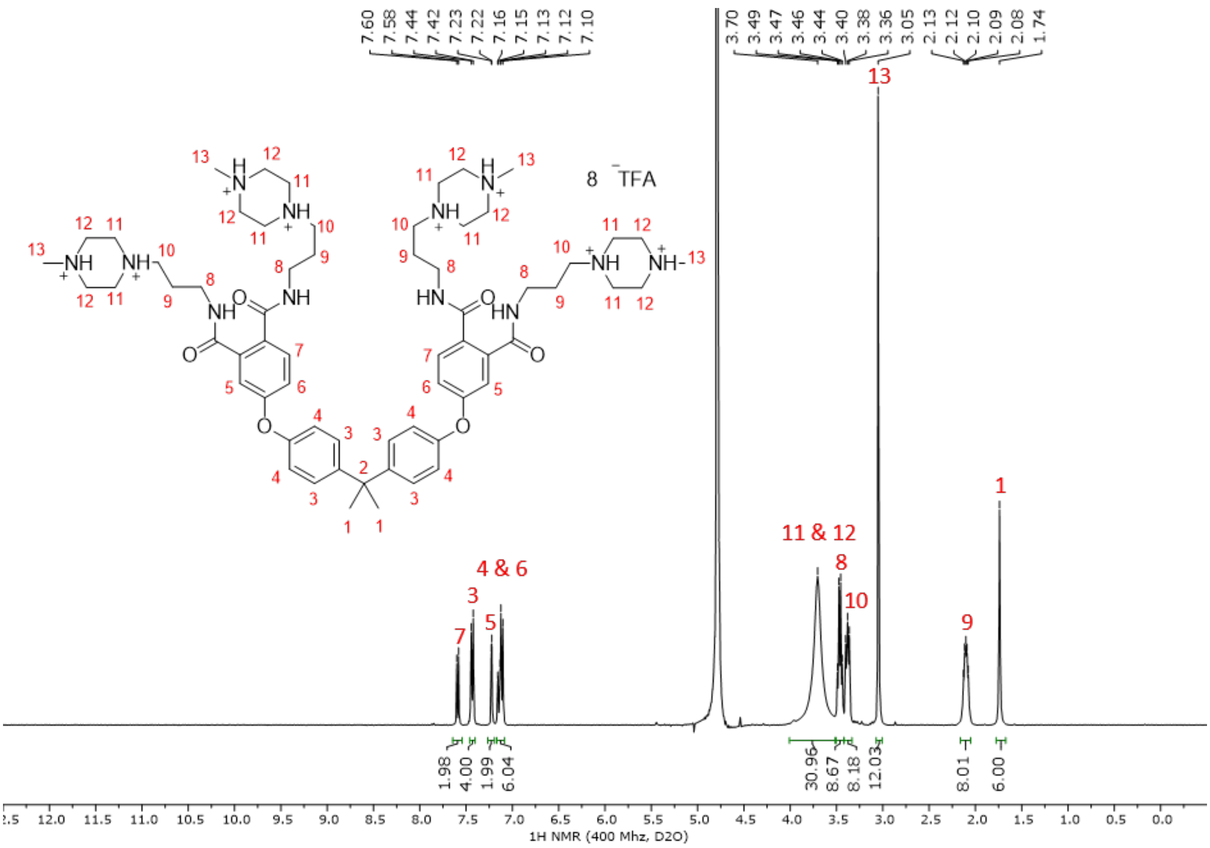

Figure S44. ^{1}H-NMR spectrum of **11** · 8TFA in D_2O (400 MHz).

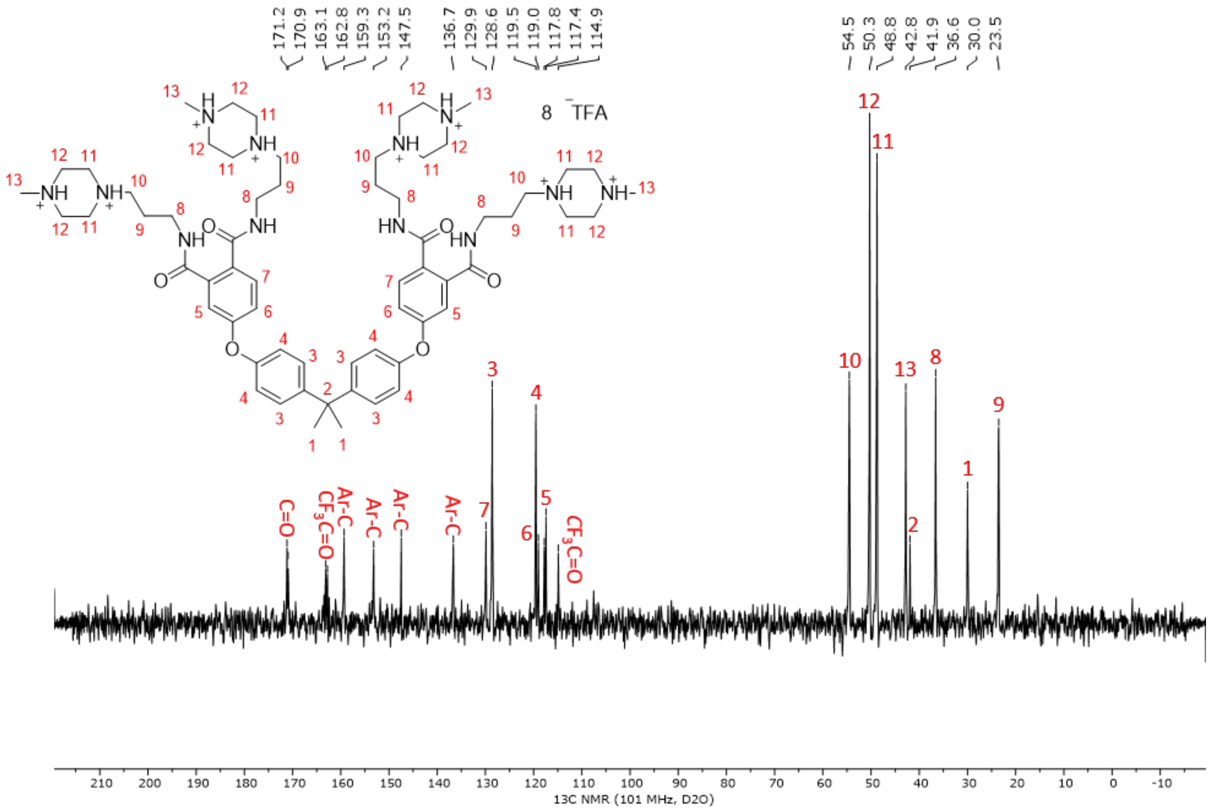

Figure S45. ^{13}C-NMR spectrum of **11** · 8TFA in D_2O (101 MHz).

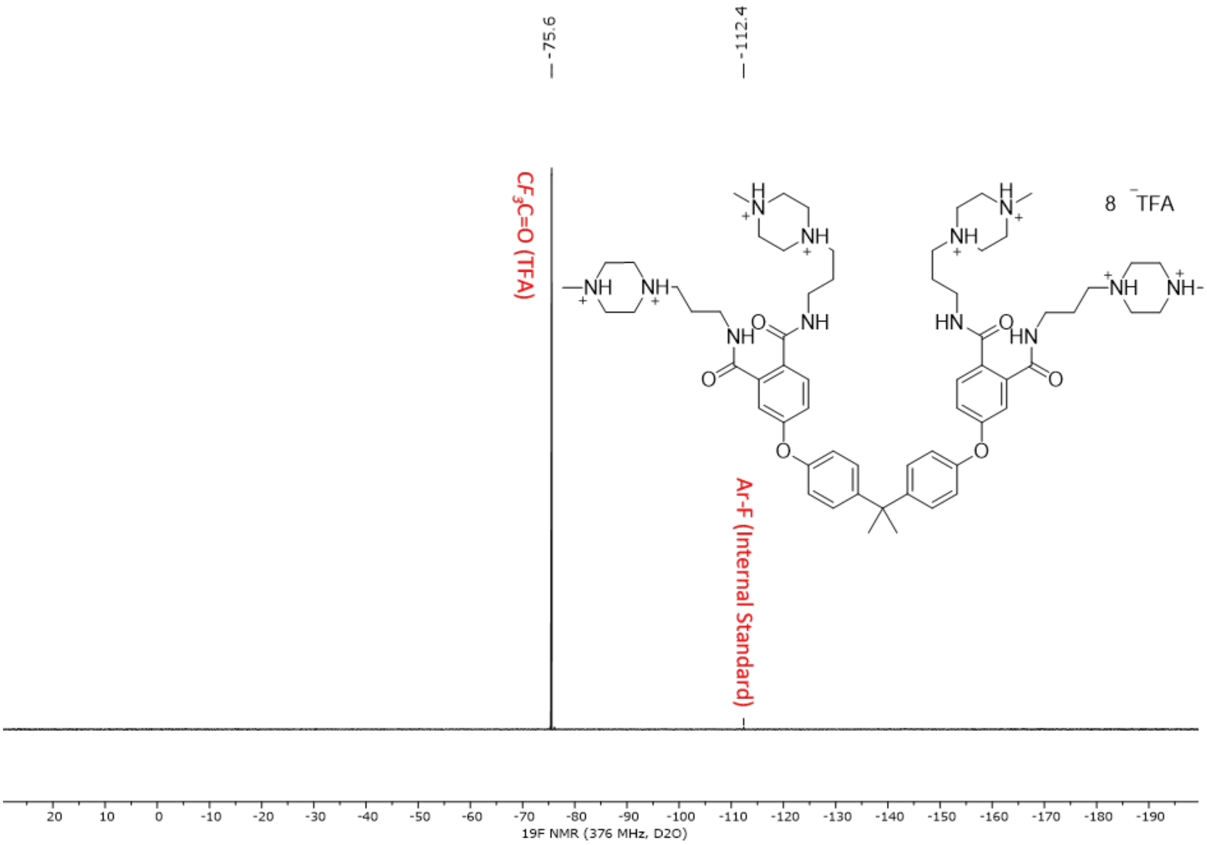

Figure S46. ^{19}F-NMR spectrum of **11** · 8TFA in D_2O (376 MHz).

References

[S1] A. B. Pangborn, M. A. Giardello, R. H. Grubbs, R. K. Rosen, F. J. Timmers, *Organometallics* **1996**, *15*, 1518–1520.

[S2] W. C. Still, M. Kahn, A. Mitra, *J. Org. Chem.* **1978**, *43*, 2923–2925.

[S3] A. De Cian, L. Guittat, M. Kaiser, B. Saccà, S. Amrane, A. Bourdoncle, P. Alberti, M.-P. Teulade-Fichou, L. Lacroix, J.-L. Mergny, *Methods* **2007**, *42*, 183–195.

[S4] J. L. Mergny, J. C. Maurizot, *ChemBioChem* **2001**, *2*, 124–132.

[S5] A. Ambrus, D. Chen, J. Dai, R. A. Jones, D. Yang, *Biochemistry* **2005**, *44*, 2048–58.

[S6] E. Belmonte-Reche, M. Martínez-García, A. Guédin, M. Zuffo, M. Arévalo-Ruiz, F. Doria, J. Campos-Salinas, M. Maynadier, J. J. López-Rubio, M. Freccero, J.-L. Mergny, J. M. Pérez-Victoria, J. C. Morales, *J. Med. Chem.* **2018**, *61*, 1231–1240.

[S7] A. D. Moorhouse, A. M. Santos, M. Gunaratnam, M. Moore, S. Neidle, J. E. Moses, *J. Am. Chem. Soc.* **2006**, *128*, 15972–15973.

[S8] K. N. Luu, A. T. Phan, V. Kuryavyi, L. Lacroix, D. J. Patel, *J. Am. Chem. Soc.* **2006**, *128*, 9963–9970.

[S9] Y. Wang, D. J. Patel, *Structure* **1993**, *1*, 263–282.

[S10] J. L. Mergny, L. Lacroix, M. P. Teulade-Fichou, C. Hounsou, L. Guittat, M. Hoarau, P. B. Arimondo, J. P. Vigneron, J. M. Lehn, J. F. Riou, T. Garestier, C. Hélène, *Proc. Natl. Acad. Sci. U. S. A.* **2001**, *98*, 3062–7.

[S11] P. Thordarson, *Chem. Soc. Rev.* **2011**, *40*, 1305–1323.

[S12] L. Hahn, N. J. Buurma, L. H. Gade, *Chem. Eur. J.* **2016**, *22*, 6314–6322.

[S13] E. M. Larson, D. J. Doughman, D. S. Gregerson, W. F. Obritsch, *Investig. Ophthalmol. Vis. Sci.* **1997**, *38*, 1929–1933.

[S14] J. M. Pérez-Victoria, B. I. Bavchvarov, I. R. Torrecillas, M. Martínez-García, C. López-Martín, M. Campillo, S. Castanys, F. Gamarro, *Antimicrob. Agents Chemother.* **2011**, *55*, 3838–3844.

[S15] C. Sissi, L. Lucatello, A. P. Krapcho, D. J. Maloney, M. B. Boxer, M. V. Camarasa, G. Pezzoni, E. Menta, M. Palumbo, *Bioorg. Med. Chem.* **2007**, *15*, 555–562.

[S16] S. T. G. Street, D. N. Chin, G. J. Hollingworth, M. Berry, J. C. Morales, M. C. Galan, *Chem. Eur. J.* **2017**, *23*, 6953–6958.
